# Comparative analysis of the efficacies of probiotic supplementation and glucose-lowering drugs for the treatment of type 2 diabetes: A systematic review and meta-analysis

**DOI:** 10.3389/fnut.2022.825897

**Published:** 2022-07-18

**Authors:** Tingting Liang, Xinqiang Xie, Lei Wu, Longyan Li, Lingshuang Yang, He Gao, Zhenshan Deng, Xiangqian Zhang, Xuefeng Chen, Jumei Zhang, Yu Ding, Qingping Wu

**Affiliations:** ^1^School of Food and Biological Engineering, Shaanxi University of Science and Technology, Xi'an, China; ^2^Guangdong Provincial Key Laboratory of Microbial Safety and Health, State Key Laboratory of Applied Microbiology Southern China, Guangdong Institute of Microbiology, Guangdong Academy of Sciences, Guangzhou, China; ^3^College of Life Sciences, Yan'an University, Yan'an, China; ^4^Department of Food Science & Technology, Institute of Food Safety and Nutrition, Jinan University, Guangzhou, China

**Keywords:** probiotics, glucose-lowering drugs, glycemic, lipids, type 2 diabetes, meta-analysis

## Abstract

The aim of this systematic review and meta-analysis was to evaluate the effects of probiotics and glucose-lowering drugs (thiazolidinedione [TZD], glucagon-like pep-tide-1 receptor agonists [GLP-1 RA], dipeptidyl peptidase IV inhibitors, and sodium glucose co-transporter 2 inhibitors [SGLT-2i]) in patients with type 2 diabetes from randomized con-trolled trials (RCTs). The PubMed, Web of science, Embase, and Cochrane Library databases were searched on the treatment effects of probiotics and glucose-lowering drugs on glycemia, lipids, and blood pressure metabolism published between Jan 2015 and April 2021. We performed meta-analyses using the random-effects model. We included 25 RCTs (2,843 participants). Overall, GLP-1RA, SGLT-2i, and TZD significantly reduce fasting blood sugar (FBS) and glycated hemoglobin (HbA1c), whereas GLP-1 RA increased the risk of hypoglycaemia. Multispecies probiotics decrease FBS, total cholesterol (TC), and systolic and diastolic blood pressure (SBP, DBP). Moreover, subgroup analyses indicated that participants aged >55 years, BMI ≥30 kg/m^2^, longer duration of intervention, and subjects from Eastern countries, showed significantly higher reduction in FBS and HbA1c, TC, TG and SBP. This meta-analysis revealed that including multiple probiotic rather than glucose-lowering drugs might be more beneficial regarding T2D prevention who suffering from simultaneously hyperglycemia, hypercholesterolemia, and hypertension.

## Introduction

According to the data released by the International Diabetes Federation in 2017, 425 million people (8.8%) (age 20–79 years) have type 2 diabetes worldwide, whereas 114.4 million people (10.9%) in China have the disease (1). Type 2 diabetes most often accompanied with hyperglycaemia, hypertension, and abnormal blood lipid profiles (2), which frequently occur simultaneously and affect human health (3, 4). Therefore, it is important to determine effective methods for treating these comorbid diseases simultaneously.

Accumulating evidence indicates that there is a relationship between antihyperglycemic agents (e.g., thiazolidinedione [TZD] (5), glucagon-like peptide-1 receptor agonists [GLP-1 RA] (6–8), dipeptidyl peptidase IV inhibitors [DPP-4i] (9–11), sodium glucose co-transporter 2 inhibitors [SGLT-2i] (12)) and hyperglycaemia, hyperlipidaemia, and hypertension. Although these drugs improve glycaemic control in patients with type 2 diabetes, it has been indicated that different classes of antidiabetic drugs differ in glycaemic efficacy (13), and that different glucose-lowering agents can have varying impacts on a patient's lipid profile (14). Analyses of the effects of these anti-diabetic drugs on clinical outcomes have yielded conflicting results, and the differences across these classes of drugs have not been investigated. Additionally, despite having a comparable efficacy in glucose control, these drugs differ in their tolerability profiles: sulfonylureas induce hypoglycaemia; pioglitazone is associated with weight gain, fluid retention, and bone fractures; and acarbose is associated with gastrointestinal side effects (15). Hypoglycaemia, diarrhea, and urinary tract infections have also been associated with the use of glucose-lowering drugs (16, 17). Thus, avoidance of these adverse reactions is recommended as an important therapeutic consideration when selecting treatments and individualizing treatment goals. Moreover, whether a new drug is superior to another is interesting to clinicians, as well as patients.

The results of several studies have suggested a close association between probiotic administration and hyperglycaemia, lipid abnormalities, and hypertension (18–23). The findings of a previous study indicated that supplementation of multispecies probiotics can regulate glycaemic and lipid indicators (fasting blood sugar [FBS] and high-density lipoprotein cholesterol [HDL-C]); however, the authors noted no significant changes in other indices (24). Another study showed that there were significant reductions in FBS, insulin, and the Homeostatic Model Assessment of Insulin Resistance (HOMA-IR) index after intake of a single species probiotic (*lactobacillus casein*) (25). Therefore, we speculate that different patterns of consumption of probiotics may induce different effects. In addition, considering the adverse effects of glucose-lowering drugs, it is necessary to determine whether probiotics can be used instead of hypoglycaemic drugs to alleviate type 2 diabetes.

Previous studies of glucose-lowering drugs and probiotics are usually based on indirect comparisons, which are usually made using the same control, such as a placebo, by comparing probiotics and placebo, hypoglycemic drugs and placebo, to further compare the efficacy of probiotics and hypoglycemic drugs. To date, no study that involves head-to-head comparisons of the effects of GLP-1RA, DPP4i, SGLT2i, and TZD on glycemia, lipid profile, and blood pressure metabolism has been conducted. Previous meta-analyses only analyzed that the effects of glucose-lowering drugs or probiotic consumption on a few indicators of blood glucose, blood pressure and lipid profiles, sucn as only blood glucose, or blood pressure, or lipid. However, blood glucose indexes [FBS or glycated hemoglobin (HbA1c), insulin, and HMOA-IR), blood lipid indexes (total cholesterol (TC), triglycerides (TG), HDL-C, low-density lipoprotein cholesterol (LDL-C)], and blood pressure indexes [systolic blood pressure (SBP), and diastolic blood pressure (DBP)], were analyzed simultaneously in this study (26, 27). In addition, some of the parameters of the studies, including the classes of the glucose-lowering drugs and probiotics treatment patterns, and subject characteristics (patients' ages, BMI, country [influences diet and genetics], disease duration, and duration of intervention) varied; thus, the analyses yielded inconsistent results. To the best of our knowledge, there has been no comparative study of the efficacies of probiotics supplementation and glucose-lowering drugs for the treatment of type 2 diabetes.

Hence, the aim of this systematic review and meta-analysis was to evaluate and compare the effects of probiotics and glucose-lowering agents, including TZD, GLP-1RA, DPP4i, SGLT2i, on glycemia, lipid profile, and blood pressure in patients with type 2 diabetes. Furthermore, we conducted subgroup analyses to explore the associations between treatment effects and study characteristics, such as treatment patterns, patients' ages, BMI, country, and duration of intervention, to determine whether probiotics can be used instead of hypoglycaemic drugs for the treatment of type 2 diabetes.

## Methods

### Data sources and searches

The meta-analysis was conducted based on the Preferred Reporting Items for Systematic Review and Meta Analyses (PRISMA) guidelines (28). Three independent investigators (T.T.L, X.Q.X, and L.W) searched the PubMed, Embase, Web of Science, and Cochrane Library databases for relevant literatures published between Jan 2015 and April 2021. First of all, investigators conduct a preliminary screening based on the title and abstract of the literature, then the literature is screened again by reading the full text. If there were differences, they can decide whether to include them through discussion. If necessary, a fourth investigator (Q.P.W) can help solve them. The main keywords used were as follows: randomized controlled trials, type 2 diabetes, probiotic, glucose-lowering drugs, blood glucose, blood lipids, and blood pressure. The search strategy was conducted using Medical Subject Heading (MeSH) terms combined with keywords and Boolean operators (e.g., AND, OR, NOT). The details of the search strategy are outlined in Supplementary Table 1.

### Study selection

Eligible studies were selected according to the “participants, intervention, comparison, outcome, and study design” format (Supplementary Table 2).

The inclusion criteria were as follows: (1) studies that included adult participants with type 2 diabetes; (2) studies in which the interventions were probiotic supplementation or administration of glucose-lowering drugs; (3) studies that involved comparison of probiotic supplementation or glucose-lowering drug interventions with appropriate placebos; (4) studies that reported one or more of the following outcomes: FBS, HbA1c, insulin, HOMA-IR index, TC, TG, LDL-C, HDL-C, SBP, DBP, diarrhea, hypoglycaemia, or a combination of these; (5) randomized controlled trials (RCT); (6) studies published in English.

The exclusion criteria were as follows: (1) studies that included participants with gestational diabetes, prediabetes, or type 1 diabetes; (2) studies that did not involve probiotic supplementation or glucose-lowering drug interventions; (3) studies with no placebo control group; (4) studies in which the baseline outcomes or outcome changes were not reported, or studies with incomplete information on outcomes; (5) studies that were not RCTs; (6) animal studies; (7) reviews or meeting papers; (8) non-English studies.

### Data extraction and quality assessment

Data extraction was performed by two investigators (T.T.L and J.M) independently. Data extracted from each article included the following items: the name of the first author, publication year, sample size, country of study, participant characteristics (sex, age, weight, BMI), disease duration, study design, dose and kinds of placebo, use of probiotic supplementation or glucose-lowering drugs, duration of intervention, and outcome information (including the baseline and endpoint data or data regarding changes in FBS, HbA1c, insulin, HOMA-IR index, TC, TG, LDL-C, HDL-C, SBP, DBP, diarrhea, or hypoglycaemia).

The qualities of the included studies and their risks of bias were independently evaluated by two researchers using the Cochrane Handbook for Systematic Reviews of Interventions tool (29). Risk of bias was assessed in seven aspects, namely: random sequence generation (selection bias), allocation concealment (selection bias), blinding of participants and personnel (performance bias), incomplete outcome data (attrition bias), selective reporting (reporting bias), and other bias. These items could be scored as “low risk,” “high risk,” or “unclear.” The qualities and risks of bias of the included studies were analyzed using the Review Manager 5.3 software.

### Data synthesis and meta-analysis

Before statistical analyses, the measurement units of outcomes must be consistent in each study. FBS levels were recorded in mg/dL, which can be converted to mmol/L to reflect glucose concentrations and back to mg/dL when necessary. Insulin levels were collated in mIU/dL, which can be converted to pmol/L and back to mIU/dL when necessary. The lipid indices (TC, TG, HDL-C, and LDL-C) were collated in mg/dL, which can be converted mmol/L and back to mg/dL as appropriate.

### Statistical analysis

A meta-analysis was performed using the STATA software package, version 15.1 (StataCorp, College Station, TX) to analyse the effects of probiotic supplementation vs. those of glucose-lowering drugs. To estimate the effect size of each study, the means and standard deviations (SD) of the changes in outcomes from baseline to the endpoint were calculated and compared between treatment and placebo groups. The means and SDs of the changes were estimated according to the following formula (30):


(1) $$\begin{array}{r} \text{Mean changes}=\text{values at endpoint}-\text{values at baseline} \end{array}$$

(2) $$\begin{array}{r} SD=\sqrt{SD_{1}^{2}+SD_{2}^{2}-2*r*SD1*SD2}\left(r=0.5\right) \end{array}$$

Forest plots for probiotic and glucose-lowering drug groups were constructed using the STATA software package. A random effects model was used to evaluate the pooled effect of outcomes. The *I*^2^ statistic was used to represent the heterogeneity of the included studies, which ranged from 0 to 100%; proportions > 75% were considered to have high heterogeneity (31, 32). *P* < 0.05 indicated statistical significance (33).

Subgroup analyses were performed to investigate whether there were any significant differences between the patterns of consumption of probiotics and the classes of glucose-lowering drugs. Meta-regression analyses were also performed to determine whether participant characteristics, including age, BMI, country, and duration of intervention, were associated with the treatment effects (34). Qualitative analysis of publication bias was performed using visual funnel plots, whereas quantitative analysis was performed using Egger's tests (35). A sensitivity analysis was conducted to evaluate whether the included studies of each study could influence the overall results of the meta-analysis. The PRISMA checklist was used as a guide for checking the quality of our meta-analysis (Supplementary Table 3).

## Results

### Literature search results and study characteristics

**Figure 1:**
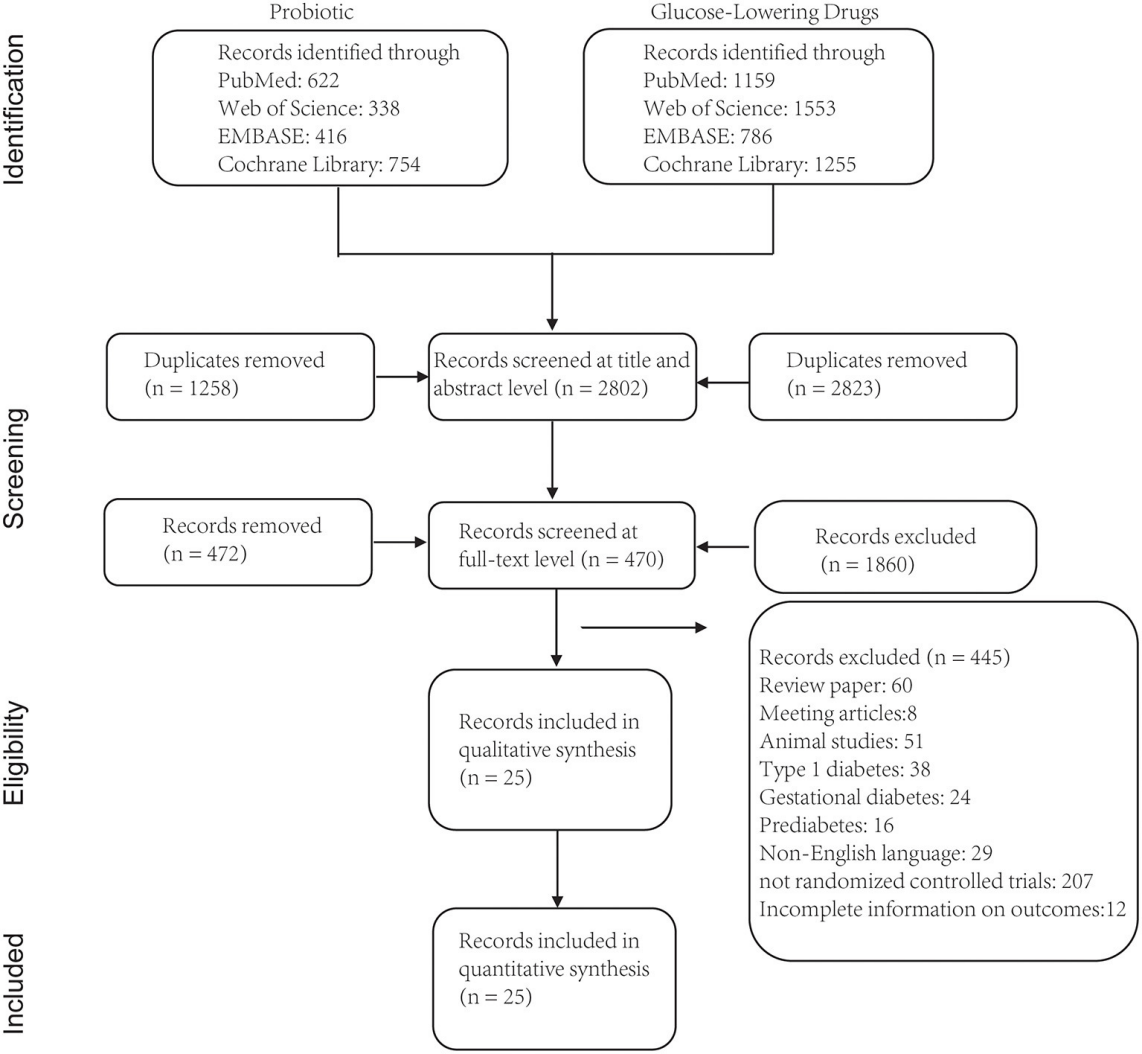
Flow chart depicting the literature search and selection strategy (based on PRISMA guideline).

Initially, 6,883 articles published between Jan 2015 and April 2021 were identified from the literature search. A total of 4,081 articles were excluded after reading their titles, and 470 articles were retrieved after reading their abstracts and full-text articles. Finally, 25 articles that satisfied the inclusion criteria were included in the meta-analysis (Figure 1).

The study and participant characteristics are summarized in Tables 1, 2. Of the 25 included studies, 14 were intervention studies (842 participants) that involved the administration of probiotics (single probiotics, multi-strain probiotics, and probiotics with co-supplements) (24, 25, 36–46, 50), whereas 11 were intervention trials (2001 participants) of glucose-lowering drugs (TZD, GLP-1 RA, SGLT-2i, and DPP-4i) (5–12, 47–49). The studies were conducted in Australia (*n* = 1), India (*n* = 2), Iran (*n* = 6), Ukraine (*n* = 1), Saudi Arabia (*n* = 1), Malaysia (*n* = 1), Brazil (*n* = 1), Sweden (*n* = 1), Denmark (*n* = 1), Turkey (*n* = 1), Netherlands (*n* = 2), Parkland (*n* = 1), Japan (*n* = 1), and China (*n* = 1); four other studies were carried out simultaneously in several countries. Regarding participants with type 2 diabetes, those included in seven of the studies were aged ≤ 55 years old, whereas those included in seven studies were aged > 55 years old. Eight studies included participants with a mean BMI ≥ 30 kg/m^2^, whereas 11 included those with mean BMI < 30 kg/m^2^. In addition, the disease duration of the participants ranged from 3 to 19 years. The duration of the probiotic interventions ranged from 6 to 12 weeks, whereas the duration of the glucose-lowering drug interventions ranged from 24 to 78 weeks.

**Table 1:** Characteristics of probiotic treatment studies included in this meta-analysis.

**References**	**Intervention/Control**	**Participants/Country**	**Subjects (Female/Male)**	**Age (year)**	**Weight (kg)**	**BMI (kg/m^2^)**	**Probiotic/Control**	**Dose**	**Diabetes duration (year)**	**Duration**	**Design**	**Measure outcomes**
Palacios et al. (36)	Probiotics	T2DM/ Australia	13	61.4 ± 8.9	100.1 ± 20.4	35.5 ± 6.2	L. plantarum, L. bulgaricus, L. gasseri, B. breve, B. animalis sbsp. lactis, B. bifidum, S. thermophilus, and S. boulardii	2 capsules	NA	12 wks	R, PC, DB	FBS/ HbA1c/ Insulin/ HOMA-IR
	Control		11	56.1 ± 12.3	101.7 ± 21.9	36.3 ± 7.5	Placebo	2 capsules	NA			
Madempudi et al. (37)	Probiotics	T2DM/ India	7/30	53.60	69.20	26.43	L. salivarius UBLS22, L. casei UBLC42, L. plantarum UBLP40, L. acidophilus UBLA34, B. breve UBBr01, and B. coagulans Unique IS2	3.0 ×10^10^ cfu	NA	12 wks	R, PC, DB	FBS/ HbA1c/ Insulin/ HOMA-IR/ TC/TG/HDL-C/LDL-C
	Control		9/28	50.50	68.00	25.97	Placebo	2 capsules	NA			
Khalili et al. (25)	Probiotics	T2DM/ Iran	13/7	43.95 ± 8.14	77.15 ± 13.58	29.50 ± 3.34	Lacidophilus. casei	10^8^ cfu capsules	4.00 ± 3.81	8 wks	R, PC, DB	FBS/HbA1c/Insulin/ HOMA-IR/ SBP/DBP/
	Control		13/7	45.00 ± 5.37	83.45 ± 15.84	31.94 ± 5.76	Placebo	capsules	3.67 ± 4.00			
Elham et al. (24)	Probiotics	T2DM/ Iran	13/17	58.60 ± 6.50	75.20 ± 15.60	27.70 ± 4.20	Lacidophilus+ L. casei + L. rhamnosus + L. bulgaricus + B. breve + B. longum + Streptococcus thermophilus	3.9 ×10^10^ cfu capsules	6.20 ± 3.10	6 wks	R, C, DB	FPG/ Insulin/ HOMA-IR /TC/TG/HDL-C/LDL-C
	Control		14/16	61.30 ± 5.20	74.10 ± 9.20	27.20 ± 4.20	Placebo	capsules	5.90 ± 2.90			
Kobyliak et al. (38)	Probiotics	T2DM/ Ukraine	NA	52.23 ± 1.74	99.32 ± 3.23	34.70 ± 1.29	Lactobacillus + Lactococcus + Bifidobacterium+ Propionibacterium+ Acetobacter	10 g/d	SC, DB, PC, P	8 wks	SC, DB, PC, P	FBS/ HbA1c/ Insulin
	Control		NA	57.18 ± 2.06	96.95 ± 4.35	35.65 ± 1.57	placebo					
Sabico et al. (39)	Probiotics	T2DM/ Saudi Arabia	20/19	48.00 ± 8.30	75.60 ± 11.00	29.40 ± 5.20	B.bifdum W23, B. lactis W52, L.acidophilus W37, L.brevis W63, L.casei W56, L.salivarius W24, L. lactis W19 +W58	5 ×10^9^ cfu/d sachets	SC, DB, R, PC	12 wks	SC, DB, R, PC	Glucose/ Insulin/ HOMA-IR/ TC/TG/ HDL-C/LDL-C/SBP/DBP
	Control		18/21	46.60 ± 5.90	79.50 ± 15.70	30.10 ± 5.00	placebo	sachets				
Feizollahzadeh et al. (40)	Probiotics	T2DM/ Iran	11/9	56.9 ± 1.81	70.84 ± 2.41	26.68 ± 0.71	soy milk containing L. planetarum A7	200 mL/d 2 ×10^7^ cfu	8.70 ± 2.10	8 wks	R, DB, PC	FBS/ TG/HDL-C/LDL-C/
	Control		10/10	53.60 ± 1.60	71.61 ± 2.55	26.58 ± 0.73	pure soy milk	200 mL/d	6.90 ± 4.90			
TajabadiEbrahimi et al. (37)	Probiotics	T2DM/ Iran	NA	64.20 ± 12.0	74.3 ± 13.7	32.30 ± 6.00	L.acidophilus, L. casei, B. bifdum	2 ×10^9^cfu	NA	12 wks	R, DB, PC	FPG/Insulin/HOMA-IR/ TC/TG/HDL-C/LDL-C
	Control		NA	64.00 ± 11.7	74.60 ± 15.1	29.60± 4.60	placebo	capsules	NA			
Firouzi et al. (41)	Probiotics	T2DM/ Malaysia	NA	52.90 ± 9.20	74.60 ± 15.1	29.20 ± 5.60	L.acidophilus, L. casei, L.lactis. Bifdobacterium, Actinobacteria, B. bifdum, B.longum and B.infantis.	10^10^ cfu 500 mL/d	NA	12 wks	R, DB, PC	FBG/ HbA1c/ insulin/ HOMA-IR/ TC/TG/HDL-C/LDL-C/SBP/DBP
	Control		NA	54.20 ± 8.30	76.60 ± 15.6	29.30 ± 5.30	placebo	500 mL/d	NA			
Tonucci et al. (42)	Probiotics	T2DM/ Brazil	11/12	51.83 ± 6.64	71.7 ± 12.43	27.49 ± 3.97	probiotic fermented milk (L.acidophilus La-5+B. animalis subsp lactis BB12)	120 g/d	6.00 ± 2.00	6 wks	DB, R, PC	FPG/HbA1c/ insulin/ HOMA-IR/ TC/TG/HDL-C/LDL-C
	Control		8/14	50.95 ± 7.20	77.15 ± 13.85	27.94 ± 4.15	conventional fermented milk	120 g/d	4.50 ± 2.00			
Alireza et al. (43)	Probiotics	T2DM/ Iran	12/18	NA	77.46 ± 13.26	28.89 ± 4.77	Fermented milk (kefir) contain L. casei, L.acidophilus and Bifidobacteria	600 mL/d	6.47 ± 0.90	8 wks	R, DB, PC	Glucose/HbA1C/ TC/TG/HDL-C/LDL-C
	Control	14/16	NA	74.92 ± 11.48	27.47 ± 3.55	Fermented milk(dough)	600 mL/d	7.36 ± 0.84			
Mobini et al. (44)	Probiotics	T2DM/ Sweden	3/11	64.00 ± 6.00	101.40 ± 18.00	32.30 ± 3.40	L. reuteri DSM 17938	1010cfu/d	14.4 ± 9.60	12 wks	R, DB, PC	FPG/HbA1c/ insulin/ TC/TG/HDL-C/LDL-C/SBP/DBP
	Control		4/11	65.00 ± 5.00	93.50 ± 12.10	30.70 ± 4.00	placebo	NA	18.3 ± 7.30			
Asemi et al. (45)	Probiotics	T2DM/ Iran	NA	NA	77.59 ± 13.65	30.15 ± 5.07	synbiotic food with Lactobacillus sporogenes	1 ×107 cfu	NA	6wks	R, DB, PC	FPG / insulin/ HOMA-IR/TC/TG/HDL-C/LDL-C/SBP/DBP
	Control		NA	NA	78.28 ± 13.42	30.15 ± 5.07	control food	9g	NA			
Hove et al. (46)	Probiotics	T2DM/ Denmark	NA	58.50 ± 7.70	93.20 ± 17.90	29.20 ± 3.80	Milk fermented with L. helveticus	300 mL	NA	12 wks	R, DB, P, PC	Glucose/HbA1c/Insulin/HOMA-IR/ TC/TG/HDL-C/LDL-C
	Control		NA	60.60 ± 5.20	85.20 ± 9.50	27.70 ± 3.30	Artificially acidified milk	300 mL	NA			

**Table 2:** Characteristics of hypoglycemic drug treatment studies included in this meta-analysis.

**References**	**Intervention/Control**	**Participants/Country**	**Subjects (Female/Male)**	**Age (year)**	**Weight (kg)**	**BMI (kg/m^2^)**	**Glucose-lowering drugs/Control**	**Diabetes duration (year)**	**Duration**	**Design**	**Measure outcomes**
Rastogi et al. (5)	Thiazolidinediones	T2DM/India	15	53.1 (8.8)	69.9 (12.6)	27.3 (2.7)	Saroglitazar	3.2 (1.5)	12 wks	R.P.C.DB	FPG/HbA1c/ TC/HDL-C/LDL-C
	Control		15	54.9 (7.8)	78.0 (11.7)	28.9 (2.8)	Placebo	3.3 (1.8)			
Bulut et al. (47)	DPP-4I	T2DM/Turkey	35	74.4 ± 7.9	65.0 ± 10.1	28.5 ± 4.2	Vildagliptin (+)	11.3 ± 7.6	24 wks	R	TG/HDL-C/LDL-C
	Control		43	79.7 ± 4.8	74.2 ± 12.4	29.3 ± 5.1	Placebo	18.4 ± 9.9			
Bernard et al. (6)	GLP-1 RA	T2DM/Austria, Canada, Japan, Norway, Russia, and the USA	151	57.5 (8.9)	89.6 (19.5)	31.1 (6.2)	Semaglutide 1.0 mg	NA	30 wks	R.DB.PC	FPG/TC/HDL-C/LDL-C/ SBP/DBP
	Control		151	56.6 (10.1)	93.8 (22.3)	32.7 (6.9)	Placebo	NA			
Eyk et al. (7)	GLP-1 RA	T2DM/Netherlands	22	55 ± 11	81.9 ± 11.0	30.4 ± 3.8	Liraglutide	19 ± 10	26 wks	R.DB.PC	HbA1c/TC/TG/HDL-C/LDL-C
	Control		25	55 ± 9	77.8 ± 12.4	28.6 ± 4.0	Placebo	17 ± 10			
Guja et al. (8)	GLP-1 RA	T2DM/Hungary, Poland, Romania, Slovakia, South Africa and the USA	231	57.8 ± 9.0	93.3 ± 20.0	33.3 ± 6.1	Exenatide QW	11.5 ± 6.6	28 wks	R.DB.C	FPG/HbA1c/ TC/TG/HDL-C/LDL-C/ SBP/DBP
	Control		230	57.6 ± 10.3	94.7 ± 19.8	34.1 ± 6.6	Placebo	11.1 ± 6.1			
Boer et al. (9)	DPP-4I	T2DM/Netherlands	13/9	63	97.9 ± 17.6	32.3 (27.8–38.2)	linagliptin	1.5 (0–5)	26 wks	R.DB.C	FPG/HbA1c/TG/HDL-C/LDL-C
	Control		14/8	62	95.3 ± 13.2	29.0 (27.4–34.2)	Placebo	1.0 (0–3.3)			
Guzman et al. (10)	GLP-1 RA	T2DM/USA,Canada,France	65	56.9 (8.3)	94.2 (22.5)	32.6 (5.5)	LY2409021	12.4 (6.3)	24 wks	R.DB.C	HbA1c/ SBP/DBP
	DPP-4I		41	57.1 (9.0)	94.0 (20.9)	31.8 (6.1)	Sitagliptin	10.9 (6.5)			
	Control		68	57.8 (8.2)	85.7 (17.9)	31.2 (4.9)	Placebo	10.2 (6.3)			
Vanderheiden et al. (48)	GLP-1 RA	T2DM/Parkland	35	52.8 (8.1)	114.6 (21.4)	40.7 (6.7)	Liraglutide	16 (12–23)	24 wks	R.DB.PC	FPG/HbA1c/TC/HDL-C/LDL-C/SBP/DBP
	Control		36	55.5 (6.6)	116.1 (26.6)	41.6 (10.4)	Placebo	18 (13–27)			
Inagaki et al. (49)	DPP-4I	T2DM/ Japan	101	58 (52–65)	NA	25.4 (4.42)	Trelagliptin	6.3 (5.93)	24 wks	R.DB.C.P	FPG/HbA1c
	DPP-4I		92	60 (53–65)	NA	24.7 (3.79)	Alogliptin	7.1(5.93)			
	Control		50	62 (54–67)	NA	24.6 (4.27)	Placebo	7.54(5.50)			
Rosenstock et al. (15)	SGLT-2I	T2DM/ Denmark, France, Ireland, Korea, Portugal, UK and USA	170	NA	90.5 ± 1.7	NA	Empagliflozin 10 mg	NA	78 wks	R.DB.C	FPG/HbA1c
	SGLT-2I		169	NA	91.6 ± 1.5	NA	Empagliflozin 25 mg	NA			
	Control		155	NA	94.7 ± 1.7	NA	Placebo	NA			
Wu et al. (15)	DPP-4I	T2DM/ China	34	52.5 ± 11.0	67.05 ± 8.12	24.37 ± 2.09	Linagliptin	NA	24 wks	R.P.C.DB	FPG /HbA1c / HOMA-IR/TC/TG/HDL-C/LDL-C/SBP/DBP
	Control		23	51.2 ± 7.5	65.24 ± 8.45	24.11 ± 2.28	Placebo	NA			
	Control		23	51.2 ± 7.5	65.24 ± 8.45	24.11 ± 2.28	Placebo	NA			

*Data are presented as means ± SDs or as a range. T2DM, type 2 diabetes mellitus; FBS, fasting blood sugar; HbA1c, glycated hemoglobin; HOMA-IR, homeostasis model assessment of insulin resistance; TC, total cholesterol; TG, triglycerides; HDL-C, high-density lipoprotein cholesterol; LDL-C, low-density lipoprotein cholesterol; SBP, systolic blood pressure, DBP, diastolic blood pressure; DPP-4i, Dipeptidyl Peptidase-4 Inhibitors; GLP-1 RA, Glucagon-like peptide 1 receptor agonist; Sodium Glucose Co-Transporter 2 Inhibitor, SGLT-2I; P, parallel; R, randomized; PC, placebo-controlled study; DB, double-blinded*.

### Quality assessment of the studies

**Figure 2:**
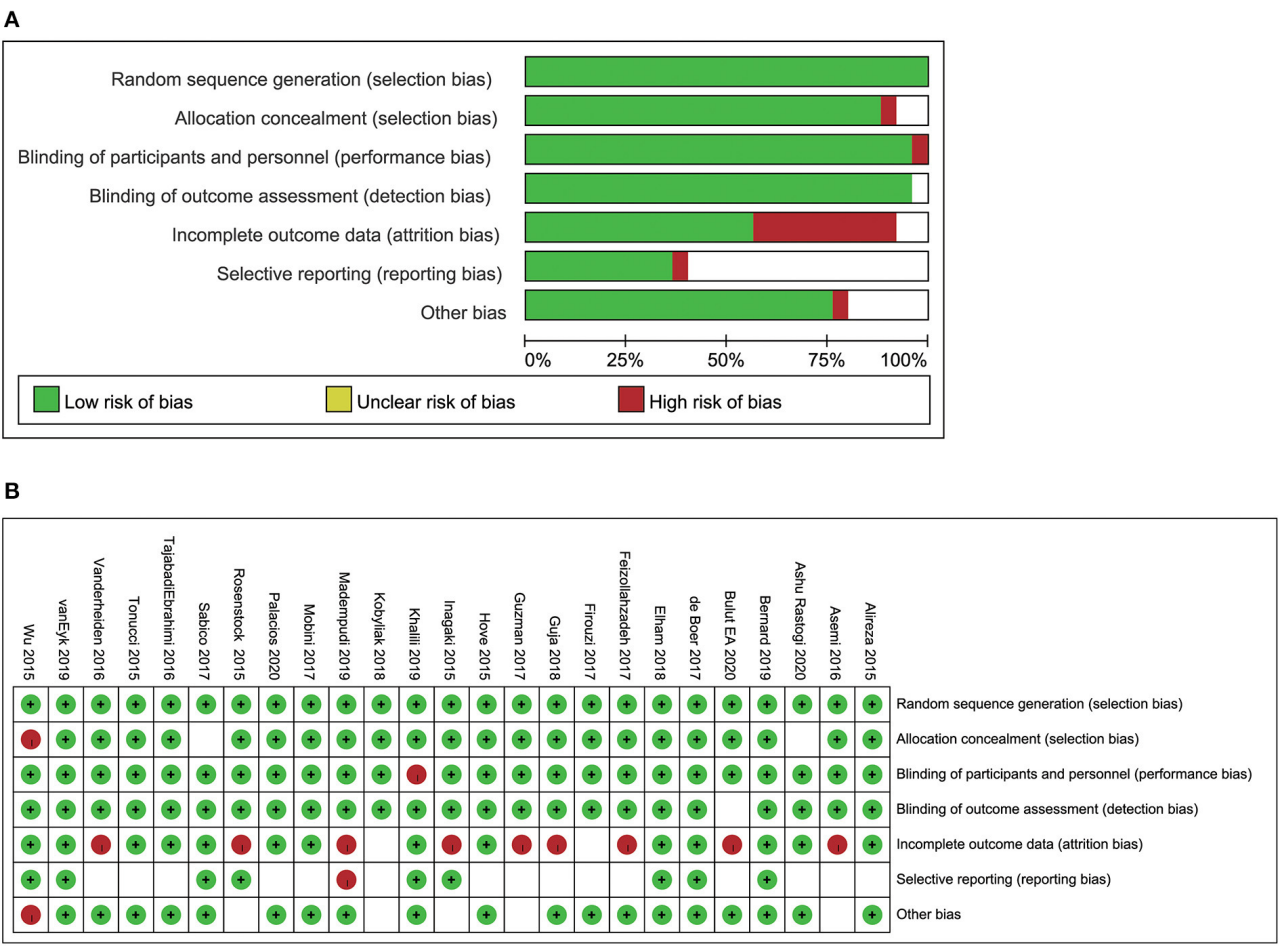
Risk of bias analysis **(A)** the analysis of the individual studies included in the systermatic review and meta-analysis. **(B)** The summary of the risk of bias analysis.

Most of the included studies had a low risk of random sequence generation. Allocation concealment was not clearly mentioned in two of the articles. One study had a high risk of blinding of participants and personnel, whereas blinding of outcome assessment was unclear in one study. Nine studies had a high risk of incomplete outcome data, whereas selective reporting and other risks were unclear in several studies. The overall quality assessment of the included studies is shown in Figure 2.

### The effects of probiotics and glucose-lowering drugs on glucose metabolism

#### Effects of probiotics and glucose-lowering drugs on fasting blood sugar

**Figure 3:**
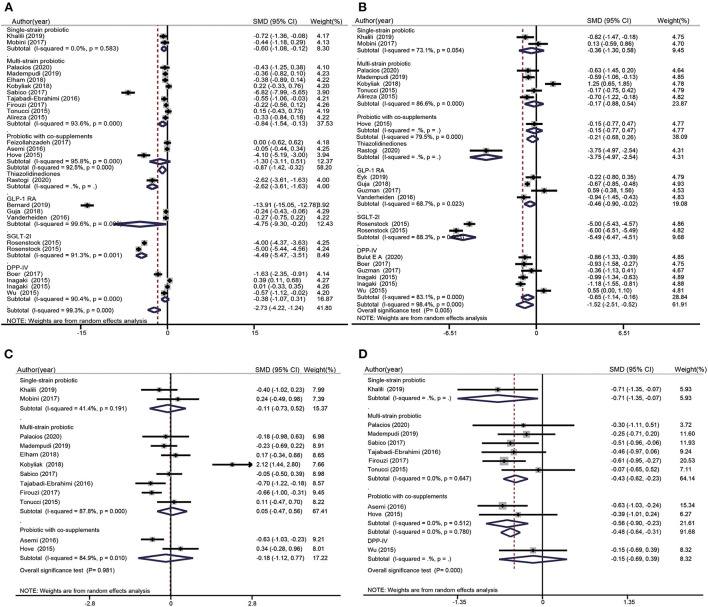
Forest plots for the effect of probiotics supplementation and glucose-lowering drugs on FBS (mg/dL) **(A)**, HbA1c (%) **(B)**, Insulin (mU/mL) **(C)**, and HOMA-IR **(D)**, compared to placebo in pooled analysis. For each study, the solid black diamonds represent the point estimate of the intervention effect. The horizontal line joins the lower and upper limits of the 95% CI of this effect. The open diamonds represent the subgroup and overall SMD determined with a random-effects model.

FBS data were included 13 probiotics intervention studies and eight glucose-lowering drug intervention studies. Overall, the probiotic groups showed significant reduction in FBS compared with the placebo groups (standardized mean difference (SMD): −0.87 mg/dL; 95% CI: −1.42, −0.32 mg/dL; *I*^2^ = 92.5%, p=0.000). However, the glucose-lowering drug groups showed more significant decrease in FBS compared with the placebo groups (SMD: −2.73 mg/dL; 95% CI: −4.22, −1.24 mg/dL; *I*^2^ = 99.3%, *p* = 0.000) (Figure 3A). The test for subgroup differences showed that single species probiotics and multispecies probiotics significantly lowered FBS. In addition, we observed a significant reduction in FBS in groups treated with TZD, GLP-1 RA, and SGLT-2i; however, there was no significant difference between the DPP-4i and placebo groups.

#### Effects of probiotics and glucose-lowering drugs on glycated hemoglobin level

HbA1c data were included in seven probiotics intervention studies and in 11 glucose-lowering drug intervention studies (Figure 3B). The overall effect of probiotic supplementation on HbA1c was not significant (SMD: −0.21%; 95% CI: −0.68%, 0.26%; *I*^2^ = 79.5%, *p* = 0.000). There was a significant decrease in HbA1c levels in the glucose-lowering drug groups compared with the control groups (SMD: −1.52%; 95% CI: −2.51%, −0.52%, *I*^2^= 98.4%, *p* = 0.000). The test for subgroup differences showed that supplementation with single species probiotics, multispecies probiotics, and probiotics with co-supplements had no significant effects on HbA1c, whereas participants treated with TZD, GLP-1 RA, SGLT-2i, and DPP-4i showed significant reduction in HbA1c levels compared with the placebo groups. This reduction was particularly notable in the comparison of SGLT-2i groups and placebo groups (−5.49%; 95% CI: −6.47%, −4.51%; *I*^2^ = 88.3%, *p* = 0.003).

#### Effects of probiotics on insulin

Insulin data were included in 10 probiotics intervention studies and none of the glucose-lowering drug intervention studies. However, the data in the 10 studies only referred to the effect of probiotic supplementation on insulin (Figure 3C). Overall, the difference between the probiotic and placebo groups were not significant (SMD: −0.02 mU/L; 95% CI: −0.39, 0.36 mU/L; *I*^2^ = 83.6%, *p* = 0.000). The test for subgroup differences showed that there was no significant reduction in insulin after supplementation with single species probiotics, multispecies probiotics, and probiotics with co-supplements.

#### Effects of probiotics and glucose-lowering drugs on the homeostatic model assessment of insulin resistance index

HOMA-IR data were pooled from seven probiotics intervention studies and one glucose-lowering drug intervention study (Figure 3D). Overall, probiotic supplementation significantly decreased the HOMA-IR index compared with the placebo (SMD: −0.48; 95% CI: −0.64, −0.31; *I*^2^= 0.0%; *p* = 0.780), regardless of whether the probiotic used was single species, multispecies, or used with a co-supplement. Nevertheless, participants treated with glucose-lowering drugs (DPP-4i) showed no significant difference in the HOMA-IR index compared with control groups (SMD: −0.15; 95% CI: −0.69, 0.39).

### Effects of probiotics and glucose-lowering drugs on lipid metabolism

#### Effects of probiotics and glucose-lowering drugs on total cholesterol levels

**Figure 4:**
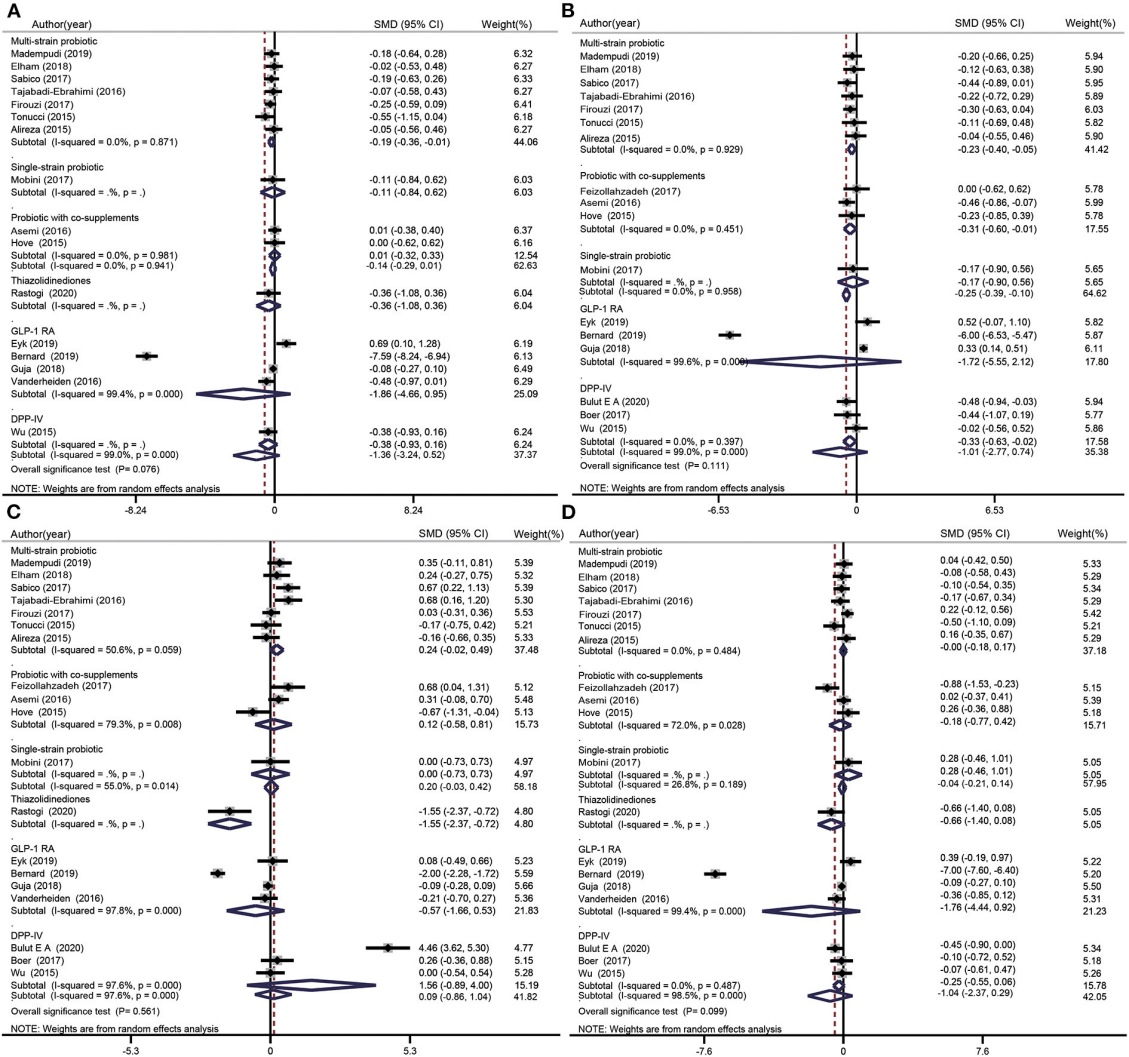
Forest plots for the effect of probiotics supplementation and glucose-lowering drugs on TC (mg/dL) **(A)**, TG (mg/dL) **(B)**, HDL-C (mg/dL) **(C)**, and LDL-C(mg/dL) **(D)** compared to placebo in pooled analysis. For each study, the solid black diamonds represent the point estimate of the intervention effect. The horizontal line joins the lower and upper limits of the 95% CI of this effect. The open diamonds represent the subgroup and overall SMD determined with a random-effects model.

TC data were included in nine probiotics intervention studies and in six glucose-lowering drug intervention studies (Figure 4A). Overall, there was no significant difference between the TC levels of participants treated with probiotics (SMD: −0.14 mg/dL; 95% CI: −0.29, 0.01 mg/dL; *I*^2^ = 0.0%; *p* = 0.941) or glucose-lowering drugs (SMD: −1.36 mg/dL; 95% CI: −3.24, 0.52 mg/dL; *I*^2^ = 99.0%; *p* = 0.000) and those of the placebo groups. The test for subgroup differences showed that multispecies probiotics significantly reduced TC levels (SMD: −0.19 mg/dL; 95% CI: −0.36, −0.01 mg/dL; *I*^2^= 0.00%; *p* = 0.871), whereas other treatment methods (single species probiotics, probiotics with co-supplements, TZD, GLP-1 RA, and DPP-4i) did not significantly decrease TC levels.

#### Effects of probiotics and glucose-lowering drugs on triglyceride levels

TG data were included in 11 probiotics intervention studies and six glucose-lowering drug intervention studies (Figure 4B). Overall, the difference between the TG levels of the probiotics and placebo groups was −0.25 mg/dL (95% CI: −0.39, −0.10 mg/dL; *I*^2^ = 0.0%, *p* = 0.958). However, there was no significant difference in TG level after treatment with glucose-lowering drugs (SMD: −1.01 mg/dL; 95% CI: −2.77, 0.74 mg/dL, *I*^2^ = 99.0%, *p* = 0.000). The test for subgroup differences showed a significant reduction in TG level when multispecies probiotics, probiotics with co-supplements, or DPP-4i were used, whereas single species probiotics and GLP-1 RA had no effect on TG levels.

#### Effects of probiotics and glucose-lowering drugs on high-density lipoprotein cholesterol levels

HDL-C data were included in 11 probiotic intervention studies and in eight glucose-lowering drug intervention studies (Figure 4C). Overall, there was no significant increase in HDL-C after use of probiotics (SMD: 0.20 mg/dL; 95% CI: −0.03, 0.42 mg/dL; *I*^2^ = 55.0%, *p* = 0.014) or glucose-lowering drugs (SMD: 0.09 mg/dL; 95% CI: −0.86, 1.04 mg/dL; *I*^2^ = 97.6%, *p* = 0.000). The test for subgroup differences showed a significant decrease in HDL-C when TZDs were used (SMD: −1.55 mg/dL; 95% CI: −2.37, −0.72 mg/dL), whereas other treatment methods (single species probiotics, multispecies probiotics, probiotics with co-supplements, GLP-1 RA, and DPP-4i) induced no significant difference in HDL-C levels compared with the placebo.

#### Effect of probiotics and glucose-lowering drugs on low-density lipoprotein cholesterol levels

LDL-C data were included in 11 probiotic intervention studies and in eight glucose-lowering drug intervention studies (Figure 4D). Overall, no significant changes in LDL-C were observed when probiotics (SMD: −0.04 mg/dL; 95% CI: −0.21, 0.14 mg/dL; *I*^2^ = 26.8%, *p* = 0.189) or glucose-lowering drugs were used (SMD: −1.04 mg/dL; 95% CI: −2.37, 0.29 mg/dL; *I*^2^ = 98.5%; *p* = 0.000). The test for subgroup differences showed no significant difference between the treatment groups (single species probiotics, multiple species probiotics, probiotics with co-supplements, TZD, GLP-1 RA, and DPP-4i) and control groups.

### Effects of probiotics and glucose-lowering drugs on blood pressure metabolism

#### Effects of probiotics and glucose-lowering drugs on systolic blood pressure

**Figure 5:**
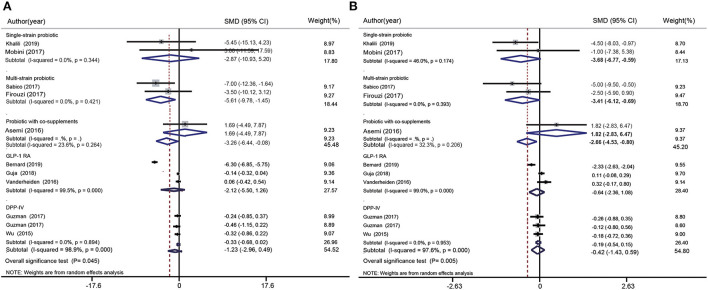
Forest plot for the effect of probiotics supplementation and glucose-lowering drugs on SBP (mmHg) **(A)** and DBP (mmHg) **(B)** compared to placebo. For each study, the solid black diamonds represent the point estimate of the intervention effect. The horizontal line joins the lower and upper limits of the 95% CI of this effect. The open diamonds represent the subgroup and overall SMD determined with a random-effects model.

SBP data were included in five probiotic intervention studies and in five glucose-lowering drug intervention studies (Figure 5A). Overall, the SBP of the probiotics group was significantly lower than that of the control group (SMD: −3.26 mmHg; 95% CI: −6.44, −0.08 mmHg; *I*^2^ = 23.6%, *p* = 0.044). However, glucose-lowering drugs did not induce any significant change in SBP (SMD: −1.23 mmHg; 95% CI: −2.96, 0.49 mmHg; *I*^2^ = 98.9%, *p* = 0.000). The test for subgroup differences showed that multispecies probiotics significantly decreased SBP (SMD: −5.61 mmHg; 95% CI: −9.78, −1.45 mmHg; *I*^2^ = 0.00%, *p* = 0.421). Nevertheless, there were no significant differences between the other treatment groups (single species probiotics, probiotics with co-supplements, GLP-1 RA, and DPP-4i) and the control groups.

#### Effects of probiotics and glucose-lowering drugs on diastolic blood pressure

DBP data was included in five probiotic intervention studies and in five glucose-lowering drug intervention studies (Figure 5B). Overall, probiotics significantly reduced DBP (SMD: −2.66 mmHg; 95% CI: −4.53, −0.80 mmHg; *I*^2^ = 32.3%, *p* = 0.206). However, no significant changes were observed when glucose-lowering drugs were used (GLP-1 RA and DPP-4i). The test for subgroup differences showed that single species probiotics (SMD: −3.68 mmHg; 95% CI: −6.77, −0.59 mmHg; *I*^2^ = 46.0%, *p* = 0.174) and multispecies probiotics (SMD: −3.41 mmHg; 95% CI: −6.12, −0.69 mmHg; *I*^2^ = 0.0%, *p* = 0.393) significantly reduced DBP.

### Adverse effects of glucose-lowering drugs on diarrhea and hypoglycaemia

#### Effect of glucose-lowering drugs on diarrhea

The effect of glucose-lowering drugs on diarrhea was reported in one article (Supplementary Figure 1A). It indicated that there was no significant difference between the GLP-1 RA group (SMD: 1.81; 95% CI: 0.83, 3.94) and the control group in terms of diarrhea.

#### Effect of glucose-lowering drugs on hypoglycaemia

The effect of glucose-lowering drugs on hypoglycaemia was reported in two articles; one was for a GLP-1 RA intervention study and the other was for a DPP-4i intervention study (Supplementary Figure 1B). Overall, glucose-lowering drugs significantly increased hypoglycaemia (SMD: 4.70; 95% CI: 1.54, 14.36; *I*^2^ = 0.0%; *p* = 0.571). The test for subgroup differences also showed that GLP-1 RA significantly increased hypoglycaemia (SMD: 5.23; 95% CI: 1.56, 17.48). However, there was no significant difference hypoglycaemia after DPP-4i was used (SMD: 1.97; 95% CI: 0.08, 46.33).

### Subgroup analyses of the effects of probiotics and glucose-lowering drugs on glucose metabolism

The subgroup analyses of the studies included in the meta-analysis was stratified according to the four glycaemic indices and based on four specific factors, namely: age, BMI, country, and duration of intervention (Supplementary Figures 2–5). Notably, the subgroup analyses showed that the effects of probiotic supplementation and glucose-lowering drugs on FBS and HbA1c were more significantly decreased among participants aged > 55 years old or with BMI ≥ 30 kg/m^2^ at baseline (*P* = 0.000, *P* = 0.002, and *P* = 0.000, respectively). Moreover, we observed that participants of Eastern descent had more decreased HbA1c levels than their Western counterparts (*P* = 0.005). The effects of probiotics and glucose-lowering drugs on the HOMA-IR index were more significantly reduced among participants aged ≤ 55 years old, with BMI ≥ 30 kg/m^2^ at baseline, or of Eastern descent (*P* = 0.000, *P* = 0.005, and *P* = 0.000). Additionally, we noted that the duration of probiotic interventions was generally <12 weeks, whereas the duration of glucose-lowering drug interventions was more than 12 weeks.

### Subgroup analyses of effects of probiotics and glucose-lowering drugs on lipid metabolism

The subgroup analyses showed that the effects of probiotic supplementation and glucose-lowering drugs on TC and TG levels were more significantly decreased among participants of Eastern descent (*P* = 0.041 and *P* = 0.001, respectively). Moreover, we found that participants with BMI < 30 kg/m^2^ at baseline had more decreased TG levels than those with BMI > 30 kg/m^2^ (*P* = 0.005). However, there was no significant difference between the HDL-C level, LDL-C level, and age, BMI, country, and duration of intervention of the participants in the probiotics and glucose-lowering drugs groups (Supplementary Figures 6–9).

### Subgroup analyses of the effects of probiotics and glucose-lowering drugs on blood pressure metabolism

The effects of probiotic and glucose-lowering drugs on SBP were more decreased in participants of Eastern descents than in those of Western descent (*p* = 0.030). However, there was no significant difference between the probiotics and glucose-lowering drugs groups in terms of DBP indicators, age, BMI, geographic area, and duration of intervention (Supplementary Figures 10, 11).

### Publication bias

Funnel plots (Supplementary Figures 12, 14, 16) and Egger's test (quantitative) (Supplementary Figures 13, 15, 17) were used to assess the publication biases of the included studies. There was a significant difference between the publication biases for insulin in probiotic intervention studies and glucose-lowering drug intervention studies (*P* = 0.039). However, there were no significant differences in the publication biases for FBS (*P* = 0.075), HbA1c (*P* = 0.991), and the HOMA-IR index (*P* = 0.160) in the two types of studies. The probiotics and glucose-lowering drug intervention studies for the control of blood lipids and blood pressure showed no significant publication biases for TC, TG, HDL-C, LDL-C, SDP, and DBP (*P* = 0.325, *P* = 0.227, *P* = 0.136, *P* = 0.348, *P* = 0.414 and *P* = 0.919, respectively).

### Meta-regression and sensitivity analysis

Meta-regression analysis was performed to assess the sources of heterogeneity in the studies. Univariate meta-regression analysis showed that participants' age, BMI at baseline, countries, and duration of intervention were not associated with change in FBS, HbA1c, insulin, HOMA-IR index, TC, TG, HDL-C, LDL-C, SBP, and DBP levels (*p* ≥ 0.05) (Table 3). Furthermore, sensitivity analysis was performed to detect the publication bias for insulin. We observed that no study may influence the pooled results or total effect size. Meanwhile, the results of the sensitivity analysis of FBS, HbA1c, the HOMA-IR index, TC, TG, HDL-C, LDL-C, SBP, and DBP also suggested that no study may affect the pooled results or total effect size (Supplementary Figures 18–20).

**Table 3:** Results of meta-regression analyses with age, BMI, country, and duration of intervention in all indexes (FBS, HbA1c, Insulin, HOMA-IR, TC, TG, HDL-C, LDL-C, SBP, and DBP) as independent variables.

	***n***	**[95% CI]**	**Coef**.	**Std. Err**.	**R2**	***p*** **value**
FBS					
Age					
Years ≤ 55	7	[−1.829, 0.420]	0.460	1.121	−12.08%	0.686
Years > 55	8					
Baseline BMI					
BMI ≥ 30	10	[−0.956, 3.452]	1.248	1.049	−12.08%	0.250
BMI <30	6					
Country					
Eastern	12	[−4.096, 2.125]	−0.986	1.480	−12.08%	0.514
Western	6					
Duration of intervention					
≤ 8 wks	7	[−1.803, 2.041]	0.119	0.915	−12.08%	0.898
8 < wks ≤ 12	6					
12 < wks ≤ 24	5					
>24 wks	4					
HbA1c					
Age					
Years ≤ 55	6	[−1.830, 0.421]	−0.704	0.528	23.79%	0.202
Years > 55	8					
Baseline BMI					
BMI ≥ 30	6	[−1.531, 0.719]	−0.406	0.528	23.79%	0.454
BMI <30	8					
Country					
Eastern	7	[−1.511, 1.579]	0.339	0.725	23.79%	0.963
Western	9					
Duration of intervention weeks					
≤ 8 wks	4	[−1.570, 0.250]	−0.660	0.427	23.79%	0.143
8 < wks ≤ 12	3					
12 < wks ≤ 24	7					
>24 wks	4					
Insulin					
Age					
Years ≤ 55	5	[−1.096, 0.781]	−0.157	0.383	−44.68%	0.696
Years > 55	4					
Baseline BMI	
BMI ≥ 30	3	[−0.485, 1.257]	0.386	0.356	−44.68%	0.320
BMI <30	5					
Country					
Eastern	7	[−1.301, 1.729]	0.214	0.619	−44.68%	0.741
Western	4					
Duration of intervention					
≤ 8 wks	6	[−1.513, 1.266]	−0.124	0.568	−44.68%	0.834
8 < wks ≤ 12	5					
12 < wks ≤ 24	-					
>24 wks	-					
HMOA-IR					
Age					
Years ≤ 55	6	[−0.376, 0.261]	−0.057	0.115	-	0.643
Years > 55	2					
Baseline BMI					
BMI ≥ 30	1	[−0.341, 0.350]	0.004	0.124	-	0.973
BMI <30	5					
Country					
Eastern	8	[−0.435,1.055]	0.310	0.268	-	0.313
Western	2					
Duration of intervention					
≤ 8 wks	4	[−0.344, 0.593]	0.125	0.169	-	0.501
8 < wks ≤ 12	4					
12 < wks ≤ 24	1					
>24 wks	-					
TC					
Age					
Years ≤ 55	6	[−1.833, 1.238]	−0.297	0.698	3.37%	0.678
Years > 55	5					
Baseline BMI					
BMI ≥ 30	5	[−1.154, 2.287]	0.566	0.782	3.37%	0.484
BMI <30	9					
Country					
Eastern	9	[−2.940, 0.853]	−1.043	0.862	3.37%	0.251
Western	5					
Duration of intervention					
≤ 8 wks	6	[−1.867, 0.948]	−0.460	0.639	3.37%	0.587
8 < wks ≤ 12	4					
12 < wks ≤ 24	3					
>24 wks	3					
TG					
Age					
Years ≤ 55	6	[−1.508, 0.908]	−0.299	0.554	−6.67%	0.599
Years > 55	7					
Baseline BMI					
BMI ≥ 30	5	[−0.929, 1.460]	0.265	0.548	−6.67%	0.638
BMI <30	9					
Country					
Eastern	8	[−2.217, 0.957]	−0.630	0.728	−6.67%	0.404
Western	7					
Duration of intervention					
≤ 8 wks	6	[−1.376, 0.804]	−0.286	0.500	−6.67%	0.578
8 < wks ≤ 12	5					
12 < wks ≤ 24	2					
>24 wks	4					
HDL-C					
Age					
Years ≤ 55	6	[−0.847, 1.312]	0.232	0.503	−28.26%	0.651
Years > 55	8					
Baseline BMI					
BMI ≥ 30	6	[−1.172, 1.041]	−0.066	0.516	−28.26%	0.900
BMI <30	10					
Country					
Eastern	9	[−1.528, 1.117]	−0.206	0.616	−28.26%	0.744
Western	8					
Duration of intervention					
≤ 8 wks	6	[−0.907, 0.961]	0.027	0.436	−28.26%	0.952
8 < wks ≤ 12	5					
12 < wks ≤ 24	4					
>24 wks	4					
LDL-C					
Age					
Years ≤ 55	6	[−1.368, 1.068]	−0.150	0.568	7.26%	0.796
Years > 55	8					
Baseline BMI					
BMI ≥ 30	6	[0.920, 1.568]	0.324	0.580	7.26%	0.585
BMI <30	10					
Country					
Eastern	9	[−2.546, 0.442]	−1.051	0.697	7.26%	0.153
Western	8					
Duration of intervention					
≤ 8 wks	6	[−1.318, 0.786]	−0.266	0.490	7.26%	0.596
8 < wks ≤ 12	5					
12 < wks ≤ 24	4					
>24 wks	4					
SBP					
Age					
Years ≤ 55	3	[−1.775, 2.962]	0.593	0.968	−8.81%	0.563
Years > 55	4					
Baseline BMI					
BMI ≥ 30	5	[−2.306, 3.352]	0.523	1.156	−8.81%	0.667
BMI <30	4					
Country					
Eastern	6	[−7.621, 2.777]	−2.421	2.124	−8.81%	0.298
Western	2					
Duration of intervention					
≤ 8 wks	4	[−2.539, 3.973]	0.717	1.330	−8.81%	0.609
8 < wks ≤ 12	1					
12 < wks ≤ 24	2					
>24 wks	4					
DBP					
Age					
Years ≤ 55	3	[−0.551, 1.477]	0.463	0.414	−18.41%	0.306
Years > 55	4					
Baseline BMI					
BMI ≥ 30	5	[−1.057, 1.400]	0.172	0.502	−18.41%	0.744
BMI <30	4					
Country					
Eastern	6	[−3.322, 1.131]	−1.095	0.910	−18.41%	0.274
Western	2					
Duration of intervention					
≤ 8 wks	4	[−0.893, 1.931]	0.519	0.577	−18.41%	0.403
8 < wks ≤ 12	1					
12 < wks ≤ 24	3					
>24 wks	2					

*CI, confidence interval; Coef, coefficient; Std. Err, standard error; R^2^, R Square*.

## Discussion

In this systematic review and meta-analysis, we compared the effects of probiotic supplementation and glucose-lowering drugs on the blood glucose, lipids, and blood pressure of patients with type 2 diabetes. We found that except for DPP-4i, glucose-lowering drugs caused a significantly greater reduction in FBS than probiotics. The results also indicated that glucose-lowering drugs reduced HbA1c level more than probiotics; SGLT-2i in particular induced the greatest decrease in HbA1c. We noted that probiotic supplementation reduced the HOMA-IR index, and that multispecies probiotics were associated with reduction in TC and TG levels; however, DPP-4i only decreased TG levels. TZD was associated with decrease in HDL-C, whereas probiotic supplementation was associated with higher decrease in SBP and DBP. The results indicated that GLP-1 RA increases the risk of hypoglycaemia.

**Figure 6:**
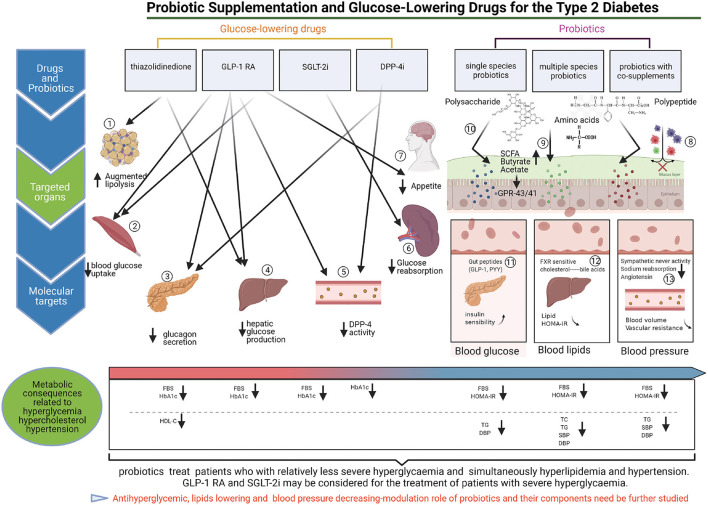
The targeted regulation mechanisms of probiotics and glucose-lowering drugs on antidiabetic. (1) TZDs can inhibit lipolysis in adipose tissue, and decrease hyperglycemia. (2) TZDs and GLP1-RAs can promote blood glucose uptake by the skeletal muscle contributes to reduce blood glucose. (3) GLP1-RA can promote the secretion of insulin from pancreas, meanwhile, GLP1-RA and DPP-4i can inhibit glucagon secretion by pancreas, and contribute to reduce the blood glucose. (4) TZDs, and GLP1-RA can inhibit endogenous hepatic glucose production, and reduce hyperglycemia. (5) GLP-1RA can inhibit DPP-4 enzymatic activity in circulation participates in decrease blood hyperglycemia. (6) SGLT2i (Sodium-Glucose cotransporter 2 inhibitors) blocks glucose reabsorption in kidneys, and reduce the blood glucose. (7) GLP1-RA can inhibit appetite by the brain, contribute to decrease blood glucose (8–13).

Our results showed that except for DPP-4i, glucose-lowering drugs reduce FBS better than probiotics. A previous study indicated that administration of SGLT-2i and GLP-1RA can reduce blood glucose levels (51). However, the efficacy of DPP-4i for the management of the complications of type 2 diabetes remains unclear. Previous study have reported that different metabolic organs regulate diabetic hyperglycemia differently, and glucose-lowering drugs regulate blood glucose by targeting corresponding organs (Figure 6). In the present study, GLP-1RA appeared to be the most effective glucose-lowering drug for FBS reduction, followed by SGLT-2i and TZD; however, there was no significant difference between DPP-4i and placebo. A recent meta-analysis showed that the FBS-lowering efficacy of SGLT-2i was significantly greater than those of GLP-1RA and TZD (52), a finding that is inconsistent with our results. Meanwhile, our results showed that single species probiotics and multispecies probiotics lowered FBS better than placebo and DPP-4i. Several researchers have suggested that probiotic supplementation could regulate gut microbiota, improve the patient's glycaemic, lipid, and blood pressure metabolic profiles, and play an important role in type 2 diabetes (53–55). Thus, probiotics and DPP-4i may be considered for patients with relatively less severe hyperglycaemia.

It is well known that HbA1c is the most important biomarker of hyperglycaemia (13). We found that glucose-lowering drugs significantly decreased HbA1c level. We also noted that SGLT-2i induced the highest reduction of HbA1c. There was no significant difference between probiotics and placebo regarding HbA1c reduction. Monami et al. (56) indicated that compared with placebo, SGLT-2i are more effective for controlling HbA1c, a result that confirms the findings of the present study. In addition, we found that probiotic supplementation decreased the HOMA-IR index, regardless of whether the intervention was done using single species probiotics, multispecies probiotics, or probiotics with co-supplements; however, no significant effects on insulin were observed.

Interestingly, the results of the subgroup analysis indicated that participants aged > 55 years old showed higher reduction in FBS and HbA1c than those aged ≤ 55 years old, a finding that is inconsistent with that of our previous study (30). This variation may be attributed to the glucose-lowering drug intervention studies included in the present meta-analysis. Gan et al. (27) reported that the older people had longer durations of intervention and severe islet cell dysfunction, which may reduce the effects of antihyperglycemic drugs. Thus, it is still necessary to consider patients' ages when assessing the efficacies of glucose-lowering drugs in patients with type 2 diabetes, especially the elderly. Moreover, subjects with BMI ≥ 30 kg/m^2^ showed a significant decrease in FBS and the HOMA-IR index compared with those with lower BMI. Matteo et al. reported that lower BMI was related to better glycaemic control (57). However, several previous studies indicated that the efficacies of glucose-lowering drugs (58) and probiotics supplementation (59) appeared to increase as a function of baseline BMI. It is possible that weight loss induced by glucose-lowering drugs and probiotic supplementation plays an important role in glycaemic control in obese patients with type 2 diabetes. In addition, our results showed that subjects from Eastern countries had a higher reduction in FBS, HbA1c, and HOMA-IR than those from Western countries. Previous meta-meta-analyses have shown that blood glucose reduction is greater in Asian-dominant groups than in Caucasian-dominant groups (60, 61), a finding that confirms our results. The present study showed that the overall duration of intervention with glucose-lowering drugs (≥12 weeks) was longer than that of probiotics supplementation (≤ 12 weeks). These results illustrate that for probiotics, a longer duration of intervention may be required to induce gut microbiota changes and beneficial effects on glucose metabolism.

A recent meta-analysis indicated that different glucose-lowering agents can have varying impacts on the lipid profile. It has been reported that TZD significantly increase LDL-C and HDL-C levels while reducing TG (14, 62). It has been reported that DPP-4i can decrease TC (63). However, in the present study, there was no significant difference between the effects of glucose-lowering drugs (including TZD, GLP-1 RA, and DPP-4i) and placebos on all lipid indicators. In addition, it has been reported that the TC and TG levels of patients who consume multispecies probiotics are reduced, a result which is consistent with that of the present meta-analysis (30). Dong et al. (64) reported that intake of probiotics decreases LDL-C levels, but has no significant effect on TC, TG, and HDL-C levels. Wu et al. (65) reported that probiotic supplementation can lower TC and LDL-C levels, but has no significant effects on TG and HDL-C levels. The differences in these results may be due to differences in the patients' ages, BMI, race, and duration of intervention in the included studies. Our results also showed that DPP-4i significantly lowered TG level, whereas TZD reduced HDL-C level; however, further studies are needed to verify this finding. Furthermore, we found that participants of Eastern descent and those with baseline BMI <30 kg/m^2^ showed significantly higher reductions in TC and TG levels than participants of Western descent and those with BMI ≥ 30 kg/m^2^, respectively.

The use of lipid lowering medication like statin, and anti-hypertension medication like ACEI can influence lipid profiles and blood pressure much more than glucose-lowering drugs and probiotics. The use of those medication is quite common on T2D patients. Recently, the findings of several studies suggested that ACE inhibitors are highly associated with decrease in blood pressure (66–68). This may be attributed to the production of bioactive peptides that inhibit ACE and lead to a decrease in blood pressure. A previous study demonstrated that DPP-4i can induce reduction of blood pressure (SBP and DBP) (69). SGLT-2i have also been reported to significantly reduce blood pressure (70). However, the results of the present study indicate that glucose-lowering drugs have no significant effect on SBP and DBP, whereas probiotics decrease SBP and DBP, regardless of whether they are single species or multispecies probiotics, a finding which is similar to those of numerous studies (26, 71). However, no significant reduction blood pressure was observed in other studies after the intake of probiotics (25, 64). These differences in results may be due to differences in the races of participants. Our subgroup analysis showed that participants from Eastern countries had a higher decrease in blood pressure than those from Western countries. Further evidence is needed to confirm these findings.

Hypoglycaemia is a serious adverse reaction that cannot be ignored in patients with type 2 diabetes. It can influence a patient's quality of life and is associated with the highest incidence of cardiovascular disease (72, 73). In general, GLP-1 RA drugs do not induce hypoglycaemia unless they are combined with sulfonylureas (74). DPP-4i seldom induce hypoglycaemia in theory because they can regulate the secretion of insulin and glucagon (75). In the present study however, semaglutide, a long-acting GLP-1 analog (6), was associated with an increased risk for hypoglycaemia compared with a placebo. This indicates that the safety of semaglutide needs to be studied further. Linagliptin, a new DPP-4i, has no significant effect on the risk for hypoglycaemia. Thus, DPP-4i may be considered safe for patients with type 2 diabetes. In addition, since the effects of GLP-1 RA and DPP-4i on hypoglycaemia were assessed in only one of the included studies, the difference between the risks for hypoglycaemia associated with glucose-lowering drugs and placebos needs to be clarified.

To our knowledge, this is the first meta-analysis in which the hypoglycaemic effects of probiotic supplementation and those of glucose-lowering drugs were compared. However, this study has several limitations. First, the protocol of our meta-analysis was not registered in PROSPERO. Second, due to the paucity of studies that satisfied the inclusion and exclusion criteria, TZD and SGLT-2i intervention studies were few. This may make the results unreliable. Therefore, future RCTs in which the efficacies of these agents are directly compared are needed. Third, the subgroup analysis of dose that is necessary to evaluate the effect of probiotic supplementation on indicators associated with glucose, lipids, and blood pressure metabolism. At the same time, subgroup analysis is necessary with the sample size and female/male numbers, due to there were different numbers in included studies. Besides, dietary and physical activity also plays an important role in effects of glucose-lowering drugs and probiotics, so these confounders should take it into consideration in furture study. Forth, other traditional hypoglycemic drugs, like metformin, sulfonylureas, alpha-glucosidase inhibitors, and insulin should be also included in furture study. Finally, due to differences in study baseline characteristics, such as age, BMI, disease duration, country, and duration of intervention, there was high heterogeneity in some indicators. As these factors may influence the overall pooled results, larger trials are needed to support the results of this meta-analysis.

## Conclusions

In the summary, the results of this meta-analysis suggest some relevant recommendations for the use of probiotic supplementation and glucose-lowering drugs for the treatment of patients with type 2 diabetes. GLP-1 RA may be optimal for the reduction of FBS, followed by SGLT-2i and TZD. Administration of SGLT2i may be a good treatment option for reduction of HbA1c level. TZD, DPP-4i, and GLP-1 RA can also lower HbA1c level. However, probiotic supplementation, especially multispecies probiotics, might be a good choice for reduction of glycemia indexes (FBS, HbA1c and HOMA-IR), lipid profile indicators (TC and TG) and blood pressure (SBP and DBP). Thus, the effect of probiotics in reduction of glycaemic indexes (FBS, HbA1c) is much milder than glucose-lowering drugs, but more effective in improving other metabolic index (lipid profiles and hypertension). So probiotics is fit for less severe T2D patients. T2D patients with high severity should still consider glucose-lowering drugs as primary treatment. Results of this review might be able to give an additional contribute to the field working onto alternative strategies aimed to reduce the use of drugs.

## Data availability statement

The original contributions presented in the study are included in the article/Supplementary material, further inquiries can be directed to the corresponding author/s.

## Author contributions

TL, XX, LW, LL, LY, and HG designed the research and wrote the paper. ZD, XZ, XC, JZ, YD, and QW conducted the research, analyzed the data, and performed the statistical analysis. All authors had equal responsibility for the final content of the paper, read, and agreed to the published version of the manuscript.

## Funding

This study was supported by research grants from the GDAS Special Project of Capacity Building for Innovation-driven Development (2019GDASYL-0201001), the Guangdong Province Academy of Sciences Special Project for Capacity Building of Innovation Driven Development (2020GDASYL-20200301002), and National Key Research and Development Program (Application demonstration of Key Technologies for Food Safety Emergency Assurance) (2019YFC160630501).

## Conflict of interest

The authors declare that the research was conducted in the absence of any commercial or financial relationships that could be construed as a potential conflict of interest.

## Publisher's note

All claims expressed in this article are solely those of the authors and do not necessarily represent those of their affiliated organizations, or those of the publisher, the editors and the reviewers. Any product that may be evaluated in this article, or claim that may be made by its manufacturer, is not guaranteed or endorsed by the publisher.

## Supplementary material

Supplementary Figure 1 Forest plot for the effect of probiotics supplementation and glucose-lowering drugs on diarrhea **(A)** and hypoglycemia **(B)** compared to placebo. For each study, the solid black diamonds represent the point estimate of the intervention effect. The horizontal line joins the lower and upper limits of the 95% CI of this effect. The open diamonds represent the subgroup and overall SMD determined with a random-effects model.

Supplementary Figure 2 Subgroup analysis of the subject ages, BMI, country, and duration of intervention on the effect of FBS levels of probiotics supplementation and glucose-lowering drugs. Ages **(A)**, BMI **(B)**, country **(C)**, and duration of intervention **(D)**. For each study, the solid black diamonds represent the point estimate of the intervention effect. The horizontal line joins the lower and upper limits of the 95% CI of this effect. The open diamonds represent the subgroup and overall SMD determined with a random-effects model.

Supplementary Figure 3 Subgroup analysis of the subject ages, BMI, country, and duration of intervention on the effect of HbA1c levels of probiotics supplementation and glucose-lowering drugs. Ages **(A)**, BMI **(B)**, country **(C)**, and duration of intervention **(D)**. For each study, the solid black diamonds represent the point estimate of the intervention effect. The horizontal line joins the lower and upper limits of the 95% CI of this effect. The open diamonds represent the subgroup and overall SMD determined with a random-effects model.

Supplementary Figure 4 Subgroup analysis of the subject ages, BMI, country, and duration of intervention on the effect of insulin levels of probiotics supplementation and glucose-lowering drugs. Ages **(A)**, BMI **(B)**, country **(C)**, and duration of intervention **(D)**. For each study, the solid black diamonds represent the point estimate of the intervention effect. The horizontal line joins the lower and upper limits of the 95% CI of this effect. The open diamonds represent the subgroup and overall SMD determined with a random-effects model.

Supplementary Figure 5 Subgroup analysis of the subject ages, BMI, country, and duration of intervention on the effect of HOMA-IR levels of probiotics supplementation and glucose-lowering drugs. Ages **(A)**, BMI **(B)**, country **(C)**, and duration of intervention **(D)**. For each study, the solid black diamonds represent the point estimate of the intervention effect. The horizontal line joins the lower and upper limits of the 95% CI of this effect. The open diamonds represent the subgroup and overall SMD determined with a random-effects model.

Supplementary Figure 6 Subgroup analysis of the subject ages, BMI, country, and duration of intervention on the effect of TC levels of probiotics supplementation and glucose-lowering drugs. Ages **(A)**, BMI **(B)**, country **(C)**, and duration of intervention **(D)**. For each study, the solid black diamonds represent the point estimate of the intervention effect. The horizontal line joins the lower and upper limits of the 95% CI of this effect. The open diamonds represent the subgroup and overall SMD determined with a random-effects model.

Supplementary Figure 7 Subgroup analysis of the subject ages, BMI, country, and duration of intervention on the effect of TG levels of probiotics supplementation and glucose-lowering drugs. Ages **(A)**, BMI **(B)**, country **(C)**, and duration of intervention **(D)**. For each study, the solid black diamonds represent the point estimate of the intervention effect. The horizontal line joins the lower and upper limits of the 95% CI of this effect. The open diamonds represent the subgroup and overall SMD determined with a random-effects model.

Supplementary Figure 8 Subgroup analysis of the subject ages, BMI, country, and duration of intervention on the effect of HDL-C levels of probiotics supplementation and glucose-lowering drugs. Ages **(A)**, BMI **(B)**, country **(C)**, and duration of intervention **(D)**. For each study, the solid black diamonds represent the point estimate of the intervention effect. The horizontal line joins the lower and upper limits of the 95% CI of this effect. The open diamonds represent the subgroup and overall SMD determined with a random-effects model.

Supplementary Figure 9 Subgroup analysis of the subject ages, BMI, country, and duration of intervention on the effect of LDL-C levels of probiotics supplementation and glucose-lowering drugs. Ages **(A)**, BMI **(B)**, country **(C)**, and duration of intervention **(D)**. For each study, the solid black diamonds represent the point estimate of the intervention effect. The horizontal line joins the lower and upper limits of the 95% CI of this effect. The open diamonds represent the subgroup and overall SMD determined with a random-effects model.

Supplementary Figure 10 Subgroup analysis of the subject ages, BMI, country, and duration of intervention on the effect of SBP levels of probiotics supplementation and glucose-lowering drugs. Ages **(A)**, BMI **(B)**, country **(C)**, and duration of intervention **(D)**. For each study, the solid black diamonds represent the point estimate of the intervention effect. The horizontal line joins the lower and upper limits of the 95% CI of this effect. The open diamonds represent the subgroup and overall SMD determined with a random-effects model.

Supplementary Figure 11 Subgroup analysis of the subject ages, BMI, country, and duration of intervention on the effect of DBP levels of probiotics supplementation and glucose-lowering drugs. Ages **(A)**, BMI **(B)**, country **(C)**, and duration of intervention **(D)**. For each study, the solid black diamonds represent the point estimate of the intervention effect. The horizontal line joins the lower and upper limits of the 95% CI of this effect. The open diamonds represent the subgroup and overall SMD determined with a random-effects model.

Supplementary Figure 12 Begg's funnel plot for publication bias in trials on the effect of probiotics and glucose-lowering drugs on glycemia indexes [FBS **(A)**, HbA1c **(B)**, Insulin **(C)**, HOMA-IR **(D)**].

Supplementary Figure 13 Egger's test in quantitative for publication bias in trials on the effect of probiotics and glucose-lowering drugs on glycemia indexes [FBS **(A)**, HbA1c **(B)**, Insulin **(C)**, HOMA-IR **(D)**].

Supplementary Figure 14 Begg's funnel plot for publication bias in trials on the effect of probiotics and glucose-lowering drugs on lipid indexes [TC **(A)**, TG **(B)**, HDL-C **(C)**, LDL-C **(D)**].

Supplementary Figure 15 Egger's test in quantitative for publication bias in trials on the effect of probiotics and glucose-lowering drugs on lipid indexes [TC **(A)**, TG **(B)**, HDL-C **(C)**, LDL-C **(D)**].

Supplementary Figure 16 Begg's funnel plot for publication bias in trials on the effect of probiotics and glucose-lowering drugs on blood pressure indexes [SBP **(A)**, DBP **(B)**].

Supplementary Figure 17 Egger's test in quantitative for publication bias in trials on the effect of probiotics and glucose-lowering drugs on blood pressure indexes [SBP **(A)**, DBP **(B)**].

Supplementary Figure 18 The sensitivity analysis of individual studies was performed by evaluating each study on the changes overall results of the meta-analysis in glycemia indicators [FBS **(A)**, HbA1c **(B)**, Insulin **(C)**, HOMA-IR **(D)**].

Supplementary Figure 19 The sensitivity analysis of individual studies was performed by evaluating each study on the changes overall results of the meta-analysis in blood lipids indicators [TC **(A)**, TG **(B)**, HDL-C **(C)**, LDL-C **(D)**].

Supplementary Figure 20 The sensitivity analysis of individual studies was performed by evaluating each study on the changes overall results of the meta-analysis in blood pressure indicators [SBP **(A)**, DBP **(B)**].

Supplementary Table 1 The search terms for each database.

Supplementary Table 2 PICOS criteria for inclusion and exclusion of studies.

Supplementary Table 3 PRISMA 2015 Checklist. This checklist has been adapted for use with systematic review protocol submissions to Food reviews international.
