# Supplementary material for: Comparative analysis of the efficacies of probiotic supplementation and glucose-lowering drugs for the treatment of type 2 diabetes: A systematic review and meta-analysis
Source: Front Nutr. 2022 Jul 18;9:825897. doi: 10.3389/fnut.2022.825897 (PMC9339904; doi:10.3389/fnut.2022.825897)
Supplement: Supplementary Figure 1 — Forest plot for the effect of probiotics supplementation and glucose-lowering drugs on diarrhea (A) and hypoglycemia (B) compared to placebo. For each study, the solid black diamonds represent the point estimate of the intervention effect. The horizontal line joins the lower and upper limits of the 95% CI of this effect. The open diamonds represent the subgroup and overall SMD determined with a random-effects model. [file Presentation_1.pdf]

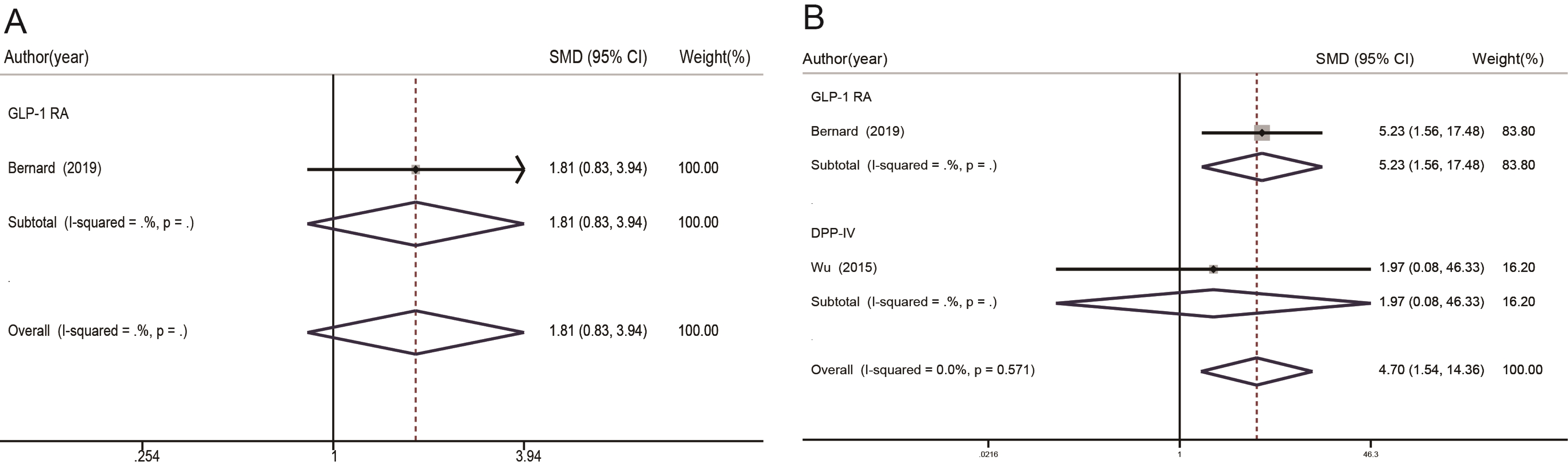
A
Author(year)
SMD (95% CI)
Weight(%)
GLP-1 RA
Bernard (2019)
1.81 (0.83, 3.94)
100.00
Subtotal (I-squared = .%, p = .)
1.81 (0.83, 3.94)
100.00
Overall (I-squared = .%, p = .)
1.81 (0.83, 3.94)
100.00
.254
1
3.94
B
Author(year)
SMD (95% CI)
Weight(%)
GLP-1 RA
Bernard (2019)
5.23 (1.56, 17.48)
83.80
Subtotal (I-squared = .%, p = .)
5.23 (1.56, 17.48)
83.80
DPP-IV
Wu (2015)
1.97 (0.08, 46.33)
16.20
Subtotal (I-squared = .%, p = .)
1.97 (0.08, 46.33)
16.20
Overall (I-squared = 0.0%, p = 0.571)
4.70 (1.54, 14.36)
100.00
.0216
1
46.3

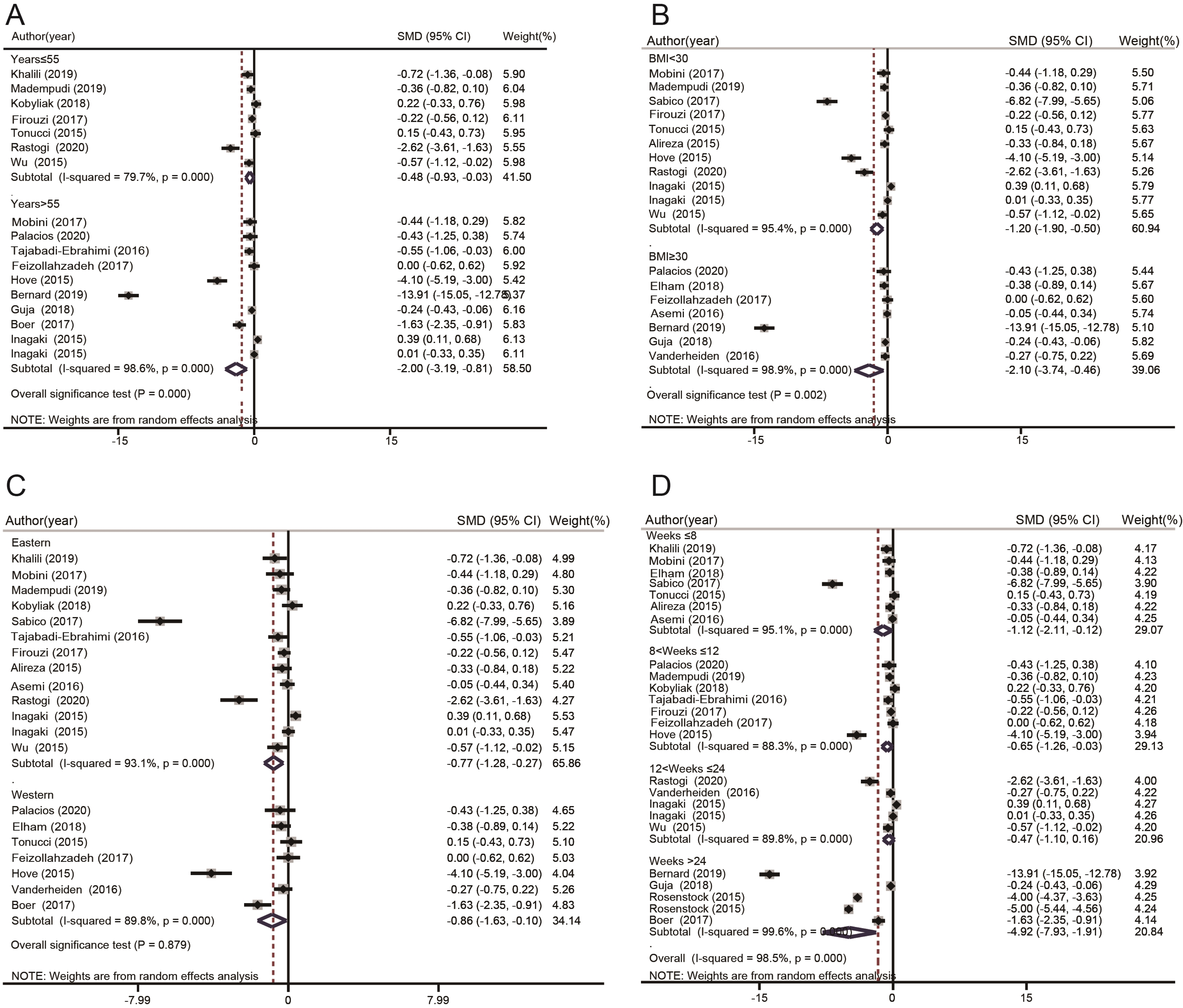
A
Author(year) SMD (95% CI) Weight(%)
Years≤55
Khalili (2019) -0.72 (-1.36, -0.08) 5.90
Madempudi (2019) -0.36 (-0.82, 0.10) 6.04
Kobyliak (2018) 0.22 (-0.33, 0.76) 5.98
Firouzi (2017) -0.22 (-0.56, 0.12) 6.11
Tonucci (2015) 0.15 (-0.43, 0.73) 5.95
Rastogi (2020) -2.62 (-3.61, -1.63) 5.55
Wu (2015) -0.57 (-1.12, -0.02) 5.98
Subtotal (I-squared = 79.7%, p = 0.000) -0.48 (-0.93, -0.03) 41.50
Years>55
Mobini (2017) -0.44 (-1.18, 0.29) 5.82
Palacios (2020) -0.43 (-1.25, 0.38) 5.74
Tajabadi-Ebrahimi (2016) -0.55 (-1.06, -0.03) 6.00
Feizollahzadeh (2017) 0.00 (-0.62, 0.62) 5.92
Hove (2015) -4.10 (-5.19, -3.00) 5.42
Bernard (2019) -13.91 (-15.05, -12.78) 5.37
Guja (2018) -0.24 (-0.43, -0.06) 6.16
Boer (2017) -1.63 (-2.35, -0.91) 5.83
Inagaki (2015) 0.39 (0.11, 0.68) 6.13
Inagaki (2015) 0.01 (-0.33, 0.35) 6.11
Subtotal (I-squared = 98.6%, p = 0.000) -2.00 (-3.19, -0.81) 58.50
Overall significance test (P = 0.000)
NOTE: Weights are from random effects analysis
-15
0
15
B
Author(year) SMD (95% CI) Weight(%)
BMI<30
Mobini (2017) -0.44 (-1.18, 0.29) 5.50
Madempudi (2019) -0.36 (-0.82, 0.10) 5.71
Sabico (2017) -6.82 (-7.99, -5.65) 5.06
Firouzi (2017) -0.22 (-0.56, 0.12) 5.77
Tonucci (2015) 0.15 (-0.43, 0.73) 5.63
Alireza (2015) -0.33 (-0.84, 0.18) 5.67
Hove (2015) -4.10 (-5.19, -3.00) 5.14
Rastogi (2020) -2.62 (-3.61, -1.63) 5.26
Inagaki (2015) 0.39 (0.11, 0.68) 5.79
Inagaki (2015) 0.01 (-0.33, 0.35) 5.77
Wu (2015) -0.57 (-1.12, -0.02) 5.65
Subtotal (I-squared = 95.4%, p = 0.000) -1.20 (-1.90, -0.50) 60.94
BMI≥30
Palacios (2020) -0.43 (-1.25, 0.38) 5.44
Elham (2018) -0.38 (-0.89, 0.14) 5.67
Feizollahzadeh (2017) 0.00 (-0.62, 0.62) 5.60
Asemi (2016) -0.05 (-0.44, 0.34) 5.74
Bernard (2019) -13.91 (-15.05, -12.78) 5.10
Guja (2018) -0.24 (-0.43, -0.06) 5.82
Vanderheiden (2016) -0.27 (-0.75, 0.22) 5.69
Subtotal (I-squared = 98.9%, p = 0.000) -2.10 (-3.74, -0.46) 39.06
Overall significance test (P = 0.002)
NOTE: Weights are from random effects analysis
-15
0
15
C
Author(year) SMD (95% CI) Weight(%)
Eastern
Khalili (2019) -0.72 (-1.36, -0.08) 4.99
Mobini (2017) -0.44 (-1.18, 0.29) 4.80
Madempudi (2019) -0.36 (-0.82, 0.10) 5.30
Kobyliak (2018) 0.22 (-0.33, 0.76) 5.16
Sabico (2017) -6.82 (-7.99, -5.65) 3.89
Tajabadi-Ebrahimi (2016) -0.55 (-1.06, -0.03) 5.21
Firouzi (2017) -0.22 (-0.56, 0.12) 5.47
Alireza (2015) -0.33 (-0.84, 0.18) 5.22
Asemi (2016) -0.05 (-0.44, 0.34) 5.40
Rastogi (2020) -2.62 (-3.61, -1.63) 4.27
Inagaki (2015) 0.39 (0.11, 0.68) 5.53
Inagaki (2015) 0.01 (-0.33, 0.35) 5.47
Wu (2015) -0.57 (-1.12, -0.02) 5.15
Subtotal (I-squared = 93.1%, p = 0.000) -0.77 (-1.28, -0.27) 65.86
Western
Palacios (2020) -0.43 (-1.25, 0.38) 4.65
Elham (2018) -0.38 (-0.89, 0.14) 5.22
Tonucci (2015) 0.15 (-0.43, 0.73) 5.10
Feizollahzadeh (2017) 0.00 (-0.62, 0.62) 5.03
Hove (2015) -4.10 (-5.19, -3.00) 4.04
Vanderheiden (2016) -0.27 (-0.75, 0.22) 5.26
Boer (2017) -1.63 (-2.35, -0.91) 4.83
Subtotal (I-squared = 89.8%, p = 0.000) -0.86 (-1.63, -0.10) 34.14
Overall significance test (P = 0.879)
NOTE: Weights are from random effects analysis
-7.99
0
7.99
D
Author(year) SMD (95% CI) Weight(%)
Weeks ≤8
Khalili (2019) -0.72 (-1.36, -0.08) 4.17
Mobini (2017) -0.44 (-1.18, 0.29) 4.13
Elham (2018) -0.38 (-0.89, 0.14) 4.22
Sabico (2017) -6.82 (-7.99, -5.65) 3.90
Tonucci (2015) 0.15 (-0.43, 0.73) 4.19
Alireza (2015) -0.33 (-0.84, 0.18) 4.22
Asemi (2016) -0.05 (-0.44, 0.34) 4.25
Subtotal (I-squared = 95.1%, p = 0.000) -1.12 (-2.11, -0.12) 29.07
8<Weeks ≤12
Palacios (2020) -0.43 (-1.25, 0.38) 4.10
Madempudi (2019) -0.36 (-0.82, 0.10) 4.23
Kobyliak (2018) 0.22 (-0.33, 0.76) 4.20
Tajabadi-Ebrahimi (2016) -0.55 (-1.06, -0.03) 4.21
Firouzi (2017) -0.22 (-0.56, 0.12) 4.26
Feizollahzadeh (2017) 0.00 (-0.62, 0.62) 4.18
Hove (2015) -4.10 (-5.19, -3.00) 3.94
Subtotal (I-squared = 88.3%, p = 0.000) -0.65 (-1.26, -0.03) 29.13
12<Weeks ≤24
Rastogi (2020) -2.62 (-3.61, -1.63) 4.00
Vanderheiden (2016) -0.27 (-0.75, 0.22) 4.22
Inagaki (2015) 0.39 (0.11, 0.68) 4.27
Inagaki (2015) 0.01 (-0.33, 0.35) 4.26
Wu (2015) -0.57 (-1.12, -0.02) 4.20
Subtotal (I-squared = 89.8%, p = 0.000) -0.47 (-1.10, 0.16) 20.96
Weeks >24
Bernard (2019) -13.91 (-15.05, -12.78) 3.92
Guja (2018) -0.24 (-0.43, -0.06) 4.29
Rosenstock (2015) -4.00 (-4.37, -3.63) 4.25
Rosenstock (2015) -5.00 (-5.44, -4.56) 4.24
Boer (2017) -1.63 (-2.35, -0.91) 4.14
Subtotal (I-squared = 99.6%, p = 0.000) -4.92 (-7.93, -1.91) 20.84
Overall (I-squared = 98.5%, p = 0.000)
NOTE: Weights are from random effects analysis
-15
0
15

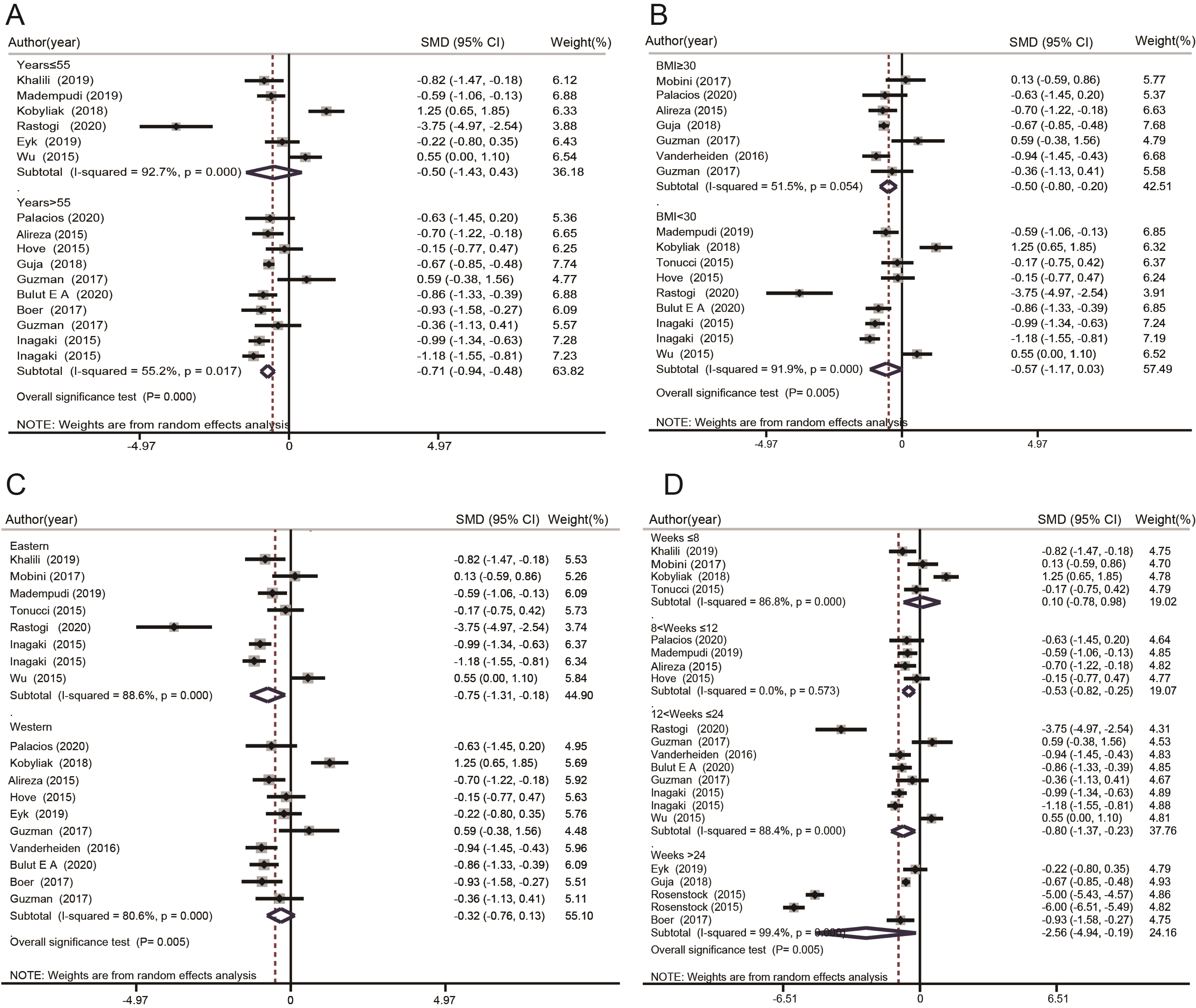
A
Author(year)
SMD (95% CI)
Weight(%)
Years≤55
Khalili (2019) -0.82 (-1.47, -0.18) 6.12
Madempudi (2019) -0.59 (-1.06, -0.13) 6.88
Kobyliak (2018) 1.25 (0.65, 1.85) 6.33
Rastogi (2020) -3.75 (-4.97, -2.54) 3.88
Eyk (2019) -0.22 (-0.80, 0.35) 6.43
Wu (2015) 0.55 (0.00, 1.10) 6.54
Subtotal (I-squared = 92.7%, p = 0.000) -0.50 (-1.43, 0.43) 36.18
Years>55
Palacios (2020) -0.63 (-1.45, 0.20) 5.36
Alireza (2015) -0.70 (-1.22, -0.18) 6.65
Hove (2015) -0.15 (-0.77, 0.47) 6.25
Guja (2018) -0.67 (-0.85, -0.48) 7.74
Guzman (2017) 0.59 (-0.38, 1.56) 4.77
Bulut E A (2020) -0.86 (-1.33, -0.39) 6.88
Boer (2017) -0.93 (-1.58, -0.27) 6.09
Guzman (2017) -0.36 (-1.13, 0.41) 5.57
Inagaki (2015) -0.99 (-1.34, -0.63) 7.28
Inagaki (2015) -1.18 (-1.55, -0.81) 7.23
Subtotal (I-squared = 55.2%, p = 0.017) -0.71 (-0.94, -0.48) 63.82
Overall significance test (P= 0.000)
NOTE: Weights are from random effects analysis
-4.97
0
4.97
B
Author(year)
SMD (95% CI)
Weight(%)
BMI≥30
Mobini (2017) 0.13 (-0.59, 0.86) 5.77
Palacios (2020) -0.63 (-1.45, 0.20) 5.37
Alireza (2015) -0.70 (-1.22, -0.18) 6.63
Guja (2018) -0.67 (-0.85, -0.48) 7.68
Guzman (2017) 0.59 (-0.38, 1.56) 4.79
Vanderheiden (2016) -0.94 (-1.45, -0.43) 6.68
Guzman (2017) -0.36 (-1.13, 0.41) 5.58
Subtotal (I-squared = 51.5%, p = 0.054) -0.50 (-0.80, -0.20) 42.51
BMI<30
Madempudi (2019) -0.59 (-1.06, -0.13) 6.85
Kobyliak (2018) 1.25 (0.65, 1.85) 6.32
Tonucci (2015) -0.17 (-0.75, 0.42) 6.37
Hove (2015) -0.15 (-0.77, 0.47) 6.24
Rastogi (2020) -3.75 (-4.97, -2.54) 3.91
Bulut E A (2020) -0.86 (-1.33, -0.39) 6.85
Inagaki (2015) -0.99 (-1.34, -0.63) 7.24
Inagaki (2015) -1.18 (-1.55, -0.81) 7.19
Wu (2015) 0.55 (0.00, 1.10) 6.52
Subtotal (I-squared = 91.9%, p = 0.000) -0.57 (-1.17, 0.03) 57.49
Overall significance test (P= 0.005)
NOTE: Weights are from random effects analysis
-4.97
0
4.97
C
Author(year)
SMD (95% CI)
Weight(%)
Eastern
Khalili (2019) -0.82 (-1.47, -0.18) 5.53
Mobini (2017) 0.13 (-0.59, 0.86) 5.26
Madempudi (2019) -0.59 (-1.06, -0.13) 6.09
Tonucci (2015) -0.17 (-0.75, 0.42) 5.73
Rastogi (2020) -3.75 (-4.97, -2.54) 3.74
Inagaki (2015) -0.99 (-1.34, -0.63) 6.37
Inagaki (2015) -1.18 (-1.55, -0.81) 6.34
Wu (2015) 0.55 (0.00, 1.10) 5.84
Subtotal (I-squared = 88.6%, p = 0.000) -0.75 (-1.31, -0.18) 44.90
Western
Palacios (2020) -0.63 (-1.45, 0.20) 4.95
Kobyliak (2018) 1.25 (0.65, 1.85) 5.69
Alireza (2015) -0.70 (-1.22, -0.18) 5.92
Hove (2015) -0.15 (-0.77, 0.47) 5.63
Eyk (2019) -0.22 (-0.80, 0.35) 5.76
Guzman (2017) 0.59 (-0.38, 1.56) 4.48
Vanderheiden (2016) -0.94 (-1.45, -0.43) 5.96
Bulut E A (2020) -0.86 (-1.33, -0.39) 6.09
Boer (2017) -0.93 (-1.58, -0.27) 5.51
Guzman (2017) -0.36 (-1.13, 0.41) 5.11
Subtotal (I-squared = 80.6%, p = 0.000) -0.32 (-0.76, 0.13) 55.10
Overall significance test (P= 0.005)
NOTE: Weights are from random effects analysis
-4.97
0
4.97
D
Author(year)
SMD (95% CI)
Weight(%)
Weeks ≤8
Khalili (2019) -0.82 (-1.47, -0.18) 4.75
Mobini (2017) 0.13 (-0.59, 0.86) 4.70
Kobyliak (2018) 1.25 (0.65, 1.85) 4.78
Tonucci (2015) -0.17 (-0.75, 0.42) 4.79
Subtotal (I-squared = 86.8%, p = 0.000) 0.10 (-0.78, 0.98) 19.02
8<Weeks ≤12
Palacios (2020) -0.63 (-1.45, 0.20) 4.64
Madempudi (2019) -0.59 (-1.06, -0.13) 4.85
Alireza (2015) -0.70 (-1.22, -0.18) 4.82
Hove (2015) -0.15 (-0.77, 0.47) 4.77
Subtotal (I-squared = 0.0%, p = 0.573) -0.53 (-0.82, -0.25) 19.07
12<Weeks ≤24
Rastogi (2020) -3.75 (-4.97, -2.54) 4.31
Guzman (2017) 0.59 (-0.38, 1.56) 4.53
Vanderheiden (2016) -0.94 (-1.45, -0.43) 4.83
Bulut E A (2020) -0.86 (-1.33, -0.39) 4.85
Guzman (2017) -0.36 (-1.13, 0.41) 4.67
Inagaki (2015) -0.99 (-1.34, -0.63) 4.89
Inagaki (2015) -1.18 (-1.55, -0.81) 4.88
Wu (2015) 0.55 (0.00, 1.10) 4.81
Subtotal (I-squared = 88.4%, p = 0.000) -0.80 (-1.37, -0.23) 37.76
Weeks >24
Eyk (2019) -0.22 (-0.80, 0.35) 4.79
Guja (2018) -0.67 (-0.85, -0.48) 4.93
Rosenstock (2015) -5.00 (-5.43, -4.57) 4.86
Rosenstock (2015) -6.00 (-6.51, -5.49) 4.82
Boer (2017) -0.93 (-1.58, -0.27) 4.75
Subtotal (I-squared = 99.4%, p = 0.000) -2.56 (-4.94, -0.19) 24.16
Overall significance test (P= 0.005)
NOTE: Weights are from random effects analysis
-6.51
0
6.51

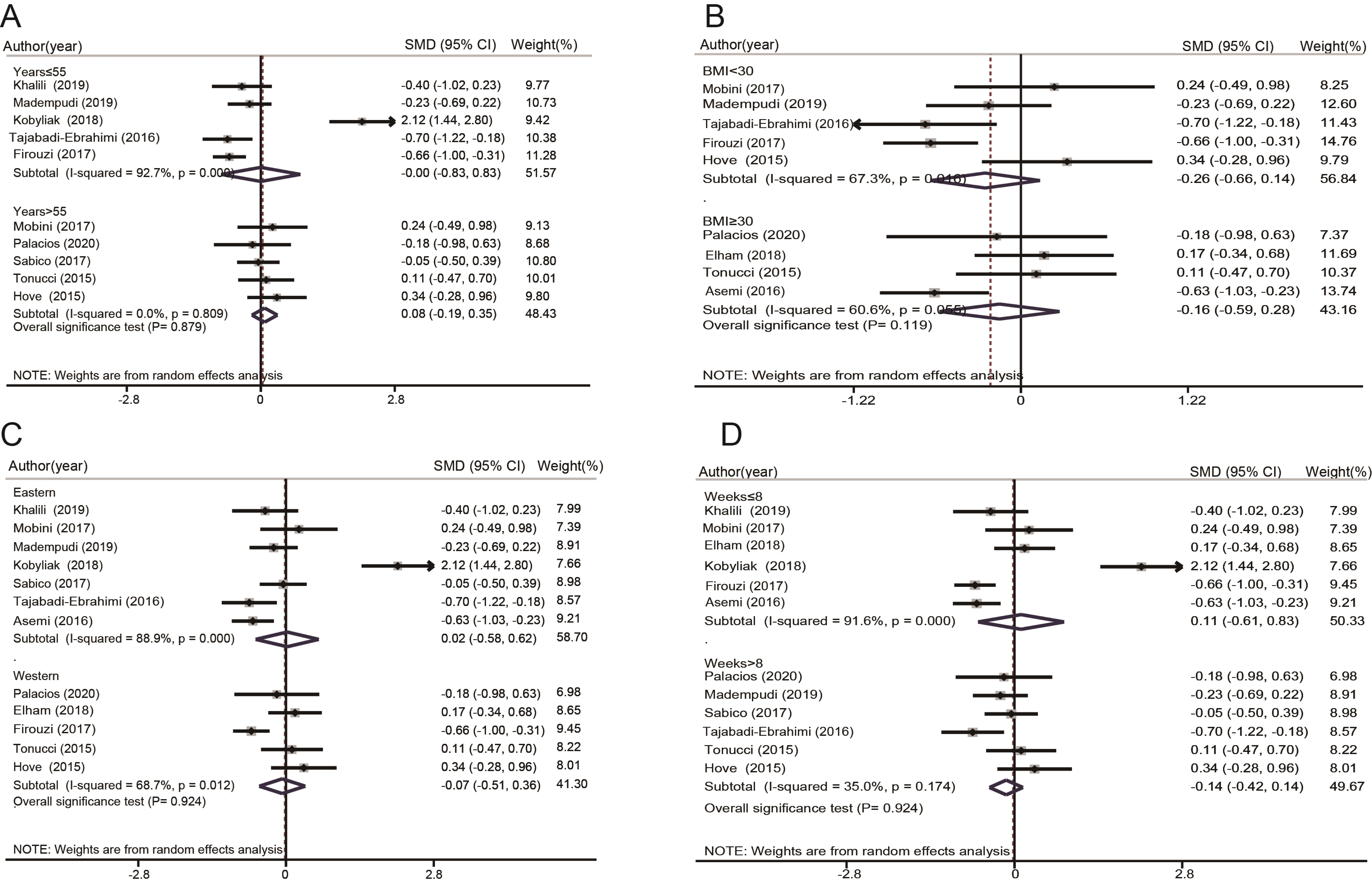
A
Author(year)
SMD (95% CI)
Weight(%)
Years≤55
Khalili (2019) -0.40 (-1.02, 0.23) 9.77
Madempudi (2019) -0.23 (-0.69, 0.22) 10.73
Kobyliak (2018) 2.12 (1.44, 2.80) 9.42
Tajabadi-Ebrahimi (2016) -0.70 (-1.22, -0.18) 10.38
Firouzi (2017) -0.66 (-1.00, -0.31) 11.28
Subtotal (I-squared = 92.7%, p = 0.000) -0.00 (-0.83, 0.83) 51.57
Years>55
Mobini (2017) 0.24 (-0.49, 0.98) 9.13
Palacios (2020) -0.18 (-0.98, 0.63) 8.68
Sabico (2017) -0.05 (-0.50, 0.39) 10.80
Tonucci (2015) 0.11 (-0.47, 0.70) 10.01
Hove (2015) 0.34 (-0.28, 0.96) 9.80
Subtotal (I-squared = 0.0%, p = 0.809) 0.08 (-0.19, 0.35) 48.43
Overall significance test (P= 0.879)
NOTE: Weights are from random effects analysis
-2.8
0
2.8
B
Author(year)
SMD (95% CI)
Weight(%)
BMI<30
Mobini (2017) 0.24 (-0.49, 0.98) 8.25
Madempudi (2019) -0.23 (-0.69, 0.22) 12.60
Tajabadi-Ebrahimi (2016) -0.70 (-1.22, -0.18) 11.43
Firouzi (2017) -0.66 (-1.00, -0.31) 14.76
Hove (2015) 0.34 (-0.28, 0.96) 9.79
Subtotal (I-squared = 67.3%, p = 0.016) -0.26 (-0.66, 0.14) 56.84
BMI≥30
Palacios (2020) -0.18 (-0.98, 0.63) 7.37
Elham (2018) 0.17 (-0.34, 0.68) 11.69
Tonucci (2015) 0.11 (-0.47, 0.70) 10.37
Asemi (2016) -0.63 (-1.03, -0.23) 13.74
Subtotal (I-squared = 60.6%, p = 0.055) -0.16 (-0.59, 0.28) 43.16
Overall significance test (P= 0.119)
NOTE: Weights are from random effects analysis
-1.22
0
1.22
C
Author(year)
SMD (95% CI)
Weight(%)
Eastern
Khalili (2019) -0.40 (-1.02, 0.23) 7.99
Mobini (2017) 0.24 (-0.49, 0.98) 7.39
Madempudi (2019) -0.23 (-0.69, 0.22) 8.91
Kobyliak (2018) 2.12 (1.44, 2.80) 7.66
Sabico (2017) -0.05 (-0.50, 0.39) 8.98
Tajabadi-Ebrahimi (2016) -0.70 (-1.22, -0.18) 8.57
Asemi (2016) -0.63 (-1.03, -0.23) 9.21
Subtotal (I-squared = 88.9%, p = 0.000) 0.02 (-0.58, 0.62) 58.70
Western
Palacios (2020) -0.18 (-0.98, 0.63) 6.98
Elham (2018) 0.17 (-0.34, 0.68) 8.65
Firouzi (2017) -0.66 (-1.00, -0.31) 9.45
Tonucci (2015) 0.11 (-0.47, 0.70) 8.22
Hove (2015) 0.34 (-0.28, 0.96) 8.01
Subtotal (I-squared = 68.7%, p = 0.012) -0.07 (-0.51, 0.36) 41.30
Overall significance test (P= 0.924)
NOTE: Weights are from random effects analysis
-2.8
0
2.8
D
Author(year)
SMD (95% CI)
Weight(%)
Weeks≤8
Khalili (2019) -0.40 (-1.02, 0.23) 7.99
Mobini (2017) 0.24 (-0.49, 0.98) 7.39
Elham (2018) 0.17 (-0.34, 0.68) 8.65
Kobyliak (2018) 2.12 (1.44, 2.80) 7.66
Firouzi (2017) -0.66 (-1.00, -0.31) 9.45
Asemi (2016) -0.63 (-1.03, -0.23) 9.21
Subtotal (I-squared = 91.6%, p = 0.000) 0.11 (-0.61, 0.83) 50.33
Weeks>8
Palacios (2020) -0.18 (-0.98, 0.63) 6.98
Madempudi (2019) -0.23 (-0.69, 0.22) 8.91
Sabico (2017) -0.05 (-0.50, 0.39) 8.98
Tajabadi-Ebrahimi (2016) -0.70 (-1.22, -0.18) 8.57
Tonucci (2015) 0.11 (-0.47, 0.70) 8.22
Hove (2015) 0.34 (-0.28, 0.96) 8.01
Subtotal (I-squared = 35.0%, p = 0.174) -0.14 (-0.42, 0.14) 49.67
Overall significance test (P= 0.924)
NOTE: Weights are from random effects analysis
-2.8
0
2.8

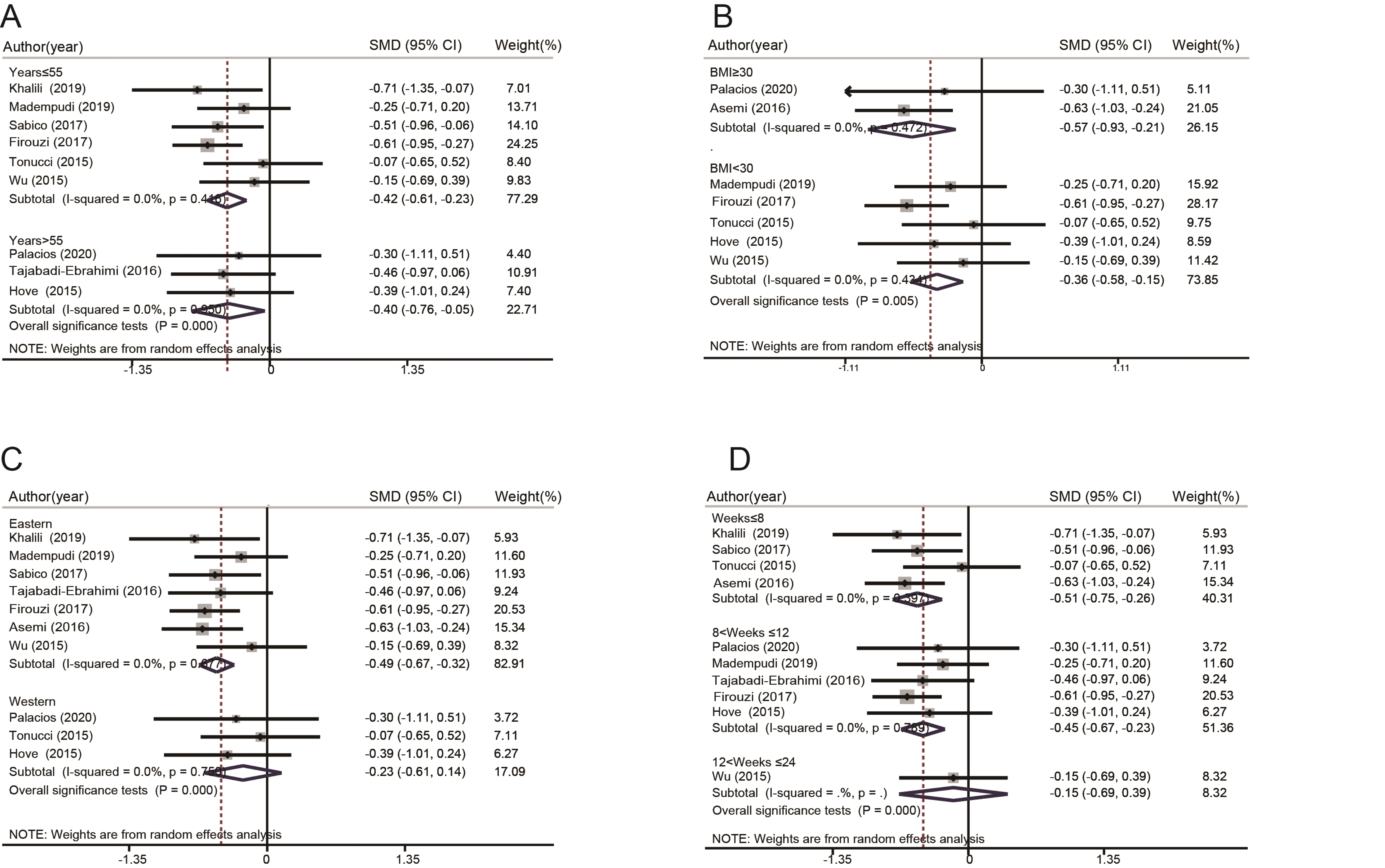
A
Author(year)
SMD (95% CI)
Weight(%)
Years≤55
Khalili (2019) -0.71 (-1.35, -0.07) 7.01
Madempudi (2019) -0.25 (-0.71, 0.20) 13.71
Sabico (2017) -0.51 (-0.96, -0.06) 14.10
Firouzi (2017) -0.61 (-0.95, -0.27) 24.25
Tonucci (2015) -0.07 (-0.65, 0.52) 8.40
Wu (2015) -0.15 (-0.69, 0.39) 9.83
Subtotal (I-squared = 0.0%, p = 0.41 -0.42 (-0.61, -0.23) 77.29
Years>55
Palacios (2020) -0.30 (-1.11, 0.51) 4.40
Tajabadi-Ebrahimi (2016) -0.46 (-0.97, 0.06) 10.91
Hove (2015) -0.39 (-1.01, 0.24) 7.40
Subtotal (I-squared = 0.0%, p = -0.40 (-0.76, -0.05) 22.71
Overall significance tests (P = 0.000)
NOTE: Weights are from random effects analysis
-1.35
0
1.35
B
Author(year)
SMD (95% CI)
Weight(%)
BMI≥30
Palacios (2020) -0.30 (-1.11, 0.51) 5.11
Asemi (2016) -0.63 (-1.03, -0.24) 21.05
Subtotal (I-squared = 0.0%, p = 0.472) -0.57 (-0.93, -0.21) 26.15
BMI<30
Madempudi (2019) -0.25 (-0.71, 0.20) 15.92
Firouzi (2017) -0.61 (-0.95, -0.27) 28.17
Tonucci (2015) -0.07 (-0.65, 0.52) 9.75
Hove (2015) -0.39 (-1.01, 0.24) 8.59
Wu (2015) -0.15 (-0.69, 0.39) 11.42
Subtotal (I-squared = 0.0%, p = 0.434) -0.36 (-0.58, -0.15) 73.85
Overall significance tests (P = 0.005)
NOTE: Weights are from random effects analysis
-1.11
0
1.11
C
Author(year)
SMD (95% CI)
Weight(%)
Eastern
Khalili (2019) -0.71 (-1.35, -0.07) 5.93
Madempudi (2019) -0.25 (-0.71, 0.20) 11.60
Sabico (2017) -0.51 (-0.96, -0.06) 11.93
Tajabadi-Ebrahimi (2016) -0.46 (-0.97, 0.06) 9.24
Firouzi (2017) -0.61 (-0.95, -0.27) 20.53
Asemi (2016) -0.63 (-1.03, -0.24) 15.34
Wu (2015) -0.15 (-0.69, 0.39) 8.32
Subtotal (I-squared = 0.0%, p = 0. -0.49 (-0.67, -0.32) 82.91
Western
Palacios (2020) -0.30 (-1.11, 0.51) 3.72
Tonucci (2015) -0.07 (-0.65, 0.52) 7.11
Hove (2015) -0.39 (-1.01, 0.24) 6.27
Subtotal (I-squared = 0.0%, p = 0.75 -0.23 (-0.61, 0.14) 17.09
Overall significance tests (P = 0.000)
NOTE: Weights are from random effects analysis
-1.35
0
1.35
D
Author(year)
SMD (95% CI)
Weight(%)
Weeks≤8
Khalili (2019) -0.71 (-1.35, -0.07) 5.93
Sabico (2017) -0.51 (-0.96, -0.06) 11.93
Tonucci (2015) -0.07 (-0.65, 0.52) 7.11
Asemi (2016) -0.63 (-1.03, -0.24) 15.34
Subtotal (I-squared = 0.0%, p = -0.51 (-0.75, -0.26) 40.31
8<Weeks ≤12
Palacios (2020) -0.30 (-1.11, 0.51) 3.72
Madempudi (2019) -0.25 (-0.71, 0.20) 11.60
Tajabadi-Ebrahimi (2016) -0.46 (-0.97, 0.06) 9.24
Firouzi (2017) -0.61 (-0.95, -0.27) 20.53
Hove (2015) -0.39 (-1.01, 0.24) 6.27
Subtotal (I-squared = 0.0%, p = 0. -0.45 (-0.67, -0.23) 51.36
12<Weeks ≤24
Wu (2015) -0.15 (-0.69, 0.39) 8.32
Subtotal (I-squared = .%, p = .) -0.15 (-0.69, 0.39) 8.32
Overall significance tests (P = 0.000)
NOTE: Weights are from random effects analysis
-1.35
0
1.35

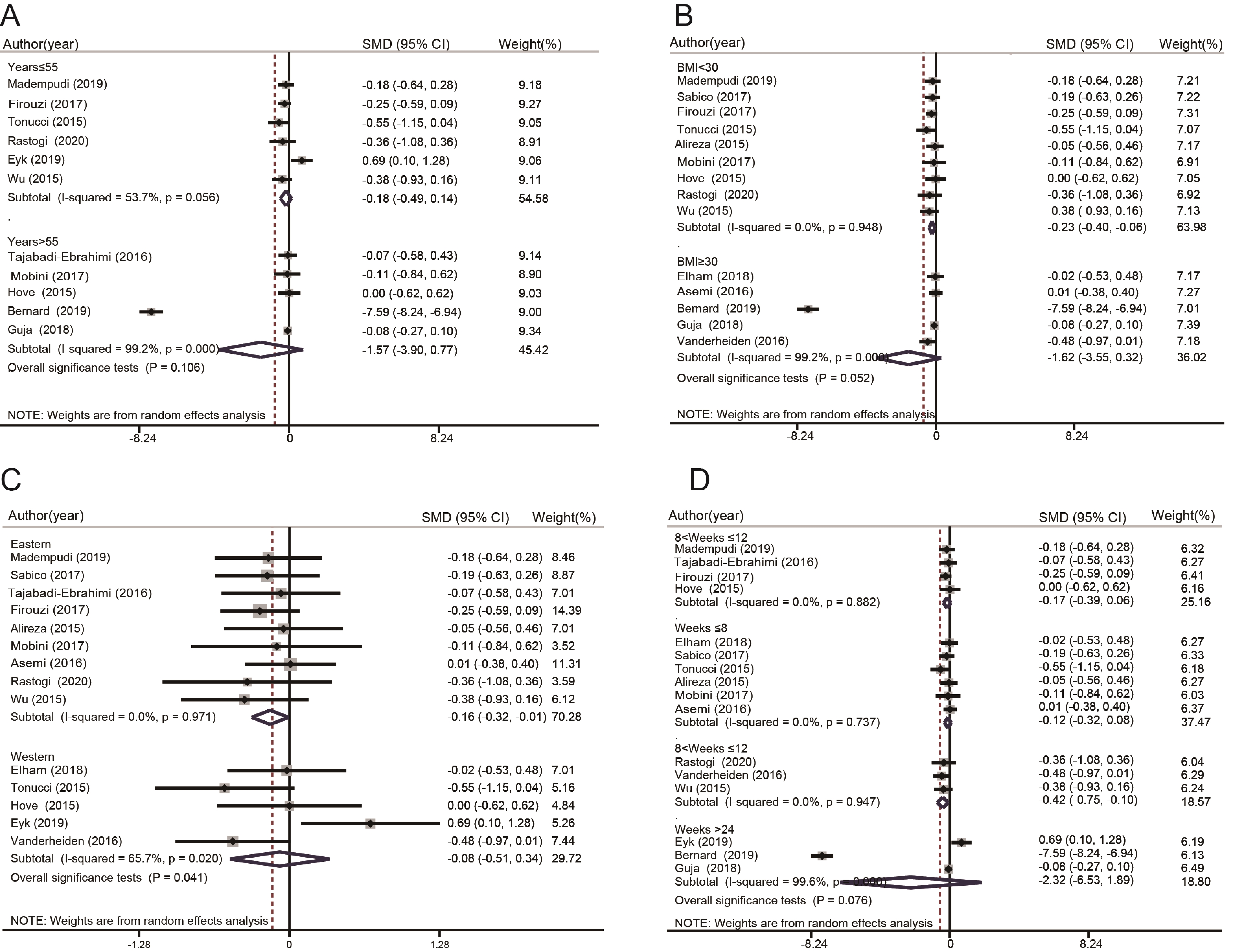

A
Author(year)
SMD (95% CI)
Weight(%)
Years≤55
Madempudi (2019) -0.18 (-0.64, 0.28) 9.18
Firouzi (2017) -0.25 (-0.59, 0.09) 9.27
Tonucci (2015) -0.55 (-1.15, 0.04) 9.05
Rastogi (2020) -0.36 (-1.08, 0.36) 8.91
Eyk (2019) 0.69 (0.10, 1.28) 9.06
Wu (2015) -0.38 (-0.93, 0.16) 9.11
Subtotal (I-squared = 53.7%, p = 0.056) -0.18 (-0.49, 0.14) 54.58
Years>55
Tajabadi-Ebrahimi (2016) -0.07 (-0.58, 0.43) 9.14
Mobini (2017) -0.11 (-0.84, 0.62) 8.90
Hove (2015) 0.00 (-0.62, 0.62) 9.03
Bernard (2019) -7.59 (-8.24, -6.94) 9.00
Guja (2018) -0.08 (-0.27, 0.10) 9.34
Subtotal (I-squared = 99.2%, p = 0.000) -1.57 (-3.90, 0.77) 45.42
Overall significance tests (P = 0.106)
NOTE: Weights are from random effects analysis
-8.24
0
8.24
B
Author(year)
SMD (95% CI)
Weight(%)
BMI<30
Madempudi (2019) -0.18 (-0.64, 0.28) 7.21
Sabico (2017) -0.19 (-0.63, 0.26) 7.22
Firouzi (2017) -0.25 (-0.59, 0.09) 7.31
Tonucci (2015) -0.55 (-1.15, 0.04) 7.07
Alireza (2015) -0.05 (-0.56, 0.46) 7.17
Mobini (2017) -0.11 (-0.84, 0.62) 6.91
Hove (2015) 0.00 (-0.62, 0.62) 7.05
Rastogi (2020) -0.36 (-1.08, 0.36) 6.92
Wu (2015) -0.38 (-0.93, 0.16) 7.13
Subtotal (I-squared = 0.0%, p = 0.948) -0.23 (-0.40, -0.06) 63.98
BMI≥30
Elham (2018) -0.02 (-0.53, 0.48) 7.17
Asemi (2016) 0.01 (-0.38, 0.40) 7.27
Bernard (2019) -7.59 (-8.24, -6.94) 7.01
Guja (2018) -0.08 (-0.27, 0.10) 7.39
Vanderheiden (2016) -0.48 (-0.97, 0.01) 7.18
Subtotal (I-squared = 99.2%, p = 0.000) -1.62 (-3.55, 0.32) 36.02
Overall significance tests (P = 0.052)
NOTE: Weights are from random effects analysis
-8.24
0
8.24
C
Author(year)
SMD (95% CI)
Weight(%)
Eastern
Madempudi (2019) -0.18 (-0.64, 0.28) 8.46
Sabico (2017) -0.19 (-0.63, 0.26) 8.87
Tajabadi-Ebrahimi (2016) -0.07 (-0.58, 0.43) 7.01
Firouzi (2017) -0.25 (-0.59, 0.09) 14.39
Alireza (2015) -0.05 (-0.56, 0.46) 7.01
Mobini (2017) -0.11 (-0.84, 0.62) 3.52
Asemi (2016) 0.01 (-0.38, 0.40) 11.31
Rastogi (2020) -0.36 (-1.08, 0.36) 3.59
Wu (2015) -0.38 (-0.93, 0.16) 6.12
Subtotal (I-squared = 0.0%, p = 0.971) -0.16 (-0.32, -0.01) 70.28
Western
Elham (2018) -0.02 (-0.53, 0.48) 7.01
Tonucci (2015) -0.55 (-1.15, 0.04) 5.16
Hove (2015) 0.00 (-0.62, 0.62) 4.84
Eyk (2019) 0.69 (0.10, 1.28) 5.26
Vanderheiden (2016) -0.48 (-0.97, 0.01) 7.44
Subtotal (I-squared = 65.7%, p = 0.020) -0.08 (-0.51, 0.34) 29.72
Overall significance tests (P = 0.041)
NOTE: Weights are from random effects analysis
-1.28
0
1.28
D
Author(year)
SMD (95% CI)
Weight(%)
8<Weeks ≤12
Madempudi (2019) -0.18 (-0.64, 0.28) 6.32
Tajabadi-Ebrahimi (2016) -0.07 (-0.58, 0.43) 6.27
Firouzi (2017) -0.25 (-0.59, 0.09) 6.41
Hove (2015) 0.00 (-0.62, 0.62) 6.16
Subtotal (I-squared = 0.0%, p = 0.882) -0.17 (-0.39, 0.06) 25.16
Weeks ≤8
Elham (2018) -0.02 (-0.53, 0.48) 6.27
Sabico (2017) -0.19 (-0.63, 0.26) 6.33
Tonucci (2015) -0.55 (-1.15, 0.04) 6.18
Alireza (2015) -0.05 (-0.56, 0.46) 6.27
Mobini (2017) -0.11 (-0.84, 0.62) 6.03
Asemi (2016) 0.01 (-0.38, 0.40) 6.37
Subtotal (I-squared = 0.0%, p = 0.737) -0.12 (-0.32, 0.08) 37.47
8<Weeks ≤12
Rastogi (2020) -0.36 (-1.08, 0.36) 6.04
Vanderheiden (2016) -0.48 (-0.97, 0.01) 6.29
Wu (2015) -0.38 (-0.93, 0.16) 6.24
Subtotal (I-squared = 0.0%, p = 0.947) -0.42 (-0.75, -0.10) 18.57
Weeks >24
Eyk (2019) 0.69 (0.10, 1.28) 6.19
Bernard (2019) -7.59 (-8.24, -6.94) 6.13
Guja (2018) -0.08 (-0.27, 0.10) 6.49
Subtotal (I-squared = 99.6%, p = 0.000) -2.32 (-6.53, 1.89) 18.80
Overall significance tests (P = 0.076)
NOTE: Weights are from random effects analysis
-8.24
0
8.24

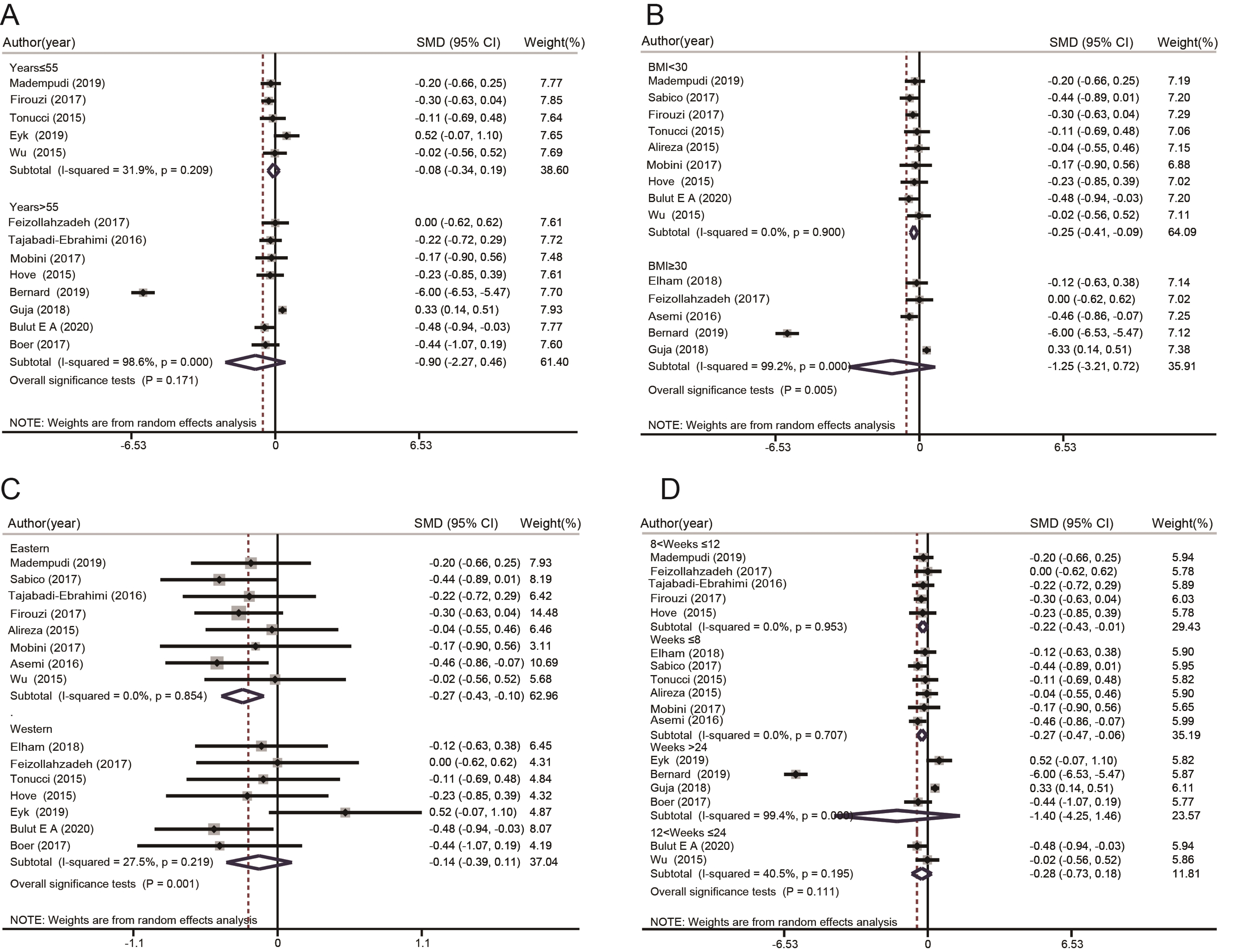
A
Author(year)
SMD (95% CI)
Weight(%)
Years≤55
Madempudi (2019) -0.20 (-0.66, 0.25) 7.77
Firouzi (2017) -0.30 (-0.63, 0.04) 7.85
Tonucci (2015) -0.11 (-0.69, 0.48) 7.64
Eyk (2019) 0.52 (-0.07, 1.10) 7.65
Wu (2015) -0.02 (-0.56, 0.52) 7.69
Subtotal (I-squared = 31.9%, p = 0.209) -0.08 (-0.34, 0.19) 38.60
Years>55
Feizollahzadeh (2017) 0.00 (-0.62, 0.62) 7.61
Tajabadi-Ebrahimi (2016) -0.22 (-0.72, 0.29) 7.72
Mobini (2017) -0.17 (-0.90, 0.56) 7.48
Hove (2015) -0.23 (-0.85, 0.39) 7.61
Bernard (2019) -6.00 (-6.53, -5.47) 7.70
Guja (2018) 0.33 (0.14, 0.51) 7.93
Bulut E A (2020) -0.48 (-0.94, -0.03) 7.77
Boer (2017) -0.44 (-1.07, 0.19) 7.60
Subtotal (I-squared = 98.6%, p = 0.000) -0.90 (-2.27, 0.46) 61.40
Overall significance tests (P = 0.171)
NOTE: Weights are from random effects analysis
-6.53
0
6.53
B
Author(year)
SMD (95% CI)
Weight(%)
BMI<30
Madempudi (2019) -0.20 (-0.66, 0.25) 7.19
Sabico (2017) -0.44 (-0.89, 0.01) 7.20
Firouzi (2017) -0.30 (-0.63, 0.04) 7.29
Tonucci (2015) -0.11 (-0.69, 0.48) 7.06
Alireza (2015) -0.04 (-0.55, 0.46) 7.15
Mobini (2017) -0.17 (-0.90, 0.56) 6.88
Hove (2015) -0.23 (-0.85, 0.39) 7.02
Bulut E A (2020) -0.48 (-0.94, -0.03) 7.20
Wu (2015) -0.02 (-0.56, 0.52) 7.11
Subtotal (I-squared = 0.0%, p = 0.900) -0.25 (-0.41, -0.09) 64.09
BMI≥30
Elham (2018) -0.12 (-0.63, 0.38) 7.14
Feizollahzadeh (2017) 0.00 (-0.62, 0.62) 7.02
Asemi (2016) -0.46 (-0.86, -0.07) 7.25
Bernard (2019) -6.00 (-6.53, -5.47) 7.12
Guja (2018) 0.33 (0.14, 0.51) 7.38
Subtotal (I-squared = 99.2%, p = 0.000) -1.25 (-3.21, 0.72) 35.91
Overall significance tests (P = 0.005)
NOTE: Weights are from random effects analysis
-6.53
0
6.53
C
Author(year)
SMD (95% CI)
Weight(%)
Eastern
Madempudi (2019) -0.20 (-0.66, 0.25) 7.93
Sabico (2017) -0.44 (-0.89, 0.01) 8.19
Tajabadi-Ebrahimi (2016) -0.22 (-0.72, 0.29) 6.42
Firouzi (2017) -0.30 (-0.63, 0.04) 14.48
Alireza (2015) -0.04 (-0.55, 0.46) 6.46
Mobini (2017) -0.17 (-0.90, 0.56) 3.11
Asemi (2016) -0.46 (-0.86, -0.07) 10.69
Wu (2015) -0.02 (-0.56, 0.52) 5.68
Subtotal (I-squared = 0.0%, p = 0.854) -0.27 (-0.43, -0.10) 62.96
Western
Elham (2018) -0.12 (-0.63, 0.38) 6.45
Feizollahzadeh (2017) 0.00 (-0.62, 0.62) 4.31
Tonucci (2015) -0.11 (-0.69, 0.48) 4.84
Hove (2015) -0.23 (-0.85, 0.39) 4.32
Eyk (2019) 0.52 (-0.07, 1.10) 4.87
Bulut E A (2020) -0.48 (-0.94, -0.03) 8.07
Boer (2017) -0.44 (-1.07, 0.19) 4.19
Subtotal (I-squared = 27.5%, p = 0.219) -0.14 (-0.39, 0.11) 37.04
Overall significance tests (P = 0.001)
NOTE: Weights are from random effects analysis
-1.1
0
1.1
D
Author(year)
SMD (95% CI)
Weight(%)
8<Weeks ≤12
Madempudi (2019) -0.20 (-0.66, 0.25) 5.94
Feizollahzadeh (2017) 0.00 (-0.62, 0.62) 5.78
Tajabadi-Ebrahimi (2016) -0.22 (-0.72, 0.29) 5.89
Firouzi (2017) -0.30 (-0.63, 0.04) 6.03
Hove (2015) -0.23 (-0.85, 0.39) 5.78
Subtotal (I-squared = 0.0%, p = 0.953) -0.22 (-0.43, -0.01) 29.43
Weeks ≤8
Elham (2018) -0.12 (-0.63, 0.38) 5.90
Sabico (2017) -0.44 (-0.89, 0.01) 5.95
Tonucci (2015) -0.11 (-0.69, 0.48) 5.82
Alireza (2015) -0.04 (-0.55, 0.46) 5.90
Mobini (2017) -0.17 (-0.90, 0.56) 5.65
Asemi (2016) -0.46 (-0.86, -0.07) 5.99
Subtotal (I-squared = 0.0%, p = 0.707) -0.27 (-0.47, -0.06) 35.19
Weeks >24
Eyk (2019) 0.52 (-0.07, 1.10) 5.82
Bernard (2019) -6.00 (-6.53, -5.47) 5.87
Guja (2018) 0.33 (0.14, 0.51) 6.11
Boer (2017) -0.44 (-1.07, 0.19) 5.77
Subtotal (I-squared = 99.4%, p = 0.000) -1.40 (-4.25, 1.46) 23.57
12<Weeks ≤24
Bulut E A (2020) -0.48 (-0.94, -0.03) 5.94
Wu (2015) -0.02 (-0.56, 0.52) 5.86
Subtotal (I-squared = 40.5%, p = 0.195) -0.28 (-0.73, 0.18) 11.81
Overall significance tests (P = 0.111)
NOTE: Weights are from random effects analysis
-6.53
0
6.53

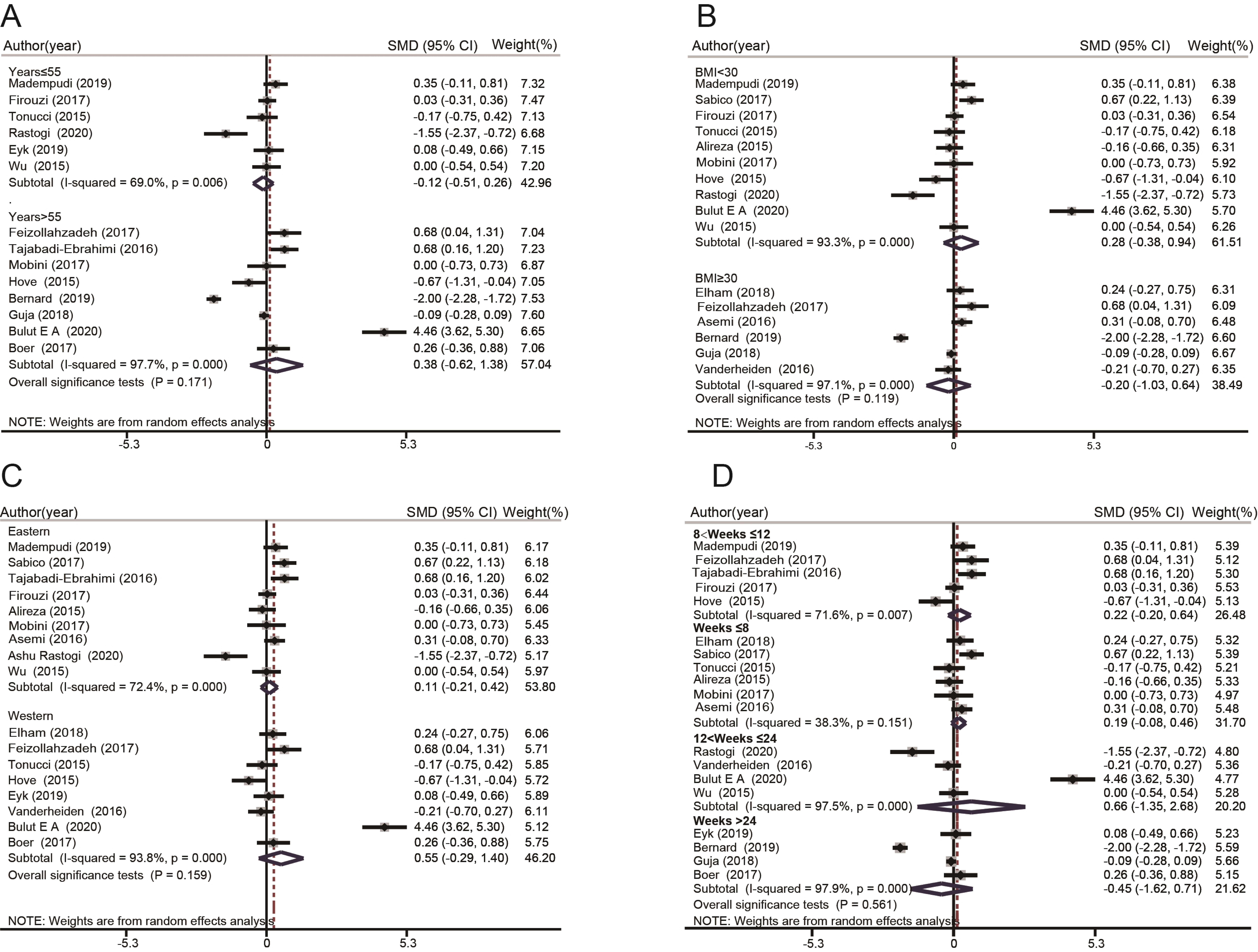

A
Author(year) SMD (95% CI) Weight(%)
Years≤55
Madempudi (2019) 0.35 (-0.11, 0.81) 7.32
Firouzi (2017) 0.03 (-0.31, 0.36) 7.47
Tonucci (2015) -0.17 (-0.75, 0.42) 7.13
Rastogi (2020) -1.55 (-2.37, -0.72) 6.68
Eyk (2019) 0.08 (-0.49, 0.66) 7.15
Wu (2015) 0.00 (-0.54, 0.54) 7.20
Subtotal (I-squared = 69.0%, p = 0.006) -0.12 (-0.51, 0.26) 42.96
Years>55
Feizollahzadeh (2017) 0.68 (0.04, 1.31) 7.04
Tajabadi-Ebrahimi (2016) 0.68 (0.16, 1.20) 7.23
Mobini (2017) 0.00 (-0.73, 0.73) 6.87
Hove (2015) -0.67 (-1.31, -0.04) 7.05
Bernard (2019) -2.00 (-2.28, -1.72) 7.53
Guja (2018) -0.09 (-0.28, 0.09) 7.60
Bulut E A (2020) 4.46 (3.62, 5.30) 6.65
Boer (2017) 0.26 (-0.36, 0.88) 7.06
Subtotal (I-squared = 97.7%, p = 0.000) 0.38 (-0.62, 1.38) 57.04
Overall significance tests (P = 0.171)
NOTE: Weights are from random effects analysis
-5.3
0
5.3
B
Author(year) SMD (95% CI) Weight(%)
BMI<30
Madempudi (2019) 0.35 (-0.11, 0.81) 6.38
Sabico (2017) 0.67 (0.22, 1.13) 6.39
Firouzi (2017) 0.03 (-0.31, 0.36) 6.54
Tonucci (2015) -0.17 (-0.75, 0.42) 6.18
Alireza (2015) -0.16 (-0.66, 0.35) 6.31
Mobini (2017) 0.00 (-0.73, 0.73) 5.92
Hove (2015) -0.67 (-1.31, -0.04) 6.10
Rastogi (2020) -1.55 (-2.37, -0.72) 5.73
Bulut E A (2020) 4.46 (3.62, 5.30) 5.70
Wu (2015) 0.00 (-0.54, 0.54) 6.26
Subtotal (I-squared = 93.3%, p = 0.000) 0.28 (-0.38, 0.94) 61.51
BMI≥30
Elham (2018) 0.24 (-0.27, 0.75) 6.31
Feizollahzadeh (2017) 0.68 (0.04, 1.31) 6.09
Asemi (2016) 0.31 (-0.08, 0.70) 6.48
Bernard (2019) -2.00 (-2.28, -1.72) 6.60
Guja (2018) -0.09 (-0.28, 0.09) 6.67
Vanderheiden (2016) -0.21 (-0.70, 0.27) 6.35
Subtotal (I-squared = 97.1%, p = 0.000) -0.20 (-1.03, 0.64) 38.49
Overall significance tests (P = 0.119)
NOTE: Weights are from random effects analysis
-5.3
0
5.3
C
Author(year) SMD (95% CI) Weight(%)
Eastern
Madempudi (2019) 0.35 (-0.11, 0.81) 6.17
Sabico (2017) 0.67 (0.22, 1.13) 6.18
Tajabadi-Ebrahimi (2016) 0.68 (0.16, 1.20) 6.02
Firouzi (2017) 0.03 (-0.31, 0.36) 6.44
Alireza (2015) -0.16 (-0.66, 0.35) 6.06
Mobini (2017) 0.00 (-0.73, 0.73) 5.45
Asemi (2016) 0.31 (-0.08, 0.70) 6.33
Ashu Rastogi (2020) -1.55 (-2.37, -0.72) 5.17
Wu (2015) 0.00 (-0.54, 0.54) 5.97
Subtotal (I-squared = 72.4%, p = 0.000) 0.11 (-0.21, 0.42) 53.80
Western
Elham (2018) 0.24 (-0.27, 0.75) 6.06
Feizollahzadeh (2017) 0.68 (0.04, 1.31) 5.71
Tonucci (2015) -0.17 (-0.75, 0.42) 5.85
Hove (2015) -0.67 (-1.31, -0.04) 5.72
Eyk (2019) 0.08 (-0.49, 0.66) 5.89
Vanderheiden (2016) -0.21 (-0.70, 0.27) 6.11
Bulut E A (2020) 4.46 (3.62, 5.30) 5.12
Boer (2017) 0.26 (-0.36, 0.88) 5.75
Subtotal (I-squared = 93.8%, p = 0.000) 0.55 (-0.29, 1.40) 46.20
Overall significance tests (P = 0.159)
NOTE: Weights are from random effects analysis
-5.3
0
5.3
D
Author(year) SMD (95% CI) Weight(%)
8<Weeks ≤12
Madempudi (2019) 0.35 (-0.11, 0.81) 5.39
Feizollahzadeh (2017) 0.68 (0.04, 1.31) 5.12
Tajabadi-Ebrahimi (2016) 0.68 (0.16, 1.20) 5.30
Firouzi (2017) 0.03 (-0.31, 0.36) 5.53
Hove (2015) -0.67 (-1.31, -0.04) 5.13
Subtotal (I-squared = 71.6%, p = 0.007) 0.22 (-0.20, 0.64) 26.48
Weeks ≤8
Elham (2018) 0.24 (-0.27, 0.75) 5.32
Sabico (2017) 0.67 (0.22, 1.13) 5.39
Tonucci (2015) -0.17 (-0.75, 0.42) 5.21
Alireza (2015) -0.16 (-0.66, 0.35) 5.33
Mobini (2017) 0.00 (-0.73, 0.73) 4.97
Asemi (2016) 0.31 (-0.08, 0.70) 5.48
Subtotal (I-squared = 38.3%, p = 0.151) 0.19 (-0.08, 0.46) 31.70
12<Weeks ≤24
Rastogi (2020) -1.55 (-2.37, -0.72) 4.80
Vanderheiden (2016) -0.21 (-0.70, 0.27) 5.36
Bulut E A (2020) 4.46 (3.62, 5.30) 4.77
Wu (2015) 0.00 (-0.54, 0.54) 5.28
Subtotal (I-squared = 97.5%, p = 0.000) 0.66 (-1.35, 2.68) 20.20
Weeks >24
Eyk (2019) 0.08 (-0.49, 0.66) 5.23
Bernard (2019) -2.00 (-2.28, -1.72) 5.59
Guja (2018) -0.09 (-0.28, 0.09) 5.66
Boer (2017) 0.26 (-0.36, 0.88) 5.15
Subtotal (I-squared = 97.9%, p = 0.000) -0.45 (-1.62, 0.71) 21.62
Overall significance tests (P = 0.561)
NOTE: Weights are from random effects analysis
-5.3
0
5.3

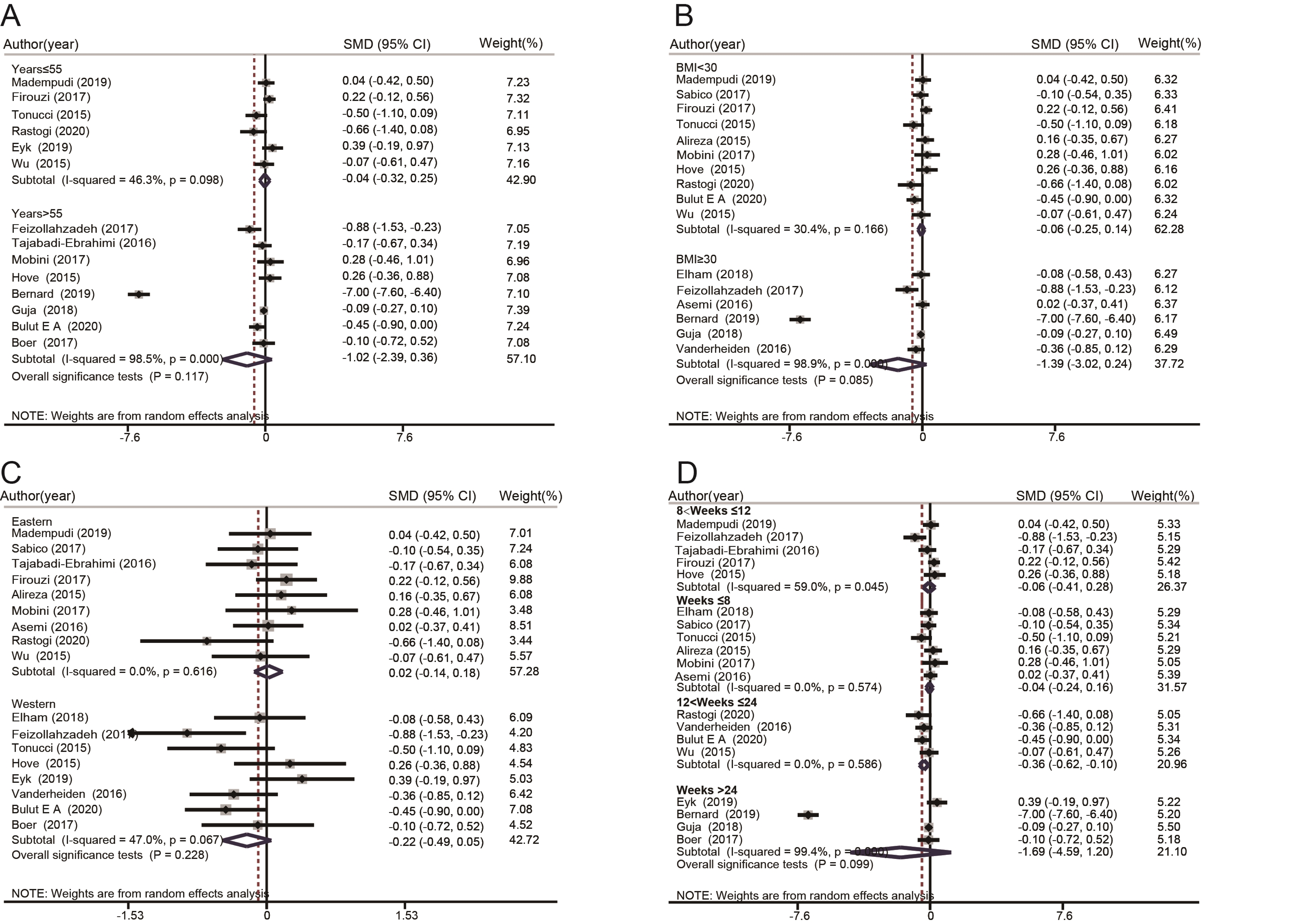

A
Author(year)
SMD (95% CI)
Weight(%)
Years≤55
Madempudi (2019) 0.04 (-0.42, 0.50) 7.23
Firouzi (2017) 0.22 (-0.12, 0.56) 7.32
Tonucci (2015) -0.50 (-1.10, 0.09) 7.11
Rastogi (2020) -0.66 (-1.40, 0.08) 6.95
Eyk (2019) 0.39 (-0.19, 0.97) 7.13
Wu (2015) -0.07 (-0.61, 0.47) 7.16
Subtotal (I-squared = 46.3%, p = 0.098) -0.04 (-0.32, 0.25) 42.90
Years>55
Feizollahzadeh (2017) -0.88 (-1.53, -0.23) 7.05
Tajabadi-Ebrahimi (2016) -0.17 (-0.67, 0.34) 7.19
Mobini (2017) 0.28 (-0.46, 1.01) 6.96
Hove (2015) 0.26 (-0.36, 0.88) 7.08
Bernard (2019) -7.00 (-7.60, -6.40) 7.10
Guja (2018) -0.09 (-0.27, 0.10) 7.39
Bulut E A (2020) -0.45 (-0.90, 0.00) 7.24
Boer (2017) -0.10 (-0.72, 0.52) 7.08
Subtotal (I-squared = 98.5%, p = 0.000) -1.02 (-2.39, 0.36) 57.10
Overall significance tests (P = 0.117)
NOTE: Weights are from random effects analysis
-7.6
0
7.6
B
Author(year)
SMD (95% CI)
Weight(%)
BMI<30
Madempudi (2019) 0.04 (-0.42, 0.50) 6.32
Sabico (2017) -0.10 (-0.54, 0.35) 6.33
Firouzi (2017) 0.22 (-0.12, 0.56) 6.41
Tonucci (2015) -0.50 (-1.10, 0.09) 6.18
Alireza (2015) 0.16 (-0.35, 0.67) 6.27
Mobini (2017) 0.28 (-0.46, 1.01) 6.02
Hove (2015) 0.26 (-0.36, 0.88) 6.16
Rastogi (2020) -0.66 (-1.40, 0.08) 6.02
Bulut E A (2020) -0.45 (-0.90, 0.00) 6.32
Wu (2015) -0.07 (-0.61, 0.47) 6.24
Subtotal (I-squared = 30.4%, p = 0.166) -0.06 (-0.25, 0.14) 62.28
BMI≥30
Elham (2018) -0.08 (-0.58, 0.43) 6.27
Feizollahzadeh (2017) -0.88 (-1.53, -0.23) 6.12
Asemi (2016) 0.02 (-0.37, 0.41) 6.37
Bernard (2019) -7.00 (-7.60, -6.40) 6.17
Guja (2018) -0.09 (-0.27, 0.10) 6.49
Vanderheiden (2016) -0.36 (-0.85, 0.12) 6.29
Subtotal (I-squared = 98.9%, p = 0.000) -1.39 (-3.02, 0.24) 37.72
Overall significance tests (P = 0.085)
NOTE: Weights are from random effects analysis
-7.6
0
7.6
C
Author(year)
SMD (95% CI)
Weight(%)
Eastern
Madempudi (2019) 0.04 (-0.42, 0.50) 7.01
Sabico (2017) -0.10 (-0.54, 0.35) 7.24
Tajabadi-Ebrahimi (2016) -0.17 (-0.67, 0.34) 6.08
Firouzi (2017) 0.22 (-0.12, 0.56) 9.88
Alireza (2015) 0.16 (-0.35, 0.67) 6.08
Mobini (2017) 0.28 (-0.46, 1.01) 3.48
Asemi (2016) 0.02 (-0.37, 0.41) 8.51
Rastogi (2020) -0.66 (-1.40, 0.08) 3.44
Wu (2015) -0.07 (-0.61, 0.47) 5.57
Subtotal (I-squared = 0.0%, p = 0.616) 0.02 (-0.14, 0.18) 57.28
Western
Elham (2018) -0.08 (-0.58, 0.43) 6.09
Feizollahzadeh (2017) -0.88 (-1.53, -0.23) 4.20
Tonucci (2015) -0.50 (-1.10, 0.09) 4.83
Hove (2015) 0.26 (-0.36, 0.88) 4.54
Eyk (2019) 0.39 (-0.19, 0.97) 5.03
Vanderheiden (2016) -0.36 (-0.85, 0.12) 6.42
Bulut E A (2020) -0.45 (-0.90, 0.00) 7.08
Boer (2017) -0.10 (-0.72, 0.52) 4.52
Subtotal (I-squared = 47.0%, p = 0.067) -0.22 (-0.49, 0.05) 42.72
Overall significance tests (P = 0.228)
NOTE: Weights are from random effects analysis
-1.53
0
1.53
D
Author(year)
SMD (95% CI)
Weight(%)
8<Weeks ≤12
Madempudi (2019) 0.04 (-0.42, 0.50) 5.33
Feizollahzadeh (2017) -0.88 (-1.53, -0.23) 5.15
Tajabadi-Ebrahimi (2016) -0.17 (-0.67, 0.34) 5.29
Firouzi (2017) 0.22 (-0.12, 0.56) 5.42
Hove (2015) 0.26 (-0.36, 0.88) 5.18
Subtotal (I-squared = 59.0%, p = 0.045) -0.06 (-0.41, 0.28) 26.37
Weeks ≤8
Elham (2018) -0.08 (-0.58, 0.43) 5.29
Sabico (2017) -0.10 (-0.54, 0.35) 5.34
Tonucci (2015) -0.50 (-1.10, 0.09) 5.21
Alireza (2015) 0.16 (-0.35, 0.67) 5.29
Mobini (2017) 0.28 (-0.46, 1.01) 5.05
Asemi (2016) 0.02 (-0.37, 0.41) 5.39
Subtotal (I-squared = 0.0%, p = 0.574) -0.04 (-0.24, 0.16) 31.57
12<Weeks ≤24
Rastogi (2020) -0.66 (-1.40, 0.08) 5.05
Vanderheiden (2016) -0.36 (-0.85, 0.12) 5.31
Bulut E A (2020) -0.45 (-0.90, 0.00) 5.34
Wu (2015) -0.07 (-0.61, 0.47) 5.26
Subtotal (I-squared = 0.0%, p = 0.586) -0.36 (-0.62, -0.10) 20.96
Weeks >24
Eyk (2019) 0.39 (-0.19, 0.97) 5.22
Bernard (2019) -7.00 (-7.60, -6.40) 5.20
Guja (2018) -0.09 (-0.27, 0.10) 5.50
Boer (2017) -0.10 (-0.72, 0.52) 5.18
Subtotal (I-squared = 99.4%, p = 0.000) -1.69 (-4.59, 1.20) 21.10
Overall significance tests (P = 0.099)
NOTE: Weights are from random effects analysis
-7.6
0
7.6

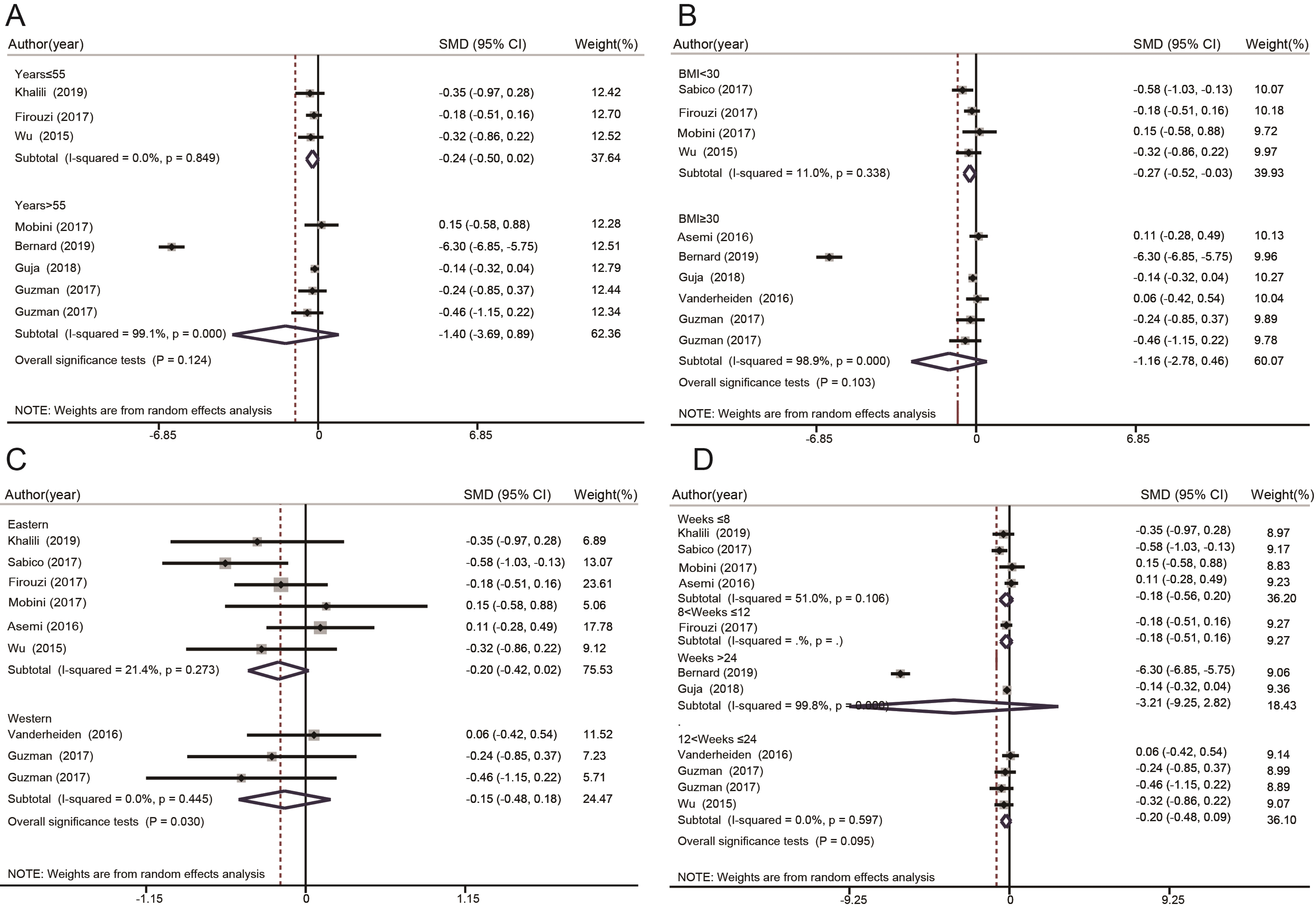
A
Author(year)
SMD (95% CI)
Weight(%)
Years≤55
Khalili (2019) -0.35 (-0.97, 0.28) 12.42
Firouzi (2017) -0.18 (-0.51, 0.16) 12.70
Wu (2015) -0.32 (-0.86, 0.22) 12.52
Subtotal (I-squared = 0.0%, p = 0.849) -0.24 (-0.50, 0.02) 37.64
Years>55
Mobini (2017) 0.15 (-0.58, 0.88) 12.28
Bernard (2019) -6.30 (-6.85, -5.75) 12.51
Guja (2018) -0.14 (-0.32, 0.04) 12.79
Guzman (2017) -0.24 (-0.85, 0.37) 12.44
Guzman (2017) -0.46 (-1.15, 0.22) 12.34
Subtotal (I-squared = 99.1%, p = 0.000) -1.40 (-3.69, 0.89) 62.36
Overall significance tests (P = 0.124)
NOTE: Weights are from random effects analysis
-6.85
0
6.85
B
Author(year)
SMD (95% CI)
Weight(%)
BMI<30
Sabico (2017) -0.58 (-1.03, -0.13) 10.07
Firouzi (2017) -0.18 (-0.51, 0.16) 10.18
Mobini (2017) 0.15 (-0.58, 0.88) 9.72
Wu (2015) -0.32 (-0.86, 0.22) 9.97
Subtotal (I-squared = 11.0%, p = 0.338) -0.27 (-0.52, -0.03) 39.93
BMI≥30
Asemi (2016) 0.11 (-0.28, 0.49) 10.13
Bernard (2019) -6.30 (-6.85, -5.75) 9.96
Guja (2018) -0.14 (-0.32, 0.04) 10.27
Vanderheiden (2016) 0.06 (-0.42, 0.54) 10.04
Guzman (2017) -0.24 (-0.85, 0.37) 9.89
Guzman (2017) -0.46 (-1.15, 0.22) 9.78
Subtotal (I-squared = 98.9%, p = 0.000) -1.16 (-2.78, 0.46) 60.07
Overall significance tests (P = 0.103)
NOTE: Weights are from random effects analysis
-6.85
0
6.85
C
Author(year)
SMD (95% CI)
Weight(%)
Eastern
Khalili (2019) -0.35 (-0.97, 0.28) 6.89
Sabico (2017) -0.58 (-1.03, -0.13) 13.07
Firouzi (2017) -0.18 (-0.51, 0.16) 23.61
Mobini (2017) 0.15 (-0.58, 0.88) 5.06
Asemi (2016) 0.11 (-0.28, 0.49) 17.78
Wu (2015) -0.32 (-0.86, 0.22) 9.12
Subtotal (I-squared = 21.4%, p = 0.273) -0.20 (-0.42, 0.02) 75.53
Western
Vanderheiden (2016) 0.06 (-0.42, 0.54) 11.52
Guzman (2017) -0.24 (-0.85, 0.37) 7.23
Guzman (2017) -0.46 (-1.15, 0.22) 5.71
Subtotal (I-squared = 0.0%, p = 0.445) -0.15 (-0.48, 0.18) 24.47
Overall significance tests (P = 0.030)
NOTE: Weights are from random effects analysis
-1.15
0
1.15
D
Author(year)
SMD (95% CI)
Weight(%)
Weeks ≤8
Khalili (2019) -0.35 (-0.97, 0.28) 8.97
Sabico (2017) -0.58 (-1.03, -0.13) 9.17
Mobini (2017) 0.15 (-0.58, 0.88) 8.83
Asemi (2016) 0.11 (-0.28, 0.49) 9.23
Subtotal (I-squared = 51.0%, p = 0.106) -0.18 (-0.56, 0.20) 36.20
8<Weeks ≤12
Firouzi (2017) -0.18 (-0.51, 0.16) 9.27
Subtotal (I-squared = .%, p = .) -0.18 (-0.51, 0.16) 9.27
Weeks >24
Bernard (2019) -6.30 (-6.85, -5.75) 9.06
Guja (2018) -0.14 (-0.32, 0.04) 9.36
Subtotal (I-squared = 99.8%, p = 0.000) -3.21 (-9.25, 2.82) 18.43
12<Weeks ≤24
Vanderheiden (2016) 0.06 (-0.42, 0.54) 9.14
Guzman (2017) -0.24 (-0.85, 0.37) 8.99
Guzman (2017) -0.46 (-1.15, 0.22) 8.89
Wu (2015) -0.32 (-0.86, 0.22) 9.07
Subtotal (I-squared = 0.0%, p = 0.597) -0.20 (-0.48, 0.09) 36.10
Overall significance tests (P = 0.095)
NOTE: Weights are from random effects analysis
-9.25
0
9.25

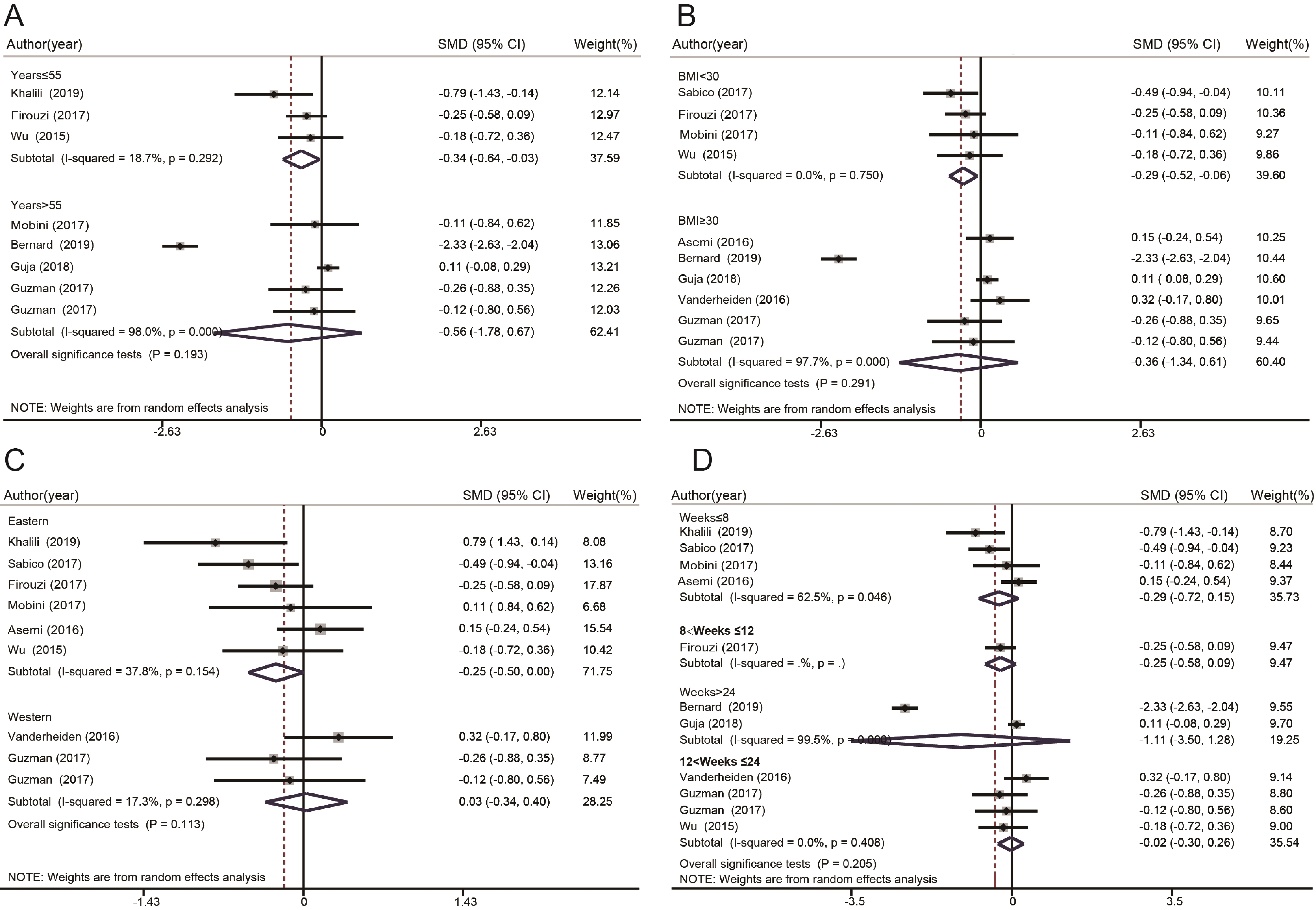
A
Author(year)
SMD (95% CI)
Weight(%)
Years≤55
Khalili (2019) -0.79 (-1.43, -0.14) 12.14
Firouzi (2017) -0.25 (-0.58, 0.09) 12.97
Wu (2015) -0.18 (-0.72, 0.36) 12.47
Subtotal (I-squared = 18.7%, p = 0.292) -0.34 (-0.64, -0.03) 37.59
Years>55
Mobini (2017) -0.11 (-0.84, 0.62) 11.85
Bernard (2019) -2.33 (-2.63, -2.04) 13.06
Guja (2018) 0.11 (-0.08, 0.29) 13.21
Guzman (2017) -0.26 (-0.88, 0.35) 12.26
Guzman (2017) -0.12 (-0.80, 0.56) 12.03
Subtotal (I-squared = 98.0%, p = 0.000) -0.56 (-1.78, 0.67) 62.41
Overall significance tests (P = 0.193)
NOTE: Weights are from random effects analysis
-2.63
0
2.63
B
Author(year)
SMD (95% CI)
Weight(%)
BMI<30
Sabico (2017) -0.49 (-0.94, -0.04) 10.11
Firouzi (2017) -0.25 (-0.58, 0.09) 10.36
Mobini (2017) -0.11 (-0.84, 0.62) 9.27
Wu (2015) -0.18 (-0.72, 0.36) 9.86
Subtotal (I-squared = 0.0%, p = 0.750) -0.29 (-0.52, -0.06) 39.60
BMI≥30
Asemi (2016) 0.15 (-0.24, 0.54) 10.25
Bernard (2019) -2.33 (-2.63, -2.04) 10.44
Guja (2018) 0.11 (-0.08, 0.29) 10.60
Vanderheiden (2016) 0.32 (-0.17, 0.80) 10.01
Guzman (2017) -0.26 (-0.88, 0.35) 9.65
Guzman (2017) -0.12 (-0.80, 0.56) 9.44
Subtotal (I-squared = 97.7%, p = 0.000) -0.36 (-1.34, 0.61) 60.40
Overall significance tests (P = 0.291)
NOTE: Weights are from random effects analysis
-2.63
0
2.63
C
Author(year)
SMD (95% CI)
Weight(%)
Eastern
Khalili (2019) -0.79 (-1.43, -0.14) 8.08
Sabico (2017) -0.49 (-0.94, -0.04) 13.16
Firouzi (2017) -0.25 (-0.58, 0.09) 17.87
Mobini (2017) -0.11 (-0.84, 0.62) 6.68
Asemi (2016) 0.15 (-0.24, 0.54) 15.54
Wu (2015) -0.18 (-0.72, 0.36) 10.42
Subtotal (I-squared = 37.8%, p = 0.154) -0.25 (-0.50, 0.00) 71.75
Western
Vanderheiden (2016) 0.32 (-0.17, 0.80) 11.99
Guzman (2017) -0.26 (-0.88, 0.35) 8.77
Guzman (2017) -0.12 (-0.80, 0.56) 7.49
Subtotal (I-squared = 17.3%, p = 0.298) 0.03 (-0.34, 0.40) 28.25
Overall significance tests (P = 0.113)
NOTE: Weights are from random effects analysis
-1.43
0
1.43
D
Author(year)
SMD (95% CI)
Weight(%)
Weeks≤8
Khalili (2019) -0.79 (-1.43, -0.14) 8.70
Sabico (2017) -0.49 (-0.94, -0.04) 9.23
Mobini (2017) -0.11 (-0.84, 0.62) 8.44
Asemi (2016) 0.15 (-0.24, 0.54) 9.37
Subtotal (I-squared = 62.5%, p = 0.046) -0.29 (-0.72, 0.15) 35.73
8<Weeks ≤12
Firouzi (2017) -0.25 (-0.58, 0.09) 9.47
Subtotal (I-squared = .%, p = .) -0.25 (-0.58, 0.09) 9.47
Weeks>24
Bernard (2019) -2.33 (-2.63, -2.04) 9.55
Guja (2018) 0.11 (-0.08, 0.29) 9.70
Subtotal (I-squared = 99.5%, p = 0.000) -1.11 (-3.50, 1.28) 19.25
12<Weeks ≤24
Vanderheiden (2016) 0.32 (-0.17, 0.80) 9.14
Guzman (2017) -0.26 (-0.88, 0.35) 8.80
Guzman (2017) -0.12 (-0.80, 0.56) 8.60
Wu (2015) -0.18 (-0.72, 0.36) 9.00
Subtotal (I-squared = 0.0%, p = 0.408) -0.02 (-0.30, 0.26) 35.54
Overall significance tests (P = 0.205)
NOTE: Weights are from random effects analysis
-3.5
0
3.5

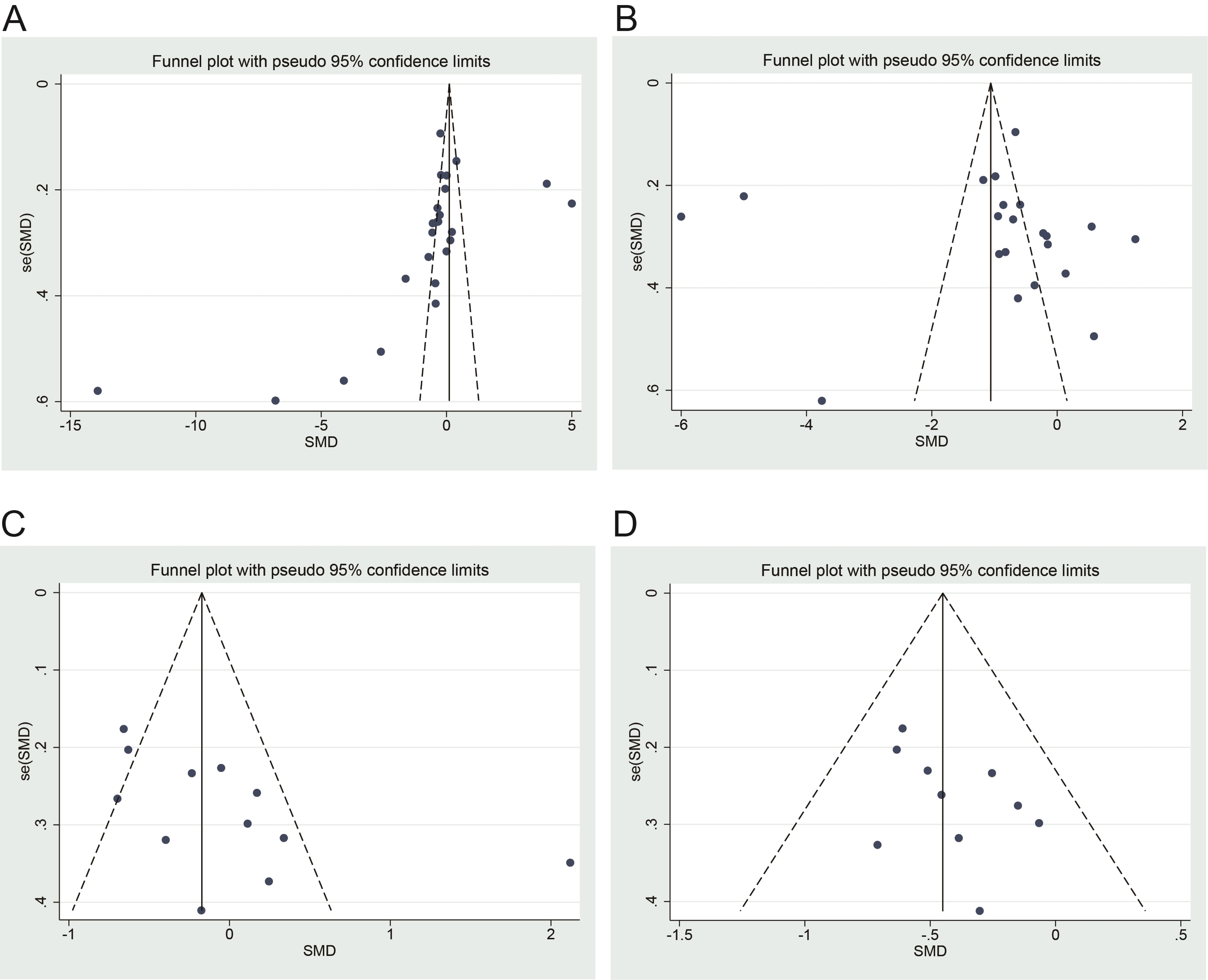
A
Funnel plot with pseudo 95% confidence limits
se(SMD)
SMD
B
Funnel plot with pseudo 95% confidence limits
se(SMD)
SMD
C
Funnel plot with pseudo 95% confidence limits
se(SMD)
SMD
D
Funnel plot with pseudo 95% confidence limits
se(SMD)
SMD

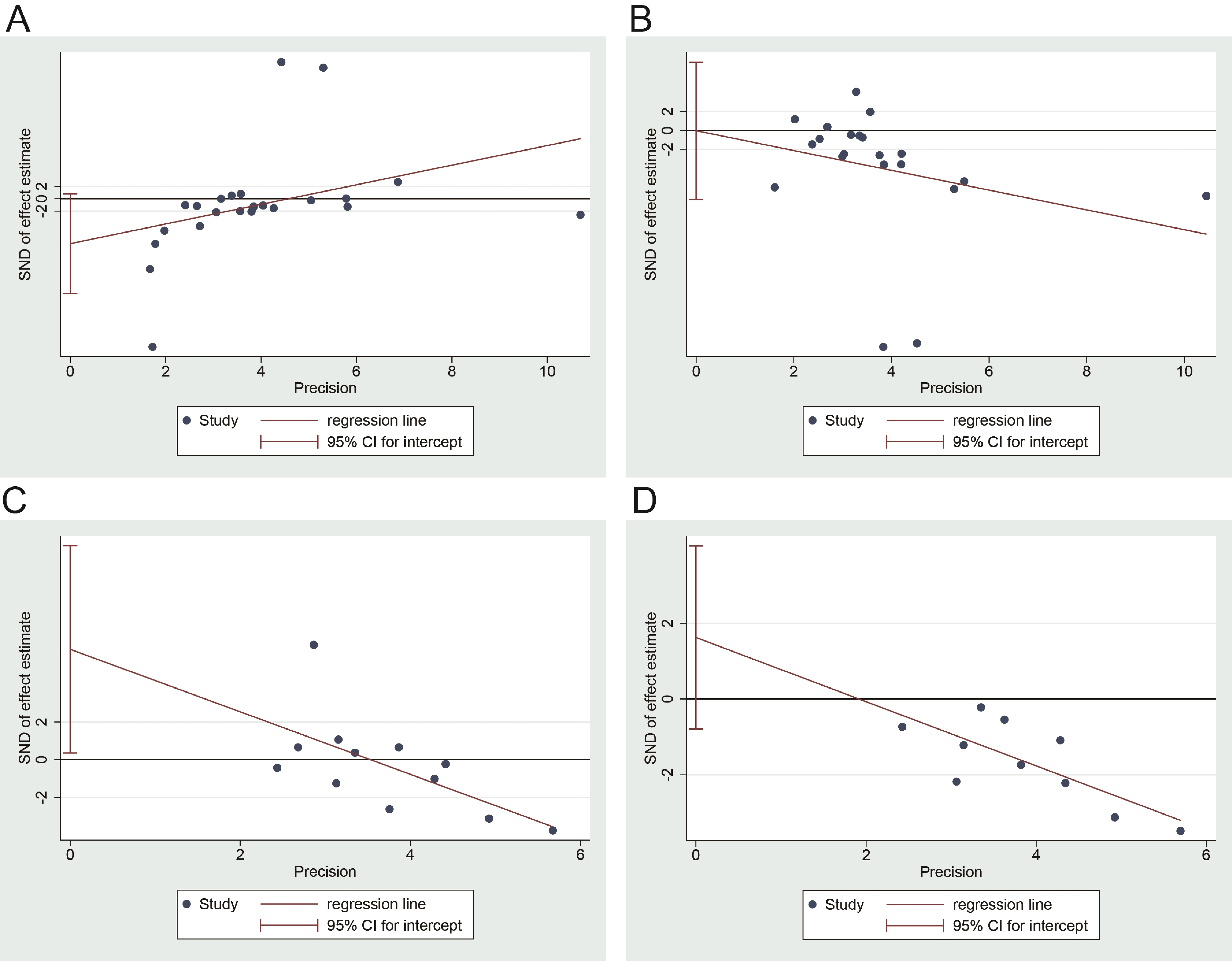

Note: A_metabias_ES_seES, egger(p=0.075); B_metabias_ES_seES, egger(p=0.991)
C_metabias_ES_seES, egger(p=0.039); D_metabias_ES_seES, egger(p=0.160)

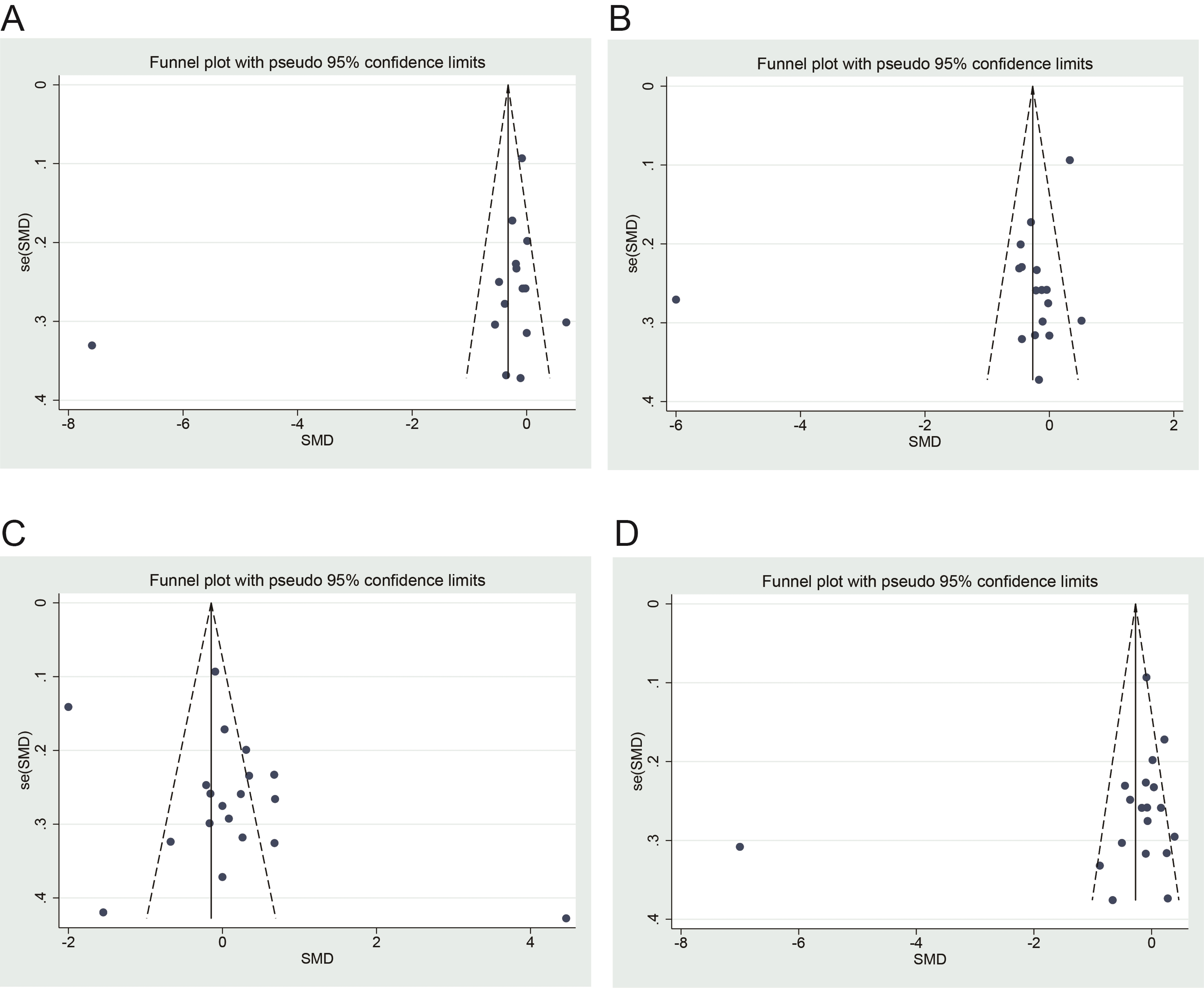
A
Funnel plot with pseudo 95% confidence limits
se(SMD)
0
.1
.2
.3
.4
-8
-6
-4
-2
0
SMD
B
Funnel plot with pseudo 95% confidence limits
se(SMD)
0
.1
.2
.3
.4
-6
-4
-2
0
2
SMD
C
Funnel plot with pseudo 95% confidence limits
se(SMD)
0
.1
.2
.3
.4
-2
0
2
4
SMD
D
Funnel plot with pseudo 95% confidence limits
se(SMD)
0
.1
.2
.3
.4
-8
-6
-4
-2
0
SMD

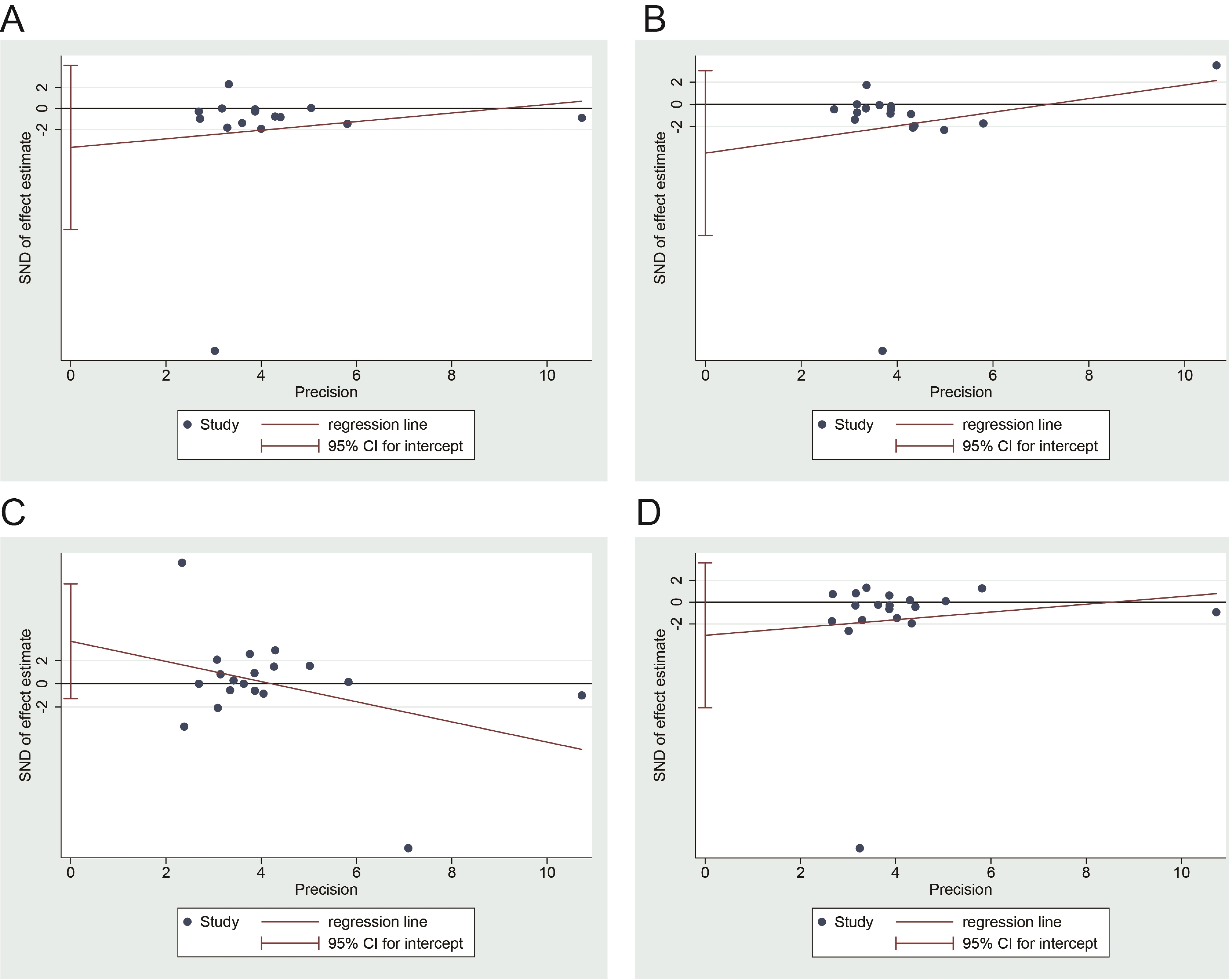

Note: A_metabias_ES_seES, egger(p=0.325); B_metabias_ES_seES, egger(p=0.227)
C_metabias_ES_seES, egger(p=0.136); D_metabias_ES_seES, egger(p=0.348)

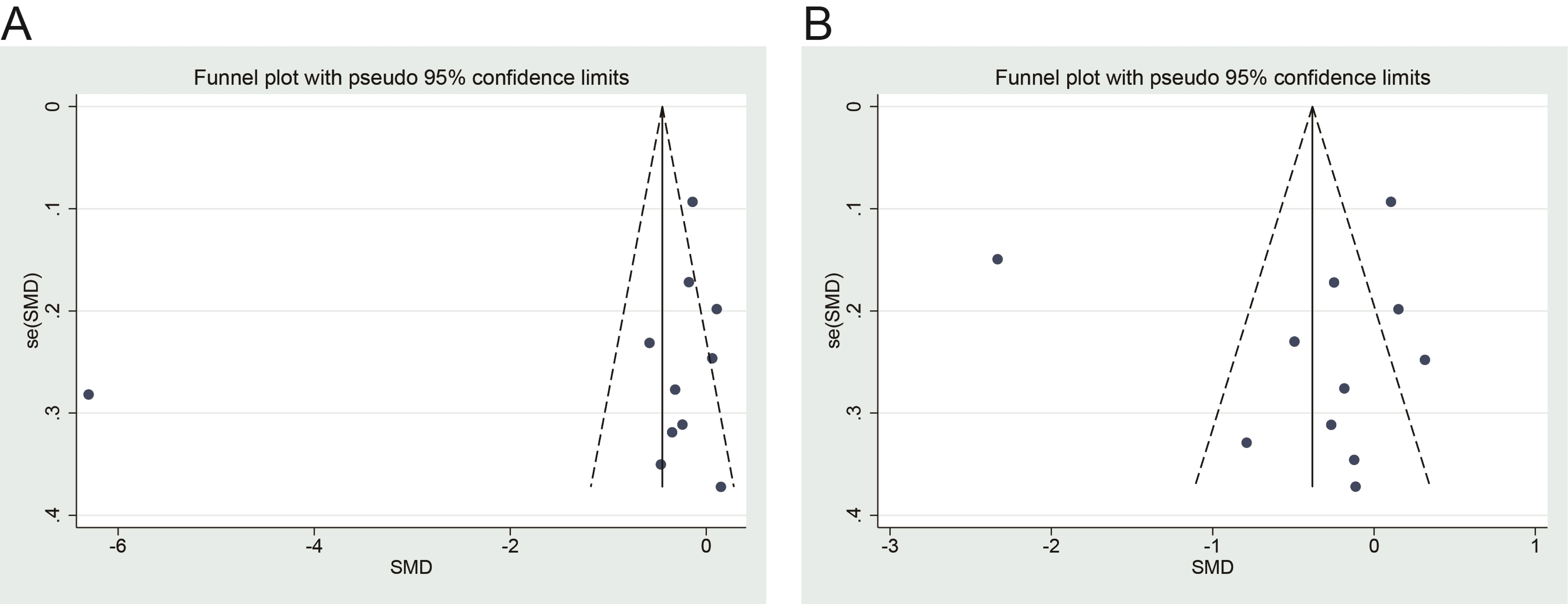
A
Funnel plot with pseudo 95% confidence limits
se(SMD)
0
.1
.2
.3
.4
-6
-4
-2
0
SMD
B
Funnel plot with pseudo 95% confidence limits
se(SMD)
0
.1
.2
.3
.4
-3
-2
-1
0
1
SMD

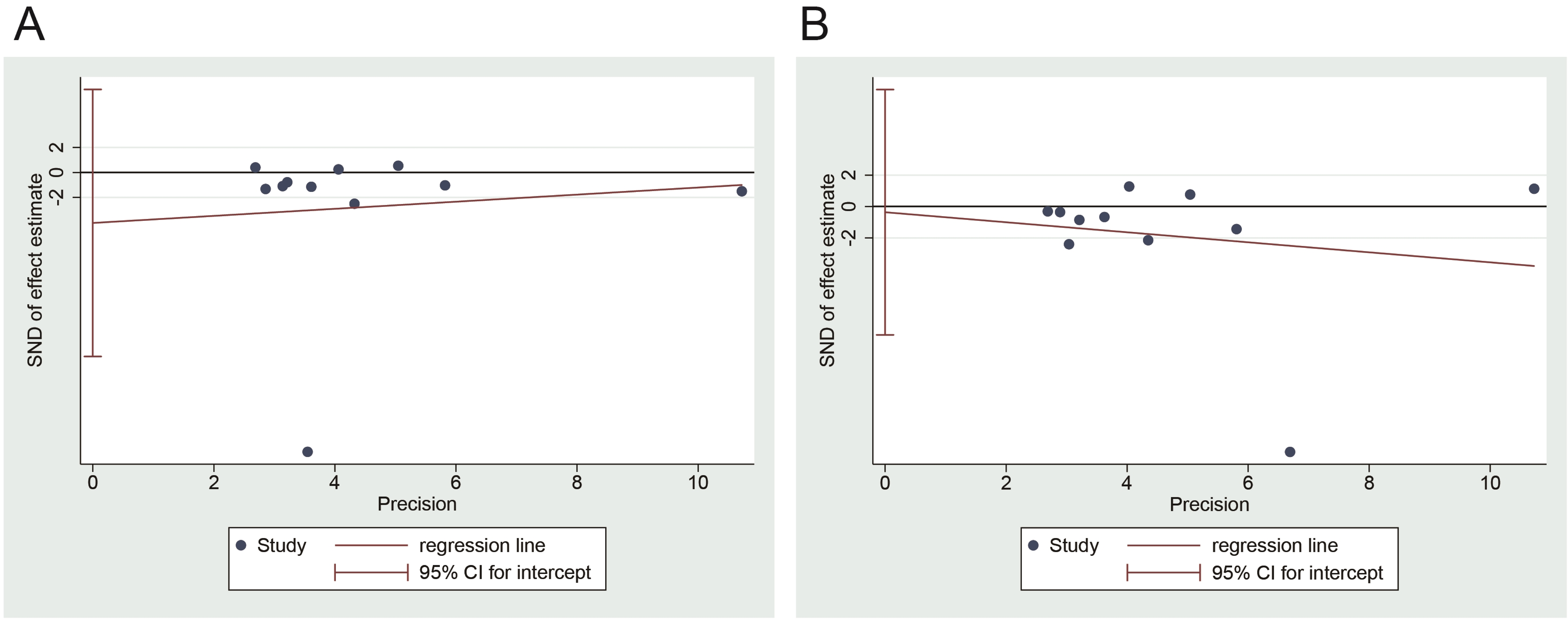

Note: A_metabias_ES_seES, egger(p=0.414); B_metabias_ES_seES, egger(p= 0.919)

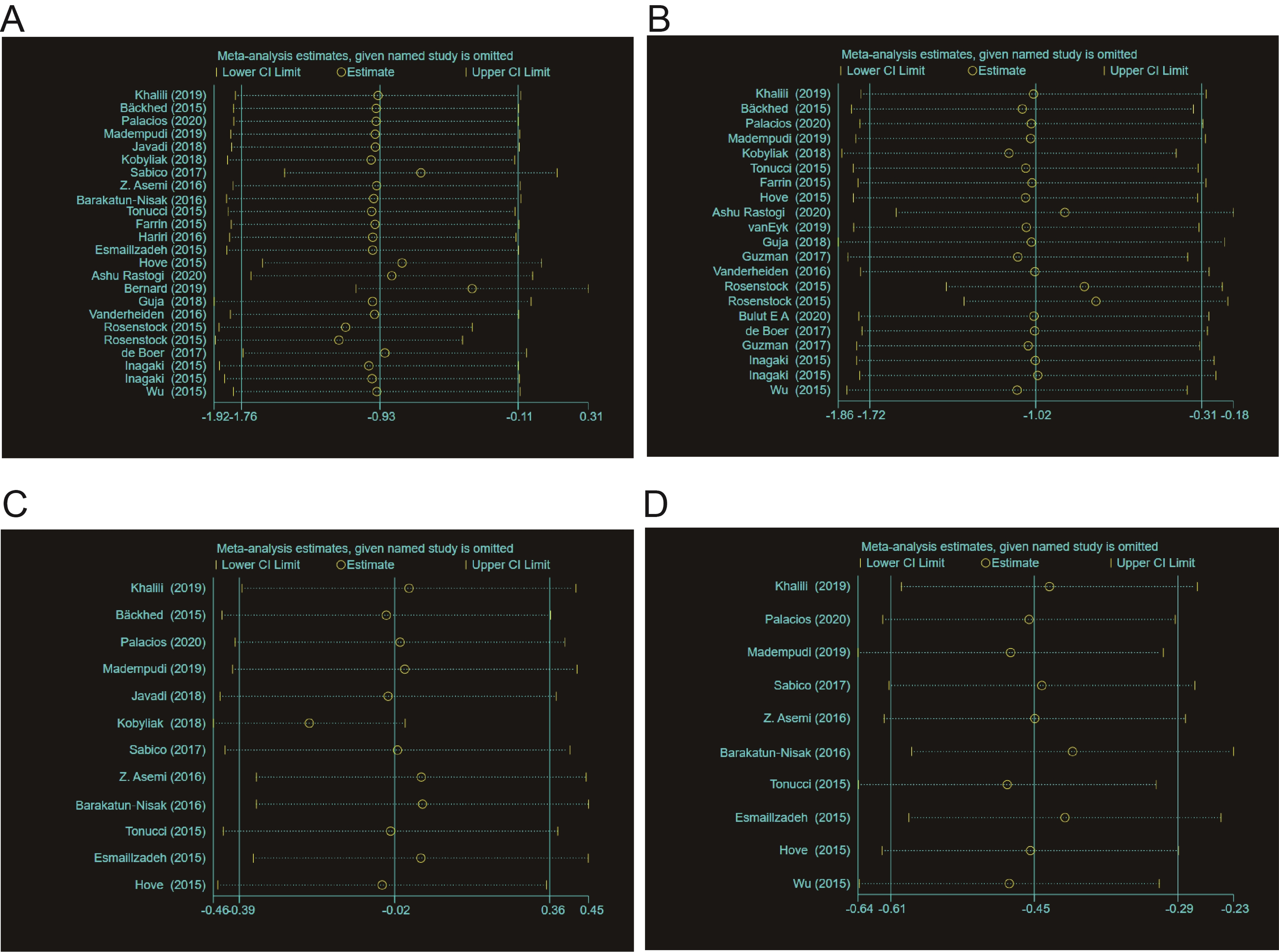
A
Meta-analysis estimates, given named study is omitted
Lower CI Limit
Estimate
Upper CI Limit
Khalili (2019)
Bäckhed (2015)
Palacios (2020)
Madempudi (2019)
Javadi (2018)
Kobyliak (2018)
Sabico (2017)
Z. Asemi (2016)
Barakatun-Nisak (2016)
Tonucci (2015)
Farrin (2015)
Hariri (2016)
Esmaillzadeh (2015)
Hove (2015)
Ashu Rastogi (2020)
Bernard (2019)
Guja (2018)
Vanderheiden (2016)
Rosenstock (2015)
Rosenstock (2015)
de Boer (2017)
Inagaki (2015)
Inagaki (2015)
Wu (2015)
-1.92
-1.76
-0.93
-0.11
0.31
B
Meta-analysis estimates, given named study is omitted
Lower CI Limit
Estimate
Upper CI Limit
Khalili (2019)
Bäckhed (2015)
Palacios (2020)
Madempudi (2019)
Kobyliak (2018)
Tonucci (2015)
Farrin (2015)
Hove (2015)
Ashu Rastogi (2020)
vanEyk (2019)
Guja (2018)
Guzman (2017)
Vanderheiden (2016)
Rosenstock (2015)
Rosenstock (2015)
Bulut E A (2020)
de Boer (2017)
Guzman (2017)
Inagaki (2015)
Inagaki (2015)
Wu (2015)
-1.86
-1.72
-1.02
-0.31
-0.18
C
Meta-analysis estimates, given named study is omitted
Lower CI Limit
Estimate
Upper CI Limit
Khalili (2019)
Bäckhed (2015)
Palacios (2020)
Madempudi (2019)
Javadi (2018)
Kobyliak (2018)
Sabico (2017)
Z. Asemi (2016)
Barakatun-Nisak (2016)
Tonucci (2015)
Esmaillzadeh (2015)
Hove (2015)
-0.46
-0.39
-0.02
0.36
0.45
D
Meta-analysis estimates, given named study is omitted
Lower CI Limit
Estimate
Upper CI Limit
Khalili (2019)
Palacios (2020)
Madempudi (2019)
Sabico (2017)
Z. Asemi (2016)
Barakatun-Nisak (2016)
Tonucci (2015)
Esmaillzadeh (2015)
Hove (2015)
Wu (2015)
-0.64
-0.61
-0.45
-0.29
-0.23

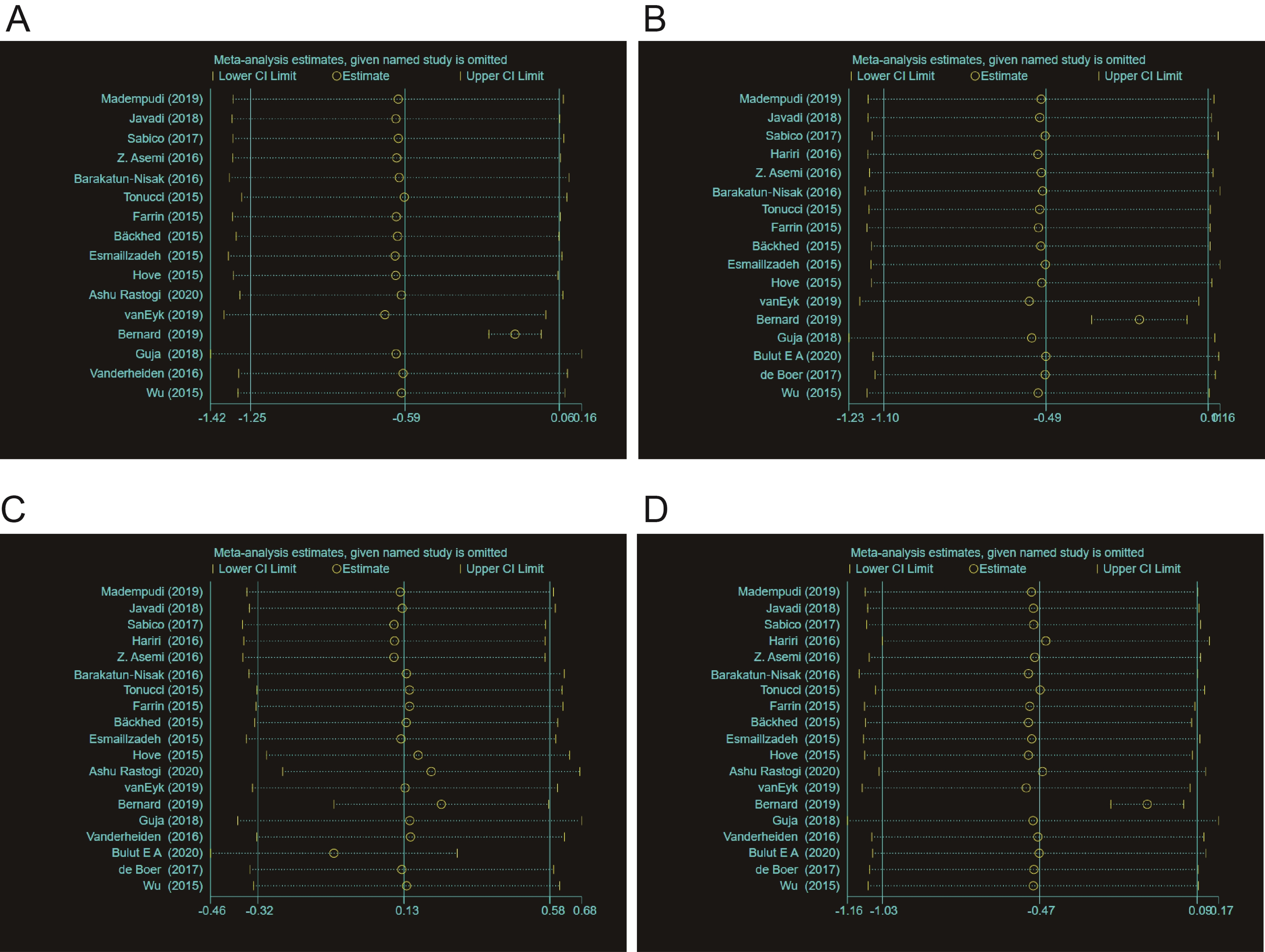

A
Meta-analysis estimates, given named study is omitted
Lower CI Limit
Estimate
Upper CI Limit
Madempudi (2019)
Javadi (2018)
Sabico (2017)
Z. Asemi (2016)
Barakatun-Nisak (2016)
Tonucci (2015)
Farrin (2015)
Bäckhed (2015)
Esmaillzadeh (2015)
Hove (2015)
Ashu Rastogi (2020)
vanEyk (2019)
Bernard (2019)
Guja (2018)
Vanderheiden (2016)
Wu (2015)
-1.42
-1.25
-0.59
0.06
0.16
B
Meta-analysis estimates, given named study is omitted
Lower CI Limit
Estimate
Upper CI Limit
Madempudi (2019)
Javadi (2018)
Sabico (2017)
Hariri (2016)
Z. Asemi (2016)
Barakatun-Nisak (2016)
Tonucci (2015)
Farrin (2015)
Bäckhed (2015)
Esmaillzadeh (2015)
Hove (2015)
vanEyk (2019)
Bernard (2019)
Guja (2018)
Bulut E A (2020)
de Boer (2017)
Wu (2015)
-1.23
-1.10
-0.49
C
Meta-analysis estimates, given named study is omitted
Lower CI Limit
Estimate
Upper CI Limit
Madempudi (2019)
Javadi (2018)
Sabico (2017)
Hariri (2016)
Z. Asemi (2016)
Barakatun-Nisak (2016)
Tonucci (2015)
Farrin (2015)
Bäckhed (2015)
Esmaillzadeh (2015)
Hove (2015)
Ashu Rastogi (2020)
vanEyk (2019)
Bernard (2019)
Guja (2018)
Vanderheiden (2016)
Bulut E A (2020)
de Boer (2017)
Wu (2015)
-0.46
-0.32
0.13
0.58
0.68
D
Meta-analysis estimates, given named study is omitted
Lower CI Limit
Estimate
Upper CI Limit
Madempudi (2019)
Javadi (2018)
Sabico (2017)
Hariri (2016)
Z. Asemi (2016)
Barakatun-Nisak (2016)
Tonucci (2015)
Farrin (2015)
Bäckhed (2015)
Esmaillzadeh (2015)
Hove (2015)
Ashu Rastogi (2020)
vanEyk (2019)
Bernard (2019)
Guja (2018)
Vanderheiden (2016)
Bulut E A (2020)
de Boer (2017)
Wu (2015)
-1.16
-1.03
-0.47
0.09
0.17

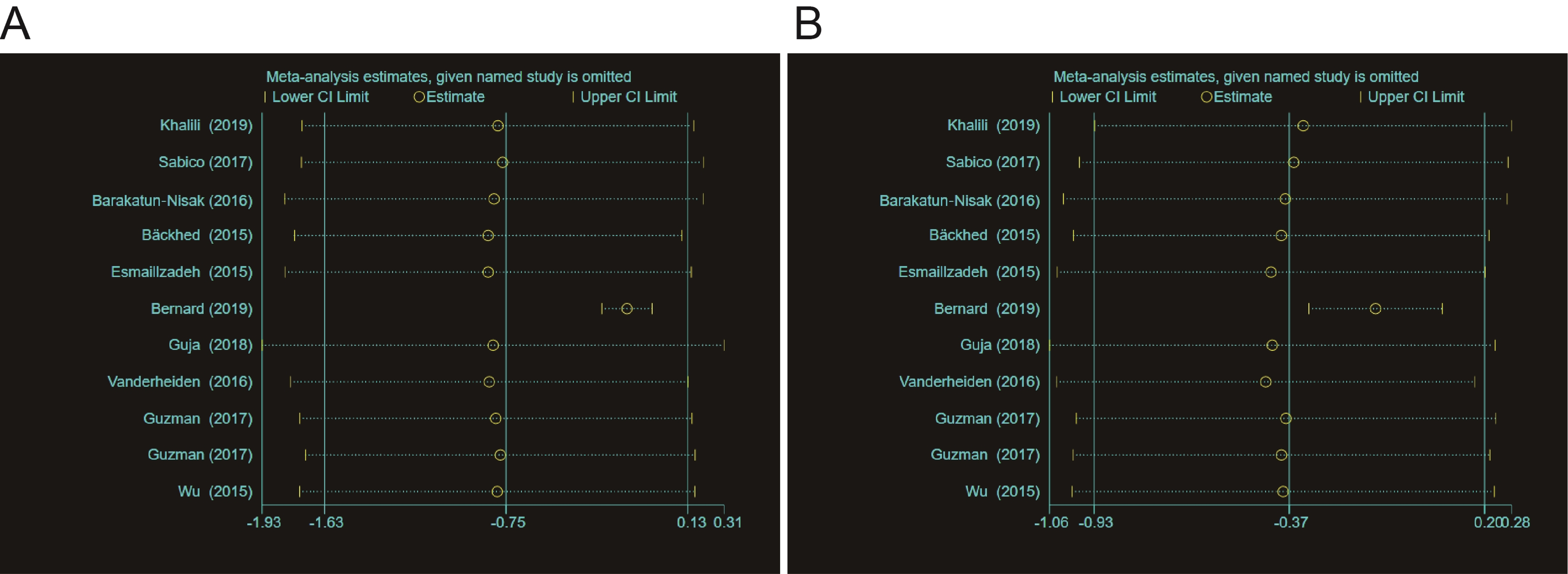

A
Meta-analysis estimates, given named study is omitted
Lower CI Limit
Estimate
Upper CI Limit
Khalili (2019)
Sabico (2017)
Barakatun-Nisak (2016)
Bäckhed (2015)
Esmaillzadeh (2015)
Bernard (2019)
Guja (2018)
Vanderheiden (2016)
Guzman (2017)
Guzman (2017)
Wu (2015)
-1.93
-1.63
-0.75
0.13
0.31
B
Meta-analysis estimates, given named study is omitted
Lower CI Limit
Estimate
Upper CI Limit
Khalili (2019)
Sabico (2017)
Barakatun-Nisak (2016)
Bäckhed (2015)
Esmaillzadeh (2015)
Bernard (2019)
Guja (2018)
Vanderheiden (2016)
Guzman (2017)
Guzman (2017)
Wu (2015)
-1.06
-0.93
-0.37
0.20
0.28
