# Supplementary material for: Comparative analysis of the efficacies of probiotic supplementation and glucose-lowering drugs for the treatment of type 2 diabetes: A systematic review and meta-analysis
Source: Front Nutr. 2022 Jul 18;9:825897. doi: 10.3389/fnut.2022.825897 (PMC9339904; doi:10.3389/fnut.2022.825897)
Supplement: Supplementary Table 1 — The search terms for each database. [file Table_1.docx]

**Sup Table 1.** The search terms for each database.

| No | Database | Search terms |
| --- | --- | --- |
| 1 | Pubmed | (“Type 2 diabetes mellitus”[MsSH Terms] OR “Type 2 diabetes mellitus”[All Fields] OR “Type 2 diabetes”[All Fields] OR “T2D”[All Fields] OR “T2DM”[All Fields] OR “Type 2 diabetes mellitus”[All Fields] AND “glycemia” [MsSH Terms] OR “glucose”[All Fields] OR “fasting blood sugar”[All Fields] OR “FBS”[All Fields] OR “fasting plasma glucose”[All Fields] OR “FPG”[All Fields] OR “Glycosylated hemoglobin A1c”[All Fields] OR “HbA1c”[All Fields] OR “insulin”[All Fields] OR “Homeostasis model assessment- insulin resistance”[All Fields] OR “HOMA-IR”[All Fields] OR “cholesterol” [MsSH Terms] OR “lipids”[All Fields] OR “Total cholesterol”[All Fields] OR “TC”[All Fields] OR “Triglyceride”[All Fields] OR “TG”[All Fields] OR “High density lipoprotein cholesterol”[All Fields] OR “HDL-C”[All Fields] OR “Low density lipoprotein cholesterol”[All Fields] OR “LDL-C”[All Fields] OR “blood pressure”[MsSH Terms] OR “Systolic blood pressure”[All Fields] OR “SBP”[All Fields] OR “Diastolic blood pressure”[All Fields] OR “DBP”[All Fields] OR “probiotic”[MsSH Terms] AND “probiotics”[All Fields] OR “*lactobacillus*”[All Fields] OR “*bifidobacteria*”[All Fields] AND “glucose-lowering drugs”[MsSH Terms] OR “antihyperglycemic agent” [All Fields] OR “thiazolidinedione”[All Fields] OR “TZD”[All Fields] OR “glucagon-like peptide-1 receptor agonists”[All Fields] OR “GLP-1 RA”[All Fields] OR “dipeptidyl peptidase IV inhibitors”[All Fields] OR “DPP-4i”[All Fields] OR “sodium glucose co-transporter 2 inhibitors (SGLT-2i)”[All Fields] OR “SGLT-2i”[All Fields] AND “Randomized Controlled Trial”[MsSH Terms] OR “RCT”[All Fields] |
| 2 | Web of science | (Type 2 diabetes mellitus) AND (glycemia OR glucose OR fasting blood sugar OR fasting plasma glucose OR HbA1c OR insulin OR HOMA-IR) AND (cholesterol OR lipids) AND (blood pressure) AND (probiotic) AND (glucose-lowering drugs OR thiazolidinedione OR glucagon-like peptide-1 receptor agonists OR dipeptidyl peptidase IV inhibitors OR sodium glucose co-transporter 2 inhibitors) AND (Randomized Controlled Trial OR RCT) |
| 3 | Cochrane | (Type 2 diabetes mellitus) AND (glycemia OR glucose OR fasting blood sugar OR fasting plasma glucose OR HbA1c OR insulin OR HOMA-IR) AND (cholesterol OR lipids) AND (blood pressure) AND (probiotic) AND (glucose-lowering drugs OR thiazolidinedione OR glucagon-like peptide-1 receptor agonists OR dipeptidyl peptidase IV inhibitors OR sodium glucose co-transporter 2 inhibitors) AND (Randomized Controlled Trial) |
| 4 | Embase | (Type 2 diabetes mellitus, glycemia, glucose, fasting blood sugar, fasting plasma glucose, HbA1c, insulin, HOMA-IR, cholesterol, lipids, blood pressure, probiotic, glucose-lowering drugs, thiazolidinedione, glucagon-like peptide-1 receptor agonists, dipeptidyl peptidase IV inhibitors, sodium glucose co-transporter 2 inhibitors, Randomized Controlled Trial) |
