# Supplementary material for: Comparative analysis of the efficacies of probiotic supplementation and glucose-lowering drugs for the treatment of type 2 diabetes: A systematic review and meta-analysis
Source: Front Nutr. 2022 Jul 18;9:825897. doi: 10.3389/fnut.2022.825897 (PMC9339904; doi:10.3389/fnut.2022.825897)
Supplement: Supplementary Table 2 — PICOS criteria for inclusion and exclusion of studies. [file Table_2.docx]

**Sup Table 2.** PICOS criteria for inclusion and exclusion of studies.

| Parameter | Defined criteria for current study |
| --- | --- |
| Participants | Type 2 diabetic patients |
| Intervention | Glucose-lowering drugs (thiazolidinedione (TZD), glucagon-like peptide-1 receptor agonists (GLP-1 RA), dipeptidyl peptidase IV inhibitors (DPP-4i), and sodium glucose co-transporter 2 inhibitors (SGLT-2i)); single strain probiotic, or multi-strain probiotic, or probiotic with co-supplements |
| Comparator | Placebo control |
| Outcomes | Fasting blood sugar (FBS), HbA1c, Insulin, homeostasis model assessment of insulin resistance (HOMA-IR), Total cholesterol (TC), triglycerides (TG), high-density lipoprotein cholesterol (HDL-C), low-density lipoprotein cholesterol (LDL-C), systolic blood pressure (SBP), diastolic blood pressure (DBP), hypoglycemia, diarrhea |
| Study design | Randomised controlled human trials (RCTs) |
